# Root Adaptive Responses to Aluminum-Treatment Revealed by RNA-Seq in Two Citrus Species With Different Aluminum-Tolerance

**DOI:** 10.3389/fpls.2017.00330

**Published:** 2017-03-08

**Authors:** Peng Guo, Yi-Ping Qi, Lin-Tong Yang, Ning-Wei Lai, Xin Ye, Yi Yang, Li-Song Chen

**Affiliations:** ^1^Institute of Plant Nutritional Physiology and Molecular Biology, College of Resources and Environment, Fujian Agriculture and Forestry UniversityFuzhou, China; ^2^Institute of Materia Medica, Fujian Academy of Medical SciencesFuzhou, China; ^3^Fujian Provincial Key Laboratory of Soil Environmental Health and Regulation, College of Resources and Environment, Fujian Agriculture and Forestry UniversityFuzhou, China; ^4^The Higher Educational Key Laboratory of Fujian Province for Soil Ecosystem Health and Regulation, College of Resources and Environment, Fujian Agriculture and Forestry UniversityFuzhou, China

**Keywords:** aluminum, *Citrus grandis*, *Citrus sinensis*, organic acid anion secretion, phosphorus homeostasis, RNA-Seq, sulfur metabolism

## Abstract

Seedlings of aluminum (Al)-tolerant *Citrus sinensis* and Al-intolerant *Citrus grandis* were fertigated daily with nutrient solution containing 0 and 1.0 mM AlCl_3_●6H_2_O for 18 weeks. The Al-induced decreases of biomass and root total soluble proteins only occurred in *C. grandis*, demonstrating that *C. sinensis* had higher Al-tolerance than *C. grandis*. Under Al-treatment, *C. sinensis* roots secreted more citrate and malate than *C. grandis* ones; less Al was accumulated in *C. sinenis* than in *C. grandis* leaves. The Al-induced reduction of phosphorus was lesser in *C. sinensis* roots and leaves than in *C. grandis* ones, whereas the Al-induced increase of sulfur was greater in *C. sinensis* roots and leaves. Using RNA-seq, we isolated 1905 and 2670 differentially expressed genes (DEGs) from Al-treated *C. sinensis* than *C. grandis* roots, respectively. Among these DEGs, only 649 DEGs were shared by the two species. Further analysis suggested that the following several aspects conferred *C. sinensis* higher Al-tolerance: (a) Al-treated *C. sinensis* seedlings had a higher external Al detoxification capacity *via* enhanced Al-induced secretion of organic acid anions, a higher antioxidant capacity and a more efficient chelation system in roots; (b) Al-treated *C. sinensis* seedlings displayed a higher level of sulfur in roots and leaves possibly due to increased uptake and decreased export of sulfur and a higher capacity to maintain the cellular phosphorus homeostasis by enhancing phosphorus acquisition and utilization; (c) Cell wall and cytoskeleton metabolism, energy and carbohydrate metabolism and signal transduction displayed higher adaptative responses to Al in *C. sinensis* than in *C. grandis* roots; (d) More upregulated than downregulated genes related to fatty acid and amino acid metabolisms were isolated from Al-treated *C. sinensis* roots, but the reverse was the case for Al-treated *C. grandis* roots. These results provide a platform for further investigating the roles of genes possibly responsible for citrus Al-tolerance.

## Introduction

In neutral or middly acidic soils, aluminum (Al) exists primarily as the forms of insoluble deposits. In acidic soils (pH < 5.0), Al is released from these deposits into soil solution in the forms of Al^3+^, Al(OH)^2+^ and Al(OH)_2_^+^. Micromolar concentration of Al^3+^ is capable of inhibiting root growth, thus impairing water and nutrient uptake and leading to crop yield loss. Thus, Al-toxicity is a major limiting factor for crop productivity on acidic soils (Kochian, 1995; Yang et al., 2013). Citrus, one of the most important fruit crops in the world, are mainly cultivated on acidic and strong acidic soils. Low pH and high Al are the factors responsible for poor growth and shortened lifespan of citrus trees (Lin and Myhre, 1990). What’s worse, soil acidification is occurring rapidly in citrus orchards in the last decade (Li et al., 2015). Therefore, understanding the mechanisms underlying Al-toxicity and Al-tolerance in citrus plants is very important for citrus production.

To tolerate high level of active Al in acidic soils, higher plants have evolved diverse mechanisms for detoxifying externally such as the Al-induced secretion of Al ligands [organic acid (OA) anions, phenolic compounds and phosphate (Pi)] from the roots, efflux of Al, increased pH in the rhizosphere, redistribution of Al and modified cell wall, and internally such as complexation and sequestration of Al. So far, the Al-induced release of OA anions from roots is the best characterized mechanism responsible for the Al-tolerance of higher plants (Yang et al., 2013; Kochian et al., 2015). Genes responsible for the Al-induced secretion of malate [*ALMT1* (*Al-activated malate transporter*)] and citrate [*HvMATE* (*multidrug and toxic compound extrusion*) and *SbMATE*] have been cloned from wheat (*ALMT1*; Sasaki et al., 2004), barely (*HvMATE*; Furukawa et al., 2007) and sorghum (*SbMATE*; Magalhaes et al., 2007). Overexpression of these genes confers Al-tolerance in transgenic sorghum (Magalhaes et al., 2007), *Arabidopsis* (Liu et al., 2009), barely (Delhaize et al., 2004) and wheat (Pereira et al., 2010) plants.

Recently, several Al-tolerance genes involved in the cell wall modification [*STAR1* (*sensitive to Al rhizotoxicity1*) and *STAR2*] and the sequestration of Al [*ALS1* (Al sensitive 1), *ALS3* and *Nrat1* (*Nramp aluminum transporter 1*) and *IREG1* (*IRON REGULATED/ferroportin 1*)] have been identified in model plants rice (*STAR1*, *STAR2* and *Nrat1*; Huang et al., 2009; Li et al., 2014), *Arabidopsis* (*ALS1* and *ALS3*; Larsen et al., 2005, 2007) and buckwheat (*IREG1*) (Yokosho et al., 2016). In addition, several Al-tolerance genes related to other processes that are independent of externall Al detoxification and sequestration of Al have been isolated from model plants, for example, *OsART1* [*Al resistance transcription factor (TF) 1*, a C2H2-type zinc-finger TF] (Yamaji et al., 2009), *AtSTOP1* (*sensitive to proton rhizotoxicity 1*, encoding a zinc finger protein) (Iuchi et al., 2007), *AtWRKY46* (Ding et al., 2013), *OsASR5* (ABA stress and ripening, acting as a TF) (Arenhart et al., 2014), *OsMGT1* [*magnesium (Mg) transporter 1*; Chen Z.C. et al., 2012], *AtMGT1* (Deng et al., 2006) and *OsMGD* [*monogalactosyldiacylglycerol (MGDG) synthase*; Zhang et al., 2016]. All these genes have been demonstrated to confer Al-tolerance in transgenic rice, *Arabidopsis* and tobacco plants *via* overexpression and/or knockout (RNAi) of them.

Gene expression networks unraveled by transcriptomics give us the chance to understand the mechanisms of Al-toxicity and Al-tolerance in higher plants (Chandran et al., 2008; Kumari et al., 2008; Maron et al., 2008; Fan et al., 2014; Wang et al., 2015; Zhou et al., 2015). Recently, a high-throughput sequencing method [RNA sequencing (RNA-Seq)] is developed to analyze the transcriptome prior to the sequencing of the genome. It provides an opportunity for large-scale and simultaneous estimation of gene abundances and identification of new genes (Grabherr et al., 2011). RNA-seq has been applied to investigate Al-responsive genes in several higher plants including rice (Arenhart et al., 2014), *Anthoxanthum odoratum* (Gould et al., 2015), buckwheat (*Fagopyrum tataricum*) (Yokosho et al., 2014), *Hydrangea macrophylla* (Chen et al., 2015). Using the method, many candidate genes possibly responsible for Al-tolerance have been identified in higher plants. However, most of these researches have focused on herbaceous plants and Al-accumulating plants. Limited data are available on Al-induced alterations of gene expression profiles in non-Al-accumulating woody plants (Brunner and Sperisen, 2013).

In China, citrus are cultivated commercially in acidic and strong acidic soils and are apt to suffer from high Al and low pH (Xu and Ji, 1998; Li et al., 2015). Previously, we used Al-tolerant *Citrus sinensis* and Al-intolerant *Citrus grandis* seedlings and comparatively investigated citrus Al-toxicity and Al-tolerance at physiological and protein levels (Yang L.T. et al., 2011; Jiang et al., 2015; Li et al., 2016). In addition, qRT-RCR analysis showed that the coordinated expression regulation of genes related to alternative glycolytic pathways, phosphorus (P) scavenging and recycling in *C. sinensis* and *C. grandis* roots played a role in citrus tolerance to Al and/or P-deficiency (Yang et al., 2012). In this study, we extended the knowledge on citrus Al-toxicity and Al-tolerance through investigating the Al-induced alterations of transcriptomics in roots of the two citrus species with different Al-tolerance using RNA-Seq. Through analysis of the Al-responsive genes, we found some candidate genes possibly responsible for citrus Al-tolerance.

## Materials and Methods

### Plant Materials

Seedling culture and Al treatments were carried out according to Zhou et al. (2015) with some modifications. Five-weeks after sprouting, uniform seedlings of ‘Shatian pummelo’ [*Citrus grandis* (L.) Osbeck] and ‘Xuegan’ [*Citrus sinensis* (L.) Osbeck] were transplanted to 6 L pots (two seedlings per pot) filled with clean river sand, then cultivated in a greenhouse with natural photoperiod at Fujian Agriculture and Forestry University throughout the trial period. Six weeks after transplanting, each pot was irrigated daily with nutrition solution containing 1 mM KNO_3_, 1 mM Ca(NO_3_)_2_, 0.1 mM KH_2_PO_4_, 0.5 mM MgSO_4_, 10 μM H_3_BO_3_, 2 μM MnCl_2_, 2 μM ZnSO_4_, 0.5 μM CuSO_4_, 0.065 μM (NH_4_)_6_Mo_7_O_24_ and 20 μM Fe-EDTA, 0 (control, -Al) or 1.0 mM (+Al) AlCl_3_●6H_2_O for 18 weeks until the sand was saturated. The pH of the solution was adjusted to 4.1 – 4.2 with HCl or NaOH. At the end of the experiment, approximately 5-mm-long root tips from new white roots were excised, immediately frozen in liquid nitrogen, then stored at -80°C until they were used for RNA and total soluble protein extraction. The remaining seedlings that were not sampled were used to measure biomass, root OA anion secretion, and root and leaf Al, P, and sulfur (S) concentrations.

### Seedling DW, S, Al and P in Root and Leaves, and Total Soluble Proteins in Roots

At the end of this experiment, 10 seedlings per treatment (one seedling per pot) were collected and divided into roots, stems and leaves. Their DW was measured after being dried at 70°C for 48 h. There were 10 replicates per treatment.

For the assays of S, Al and P in roots and leaves, fully expanded mature (about 7-week-old) leaves (midribs and petioles removed) and fibrous roots were collected after the seedlings were washed thoroughly with tap water and given a final rinsing with deionized water, then dried at 70°C for 48 h. These dried samples were ground to pass a 40-mesh sieve. S was assayed with the vario MAX cube CNS analyzer (Elementar Analysensysteme GmbH, Hanau, Germany). Al and P concentrations were measured according to Hsu (1963) and Ames (1966), respectively after the powdered samples were digested in a mixture of HNO_3_ : HClO_4_ (Yang et al., 2012). There were four replicates per treatment.

Root total soluble proteins were extracted with 50 mM Na_2_HPO_4_-KH_2_PO_4_ (pH 7.0) and 5% (w/v) insoluble polyvinylpyrrolidone. Total soluble proteins in the extract were assayed according to Bradford (1976). There were four replicates per treatment.

### Collection and Assay of Root Exudates

Root exudates were collected according to Yang L.T. et al. (2011). At the end of this experiment, about 5-mm-long root apices from new white roots of *C. sinensis* and *C. grandis* seedlings treated with or without 1 mM Al were excised. Ten-twelve excised root apices were collected in Petri dishes containing 5 mL of control solution (0.5 mM CaCl_2_, pH 4.1–4.2). After three rinses with 5 mL of control solution (each for 20 min), the root apices were transferred to 2 mL centrifuge tubes containing 1 mL of control solution in the presence or absence of 0.5 mM AlCl_3_●6H_2_O (pH 4.1–4.2). The tubes were placed vertically on a shaker (200 rpm) at dark. Citrate and malate secreted from roots were assayed according to Yang L.T. et al. (2011) as described above after 12 or 24 h treatment, respectively.

### RNA Extraction, cDNA Preparation and RNA-Seq

Equal amounts of frozen roots from five seedlings (one seedling per pot) were pooled as one biological replicate. There were two biological replicates for each treatment. Total RNA was independently extracted twice from ca. 100 – 200 mg frozen control and +Al roots of *C. sinensis* and *C. grandis* seedlings using Recalcirtant Plant Total RNA Extraction Kit (Centrifugal column type, Bioteke Corporation, China) following the manufacturer’s instructions. The integrity and quality of total RNA were checked by 1% (w/v) agarose gel electrophoresis and spectrophotometer at 260 and 280 nm. Only these RNA samples that had a 260 nm/280 nm absorbance ratio of between 1.8 and 2.0 were used for subsequent analyses. Ten microgram (500 ng/μL) of high quality total RNA per sample was shipped to the Genedenovo Biotechnology Corporation (Guangzhou, China) for deep sequencing and generation of datasets. Briefly, mRNA, which was isolated from approximately 5 μg of total RNA, was enriched by using magnetic beads with oligo(dT) (Qiagen, Valencia, CA, USA). The resulting mRNA was then fragmented into short fragments (200 nt) and converted to the first-strand cDNA using random hexamer-primers. The second-strand cDNA was synthesized using RNase H and DNA polymerase I, and purified using a QIAquick PCR extraction kit, then end-repaired with a base A tail adding to 3′ ends. Sequencing adapters were connected to the end of the double-stranded cDNA (200 ± 25 bp) for PCR amplification. Sequencing of the double-stranded cDNAs was carried out on HiSeq^TM^ 2000 device (Illumina Inc., San Diego, CA, USA) using the paired-end technology in a single run. Illumina GA Pipeline (version 1.6) was used to perform the original image process to sequences, base-calling and quality value calculation, in which 125 bp paired-end reads were obtained (Li et al., 2013).

### RNA-Seq Reads Mapping, Transcript Assembly and Analysis of Gene Expression

Clean reads were obtained by removing low quality sequences, reads containing adapter and reads containing ploy-N. In the mapping process, the software Bowtie aligner (Langmead et al., 2009) was used to delete the reads mapping to ribosomal RNA database. The remaining reads were mapped to the *C. sinensis* genome^1^ using the software TopHat2 (Kim et al., 2013), and assembled by Cufflinks (Trapnell et al., 2010) and RABT (reference annotation based transcripts; Roberts et al., 2011).

The gene expression level was calculated by FPKM (fragments per kilobase of transcript per million mapped reads) method (Mortazavi et al., 2008). Differentially expressed analysis was performed using EdgeR (version 3.0.0, R version2.1.5). Genes that had a FDR (false discovery rate) ≤0.05 and an absolute value of the log2 ratio ≥ 1 were considered as differentially expressed. Their functional categories were assigned according to Uniprot^2^, KEGG^3^ and the gene ontology^4^ databases.

### qRT-PCR Analysis

Root total RNA was extracted as described above. There were three biological replicates per treatment. Equal amounts of frozen roots from five seedlings (one seedling per pot) were mixed as one biological replicate. The replicate used for RNA-seq and qRT-PCR was not the same. Genes specific primers were designed using Primier version 5.0 (Premier Biosoft International, Palo Alto, CA, USA). The sequences of the Forward and Reverse primers were listed in Supplementary Table S1. qRT-PCR was carried out following the method of Zhou et al. (2013). For the normalization of gene expression and reliability of qRT-PCR data, three citrus genes [i.e., *actin* (JN191387), *β-tublin* (JN580571) and *polyubiquitin* (GU362416)] were selected as internal standards and the roots from -Al seedlings were used as reference sample, which was set to 1. Each sample was run in two technical replicates.

### Experimental Design and Statistical Analysis

There were 20 pots (40 seedlings) per treatment in a completely randomized design. Experiments were performed with 2–10 replicates. Results represented the mean ± SD. Differences among four treatment combinations (two Al levels × two species) were analyzed by ANOVA tests. Four means were separated by the Duncan’s new multiple range test at *P* < 0.05.

## Results

### Biomass, Al, P and S in Roots and Leaves, and Total Soluble Proteins in Roots

**FIGURE 1:**
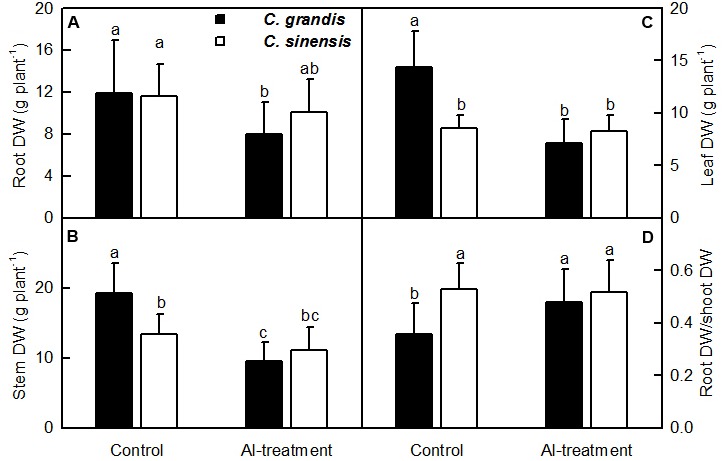
**Effects of Al-treatment on root** **(A)**, stem **(B)** and leaf **(C)** DW, and root DW/shoot DW ratio **(D)** of *C. grandis* and *C. sinensis* seedlings. Bars represent means ± SD (*n* = 10). Different letters above the bars indicate a significant difference at *P* < 0.05.

Al-treatment reduced *C. grandis* root, stem and leaf DW. Root DW decreased to a lesser extent than shoot (stem + leaf) DW when exposed to Al, thus leading to a greater ratio of root DW to shoot DW in *C. grandis* seedlings. However, Al-treatment did not significantly alter all the four parameters in *C. sinensis* seedlings (**Figure 1**).

**FIGURE 2:**
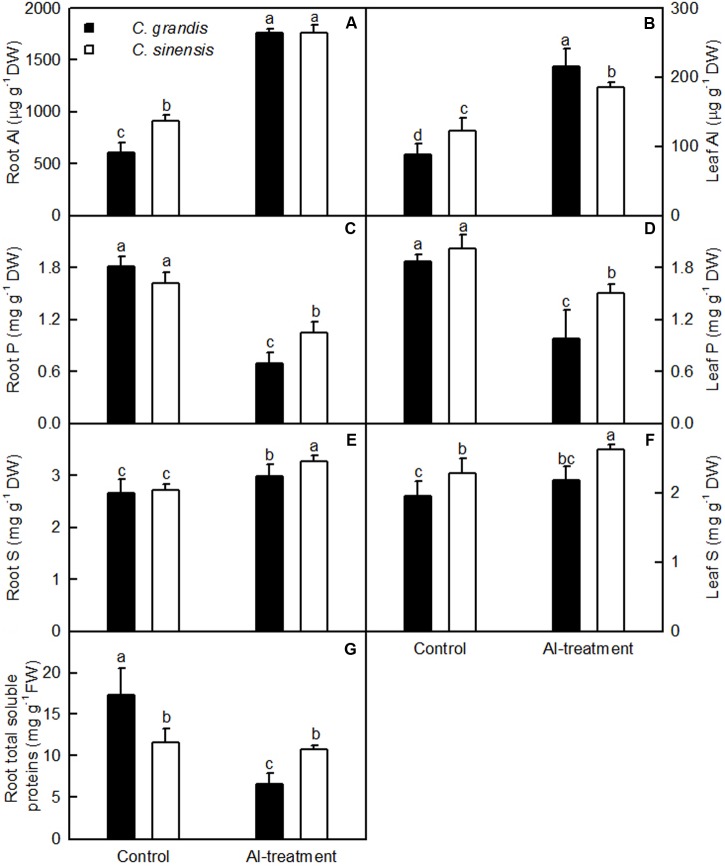
**Effects of Al-treatment on Al** **(A,B)**, P **(C,D)** and S **(E,F)** concentrations in *C. sinensis* and *C. grandis* roots **(A,C,E)** and leaves **(B,D,F)**, and total soluble protein concentration in *C. sinensis* and *C. grandis* roots **(G)**. Bars represent means ± SD (*n* = 4). Different letters above the bars indicate a significant difference at *P* < 0.05.

Al concentration was higher in *C. sinensis* roots and leaves than in *C. grandis* roots and leaves in the absence of Al, but was lower in Al-treated *C. sinensis* leaves than in Al-treated *C. grandis* leaves. No significant difference was found in Al level between +Al *C. grandis* and *C. sinensis* roots (**Figures 2A,B**). Al-treatment reduced P level in *C. sinensis* and *C. grandis* roots and leaves. Root and leaf P concentration did not differ between *C. grandis* and *C. sinensis* in the absence of Al, but was lower in *C. grandis* than in *C. sinensis* under Al-treatment (**Figures 2C,D**). Al-treatment increased S level in *C. sinensis* roots and leaves and *C. grandis* roots, but did not significantly affect its level in *C. grandis* leaves. S concentration was higher in *C. sinensis* roots and leaves than in *C. grandis* roots and leaves at each given Al level. The exception was that root S level was similar between the two species at the absence of Al (**Figures 2E,F**).

Al-treatment lowered total soluble protein concentration only in *C. grandis* roots. Total soluble protein level was higher in *C. grandis* roots than in *C. sinensis* roots at the absence of Al, but the revers was the case under Al-treatment (**Figure 2G**).

### RNA-Seq, *De novo* Assembly of the Transcripts and Annotation

Eight libraries were constructed and sequenced, including two biological replicates for control (control 1 and control 2) and Al-treated (Al-treatment 1 and Al-treatment 2) *C. sinensis* and *C. grandis* roots. The numbers of raw reads generated from each library ranged from 23,472,150 to 29,474,312. The percentages of clean reads and Q20 (sequencing error rates lower than 1%) were more than 98 and 95%, respectively (**Table 1**). Here, 75.2–83.8% of the clean reads were mapped uniquely to the *C. sinensis* genome, and only a small proportion of them were mapped multiply to the genome (**Table 2**). Similar results have been obtained on *C. grandis* fruits (Guo et al., 2015). The number of known transcripts generated from reference genomes ranged from 20,846 to 21,953, which accounted for 70.3–74.0% of the number of annotated genes in the genome. The number of known genes in the four groups (when mixed the two replicates to four groups: control *C. sinensis*, +Al *C. sinensis*, control *C. grandis* and +Al *C. grandis*) accounted for 73.8–76.3% of the annotated genes in the genome (**Table 2**).

**Table 1:** Summary of the RNA-Seq data collected from control and Al-treated roots of *C. sinensis* and *C. grandis*.

Sample andtreatment	Raw reads	Clean reads (%)	Read length	Adapter (%)	Low quality (%)	Poly A (%)	*N* (%)	Q20 %	GC %
***C. sinensis***									
Control 1	29474312	29038964 (98.52%)	125 + 125	199137 (1.35%)	18537 (0.13%)	0 (0%)	0 (0%)	95.64	45.12
Control 2	28450848	28024122 (98.50%)	125 + 125	194415 (1.37%)	18948 (0.13%)	0 (0%)	0 (0%)	95.47	45.02
Al-treatment 1	26476012	26081448 (98.51%)	125 + 125	179110 (1.35%)	18172 (0.14%)	0 (0%)	0 (0%)	95.41	45.04
Al-treatment 2	24254284	23892372 (98.51%)	125 + 125	164453 (1.35%)	16503 (0.14%)	0 (0%)	0 (0%)	95.43	45.14
***C. grandis***									
Control 1	26043782	25665568 (98.55%)	125 + 125	170586 (1.31%)	18521 (0.14%)	0 (0%)	0 (0%)	95.24	45.27
Control 2	26937382	26560774 (98.60%)	125 + 125	170882 (1.27%)	17422 (0.13%)	0 (0%)	0 (0%)	95.59	45.52
Al-treatment 1	23472150	23203560 (98.86%)	125 + 125	116640 (0.99%)	17655 (0.15%)	0 (0%)	0 (0%)	95.26	46.63
Al-treatment 2	28606284	28187340 (98.54%)	125 + 125	192291 (1.35%)	17181 (0.12%)	0 (0%)	0 (0%)	95.69	44.46


*N, unknown base rates higher than 10%; Q20, Sequencing error rates lower than 1%.*

**Table 2:** Summary of clean reads and genes mapped to the reference genome from control and Al-treated roots of *C. sinensis* and *C. grandis*.

Sample andtreatment	Total reads	Total mapped	Multiple mapped	Unique mapped	Known genes	Known genes in two samples	Novel genes	Total genes
***C. sinensis***								
Control 1	29038964	24762847 (85.27%)	265639 (1.83%)	24231569 (83.45%)	21886 (73.8%)	22625(76.3%)	453	22339
Control 2	28024122	23911090 (85.32%)	250294 (1.79%)	23410502 (83.54%)	21895 (73.8%)		442	22337
Al-treatment 1	26081448	22299231 (85.50%)	231529 (1.78%)	21836173 (83.72%)	21953 (74.0%)	22629(76.3%)	457	22410
Al-treatment 2	23892372	20453417 (85.61%)	212055 (1.78%)	20029307 (83.83%)	21789 (73.5%)		454	22243
***C. grandis***								
Control 1	25665568	19822398(77.23%)	193082 (1.50%)	19436234 (75.73%)	21171 (71.4%)	21871(73.8%)	428	21599
Control 2	26560774	20540684 (77.33%)	219723 (1.65%)	20101238 (75.68%)	21078 (71.1%)		418	21496
Al-treatment 1	23203560	18549555 (79.94%)	246157 (2.12%)	18057241 (77.82%)	20846 (70.3%)	21932(74.0%)	415	21261
Al-treatment 2	28187340	21615136 (76.68%)	208814 (1.48%)	21197508 (75.20%)	21377 (72.1%)		425	21802


### Al-Induced Secretion of Malate and Citrate

**FIGURE 3:**
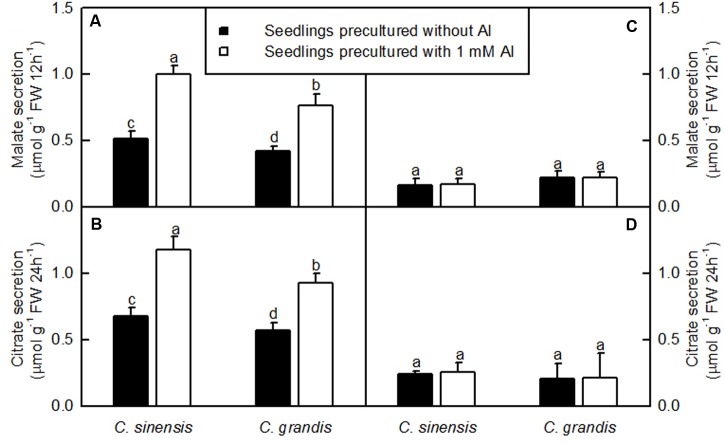
**Al-induced secretion of malate** **(A,C)** and citrate **(B,D)** by excised roots from *C. sinensis* and *C. grandis* seedlings treated with 0 and 1 mM Al for 18 weeks. Malate and citrate secretion from excised roots was determined after 12 or 24 h treatment, respectively in 0.5 mM CaCl_2_ + 0.5 mM AlCl_3_●6H_2_O **(A,B)** or 0.5 mM CaCl_2_ **(C,D)** solution, pH 4.1–4.2. Bars represent means ± SD (*n* = 4). Different letters above the bars indicate a significant difference at *P* < 0.05.

The Al-induced secretion of malate and citrate from +Al excised roots was higher than from -Al excised ones. Excised *C. sinensis* roots secreted more citrate and malate than *C. grandis* ones when exposed to Al (**Figure 3**).

### DEGs between Control and Al-Treated Roots

**FIGURE 4:**
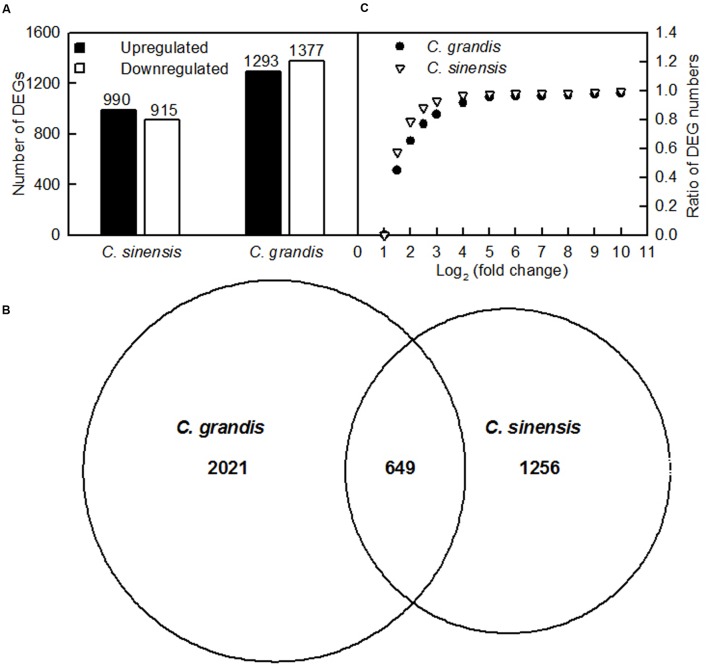
**DEGs identified in Al-treated *C. grandis* and *C. sinensis* roots.** **(A)** upregulated and downregulated genes in Al-treated *C. grandis* and *C. sinensis* roots, **(B)** venn diagram analysis of Al-responsive genes in roots of the two citrus species, **(C)** fold change pattern of DEGs in roots of the two citrus species.

Here, the DEGs between control and Al-treated roots were identified with an absolute value of the log2 ratio ≥ 1 and a threshold of FDR ≤ 0.05. Based on the two criteria, we obtained 1293 upregulated and 1370 downregulated, and 990 upregulated and 915 downregulated DEGs from Al-treated *C. grandis* and *C. sinensis* roots, respectively (**Figure 4A** and Supplementary Tables S2, S3).

As shown in **Figure 4B** and Supplementary Table S4, we isolated a total of 3926 DEGs from +Al *C. sinensis* and *C. grandis* roots. Among these DEGs, only 649 DEGs were shared by the two species. The changes in low-P-responsive genes, TFs and genes involved in cellular transport, S transport and metabolism, antioxidation and detoxification, polysaccharide, cell wall and cytoskeleton metabolism, carbohydrate and energy metabolism, protein and amino acid metabolism, lipid metabolism, and signal transduction and hormone metabolism also differed between *C. sinensis* and *C. grandis* roots in response to Al (Supplementary Tables S5–14). Great differences existed in Al-induced alterations of gene expression profiles between the two citrus species. As shown in **Figure 4C**, 78.7% of DEGs had an absolute value of log2 the ratio < 2 in +Al *C. sinensis* roots, while only 65.0% of DEGs had an absolute value of log2 the ratio < 2 in +Al *C. grandis* ones, implying that wider variation of gene expression occurred in +Al *C. grandis* roots than in +Al *C. sinensis* roots.

### qRT-PCR Validation of RNA-Seq Expression Data

**FIGURE 5:**
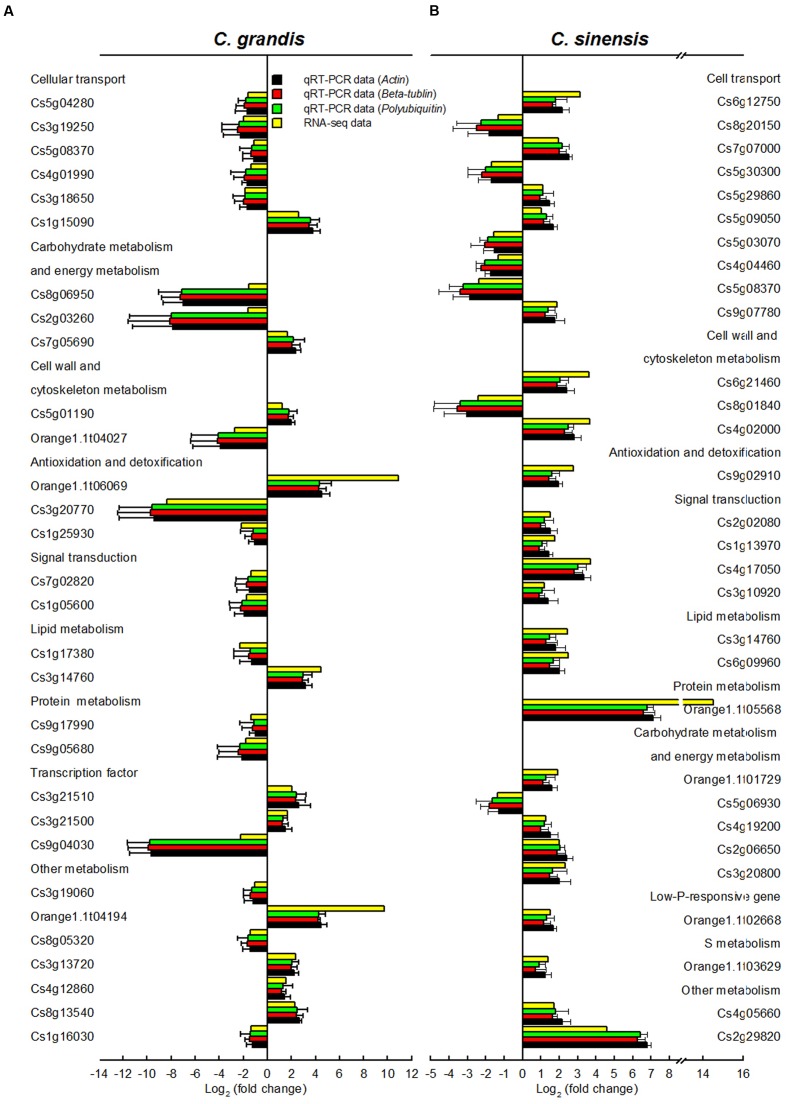
**Relative expression levels of DEGs from Al-treated *C. grandis*** **(A)** and *C. sinensis* **(B)** roots. Bars represent means ± SD (*n* = 3). Three citrus genes [i.e., *β-tubulin* (JN580571), *actin* (JN191387) and *polyubiquitin* (GU362416)] were selected as the internal standards and the roots from control seedlings were used as reference sample, which was set to 1.

In order to validate RNA-Seq expression data, 60 DEGs selected randomly from *C. grandis* (30) and *C. sinensis* (30) roots were used for qRT-PCR analysis. Three genes were selected as the internal standards. The expression patterns of all 60 DEGs revealed by qRT-PCR were in agreement with the RNA-seq expression data, demonstrating that the RNA-seq data were reliable (**Figure 5**).

## Discussion

### *Citrus sinensis* Displayed Higher Al-Tolerance than *C. grandis*

Al-treatment led to decreased root, stem and leaf DW, and increased ratio of root DW/shoot DW in *C. grandis* seedlings, but did not affect the four parameters in *C. sinensis* seedlings (**Figure 1**). Also, Al-induced reduction of the total soluble protein level only occurred in *C. grandis* roots (**Figure 2G**). Based on these results, we concluded that *C. sinensis* seedlings were more tolerant to Al than *C. grandis* ones. This is also supported by our data that less DEGs was identified in +Al *C. sinensis* roots than in +Al *C. grandis* roots (**Figure 4A**). Similar result has been obtained on Al-treated maize roots (Maron et al., 2008). However, Jiang et al. (2015) obtained more Al-responsive proteins in *C. sinensis* than in *C. grandis* ones. The discrepancy between the Al-induced alterations of root gene expression patterns and protein profiles means that post-translational modifications (PTMs) might influence protein abundance.

The tolerance of higher plants to Al is related not only to low Al uptake, but also to relatively little Al transport from roots to shoots (leaves) (Yang L.T. et al., 2011). Our results indicated that less Al was transported from roots to shoots (leaves) of *C. sinensis* seedlings when exposed to Al relative to *C. grandis* seedlings (**Figures 2A,B**), thus contributing to the Al-tolerance of *C. sinensis* seedlings.

The Al-induced secretion of OA anions from roots is considered as a major mechanism of Al-tolerance in higher plants (Yang et al., 2013; Kochian et al., 2015). We found that *C. sinensis* roots secreted more citrate and malate than *C. grandis* ones when exposed to Al (**Figure 3**), demonstrating that the Al-induced secretion of citrate and malate played a role in the higher Al-tolerance of *C. sinensis via* external detoxification. This is also supported by our finding that Al concentration was lower in +Al *C. sinensis* than in +Al *C. grandis* leaves (**Figure 2B**).

### Genes Related to Cellular Transport

Many transport-related DEGs were isolated from +Al *C. grandis* and *C. sinensis* roots (Supplementary Table S5). They included genes encoding malate and citrate transporters [ALMTs, MATEs and ferric reductase defective 3b (FRD3b)], ATPases, ion transporters, cyclic nucleotide-gated ion channels (CNGCs), ammonium transporters and amino acid transporters. Here, we obtained two upregulated and one downregulated, and two upregulated and three downregulated *ALMTs* from +Al *C. grandis* and *C. sinensis* roots, respectively, demonstrating the possible involvement of *ALMTs* in the Al-induced secretion of malate from citrus roots. However, the Al-induced secretion of malate from citrus roots could not be explained by the activation of *ALMTs* alone because *C. sinensis* roots secreted more malate than *C. grandis* roots when exposed to Al (**Figures 3A,B**). By contrast, we identified four upregulated genes (three *MATEs* and one *FRD3b*) involved in citrate secretion and one down-regulated *MATE* from +Al *C. sinensis* roots, but only one up-regulated *FRD3b* and one down-regulated *MATE* from +Al *C. grandis* roots, which agrees with our report that the Al-induced secretion of citrate from *C. sinensis* roots was higher than from *C. grandis* roots (**Figures 3C,D**).

Internal detoxification of Al is mainly achieved *via* active transport and the sequestration of Al into the vacuoles (Kochian et al., 2015). Overexpression of *FeIREG1* from buckwheat conferred Al-tolerance in transgenic *Arabidopsis* plants possibly through sequestrating Al into root vacuoles (Yokosho et al., 2016). Thus, the upregulation of *Fe regulated 1 protein* in +Al *C. grandis* and *C. sinensis* roots might be involved in citrus Al-tolerance. In rice, Al uptake is mediated by a plasma-membrane bound Nramp (natural resistance-associated macrophage protein) family Al transport protein, OsNarat1, which removes Al from the apoplast and sequester it into the vacuoles of roots by concerting with a vacuolar ABC-transporter, OsALS1 (Li et al., 2014). Here, we found that *metal transporter Nramp6* was upregulated in +Al *C. grandis* roots. In addition, the expression of many genes encoding aquaporins, ABC-transporters and major facilitator superfamily proteins (MFSs) was altered in +Al C. *grandis* and *C. sinensis* roots, demonstrating the possible involvement of these genes in Al sequestration.

### Low-P-Responsive Genes

Studies showed that P-deficiency was the key cause of the Al-induced growth inhibition in plants (Quartin et al., 2001), and that P supply could alleviate plant Al-toxicity (Jiang et al., 2009). Here, we obtained 16 upregulated and six downregulated, and 18 upregulated and three downregulated low-P-responsive genes from +Al *C. grandis* and *C. sinensis* roots, respectively, demonstrating that the upregulation of low-P-responsive genes was greater in +Al *C. sinensis* roots (Supplementary Table S6). Induction of acid phosphatases (APs) is an adaptive stragety of plants to P-deficiency. Purple APs (PAPs, the largest group of non-specific APs) play a vital role in Pi recycling and scavenging in P-deficient plants (Liu et al., 2016). Maron et al. (2008) reported that the Al-induced upregulation of root *PAPs* was greater in Al-tolerant maize genotype than in Al-sensitive ones. Here, we isolated six upregulated and one downregulated *PAPs* from +Al *C. sinensis* roots, and one downregulated *phosphatidic acid phosphatase-related protein*, five upregulated and one downregulated *PAPs* from +Al *C. grandis* roots. Also, we isolated one upregulated *alkaline-phosphatase-like family protein isoform 1* from +Al *C. sinensis* roots, which agrees with the report that an alkaline phosphatase was induced in P-deficient *Phaseolus vulgaris* roots (Morales et al., 2012). Duff et al. (1989) observed that NADP-glyceraldehyde-3-phosphate dehydrogenase (NADP-G3PDH) specific activity was increased in P-starved *Brassica nigra* suspension cells, suggesting that the enzyme circumvented Pi-dependent NAD-G3PDH and phosphoglycerate kinase. Characterization of the *Arabidopsis* glycerophosphodiester phosphodiesterase (GDPD) family showed that plastid-localized AtGDPD1 played a role in maintaining cellular Pi homeostasis by releasing Pi from phospholipids in P-starved plants (Cheng et al., 2011). Ribonucleases play a role in the remobilization of Pi from RNA under P-limited conditions (Bariola et al., 1994). Caparrós-Martín et al. (2013) showed that that AtSgpp (At2g38740) gene encoding haloacid dehalogenase-like hydrolase (HAD) might play a role in the homeostatic balance of Pi in the cell. Here, we isolated three and one upregulated HAD genes from +Al *C. sinensis* and *C. grandis* roots, respectively. Also, we obtained two downregulated (Cs2g04480 and Cs9g18560) and two upregulated (Cs3g27660 and Cs7g19830) genes involved in Pi transport from +Al *C. grandis* roots, but only one downregulated (Cs9g18560) and two upregulated (Cs7g19830 and Cs5g29860) genes from +Al *C. sinensis* roots, indicating that Al-treatment affected Pi transport more in *C. grandis* than in *C. sinensis* roots. To sum up, +Al *C. sinensis* seedlings had higher capacity to maintain the cellular P homeostasis *via* enhancing P acquisition and utilization, hence protecting plants against Al-toxicity. This agrees with our data that leaf, stem and root P level, and P uptake per plant were higher in +Al *C. sinensis* than in +Al *C. grandis* seedlings (**Figures 2C,D**; Yang L.T. et al., 2011).

### Genes Related to S Transport and Metabolism

S-mediated alleviation of Al-toxicity has been found in barley (Dawood et al., 2012) and wheat (Zhang et al., 2010). Here, we isolated one upregulated sulfate transporter (SULTR) 3,5 isoform 1 gene and two downregulated genes encoding SULTR 1,3 isoform 1 and SULTR 3.1-like, two upregulated sulfate/bicarbonate/oxalate exchanger and transporter sat-1 genes, and two upregulated sulfite exporter TauE/SafE family protein genes from +Al *C. grandis* roots, but only two upregulated genes encoding SULTR 3,5 isoform 1 and SULTR 3.1-like and one downregulated *sulfite exporter TauE/SafE family protein* from +Al *C. sinensis* roots (Supplementary Table S7). Thus, S uptake and export might be increased and reduced in +Al *C. sinensis* seedlings, respectively, thus enhancing S level and the Al-tolerance of *C. sinensis*. This was also supported by our data that S level was higher in *C. sinensis* roots and leaves than in *C. grandis* ones at each given Al level except for a similar root S level between the two species at the absence of Al (**Figures 2E,F**). S metabolism is the kernel pathway for the biosynthesis of molecules necessary for plant growth and development as well as tolerance to environmental stresses including Al (Yang et al., 2007; Anjum et al., 2015; Jiang et al., 2015). Here, we identified more downregulated (25) than upregulated (13) genes from +Al *C. grandis* roots, but less downregulated (9) than upregulated (14) genes related to S metabolism from +Al *C. sinensis* roots (Supplementary Table S7). Based on these results, we concluded that genes involved in S transport and metabolism might contribute to the Al-tolerance of *C. sinensis* by elevating cell S level and increasing the biosynthesis of S-containing compounds responsible for Al detoxification.

### Genes Related to Antioxidation

Al can elicit the production of ROS in plant cells (Yin et al., 2010; Li et al., 2016). Plants have developed diverse mechanisms for the detoxification of ROS. Besides the upregulation of some S metabolism-related genes, other genes related to antioxidation might be altered in +Al citrus roots. Here, we isolated 17 upregulated antioxidant enzyme genes (i.e., 11 *PODs*, five *GLPs* and one *peroxiredoxin-2B-like*) and three downregulated *GLP 9-3* from +Al *C. sinensis* roots. By contrast, we obtained 14 upregulated (i.e., five *PODs* and nine *GLPs*) and eight downregulated (i.e., four *PODs* and four *GLP 9-3*) genes from +Al *C. grandis* roots (Supplementary Table S8). Generally viewed, the expression of antioxidant enzyme genes was upregulated in +Al *C. sinensis* and *C. grandis* roots, especially in +Al *C. sinensis* roots. This agrees with our report that the levels of proteins related to ROS scavenging were increased in +Al *C. sinensis* roots, but were less altered in +Al *C. grandis* roots (Jiang et al., 2015).

### Genes Related to Polysaccharide, Cell Wall and Cytoskeleton Metabolism

Root cell wall is the key target for Al-toxicity and Al-tolerance (Horst et al., 2010). Here, we isolated 90 downregulated and 28 upregulated, and 25 downregulated and 39 upregulated genes related to polysaccharide and cell wall metabolism from +Al *C. grandis* and *C. sinensis* roots, respectively (Supplementary Table S9), implying that polysaccharide and cell wall metabolism was less impaired in +Al *C. sinensis* than in +Al *C. grandis* roots.

The Al-induced biosynthesis of root callose is an indicator of Al-injury and a reliable index for the evaluation of Al-tolerance (Horst et al., 2010). The upregulation of *callose synthase 5-like* in +Al *C. grandis* roots also support the above inference that *C. grandis* had less Al-tolerance than *C. sinensis*. In addition, we isolated six downregulated and one upregulared *cellulose synthases* from +Al *C. grandis* roots, but only one downregulated and one upegulated *cellulose synthases* from +Al *C. sinensis* roots. This agrees with the report that the Al-induced decrease in cellulose synthesis was less severe in Al-tolerant than in Al-sensitive wheat cultivar. The reduction in cellulose synthesis has been suggested to be the cause responsible for the rapid inhibition of root elongation under Al-stress (Teraoka et al., 2002). The Al-induced downregulation of *cellulose synthases* also agrees with our result that *callose synthase 5-like* was upregulated in +Al *C. grandis* roots, because the Al-induced inhibition of cellulose synthesis has been suggested to be in favor of callose synthesis (Teraoka et al., 2002).

The interaction of Al with cell wall pectin may cause the stiffening of cell walls, thus inhibiting root cell elongation (Tabuchi and Matsumoto, 2001). Pectinesterases (PEs) catalyze the deesterification and deacetylation of pectin, and potentially regulate the degradation of cell wall *via* modifing the accessibility of pectin to the action of cell wall degrading enzymes (Chandran et al., 2008). Thus, PEs may improve cell wall loosening, thereby compensating for the Al-induced cell wall stiffening. Here, we obtained 16 downregulated genes involved in pectin deesterification (Cs1g16550, Cs1g16560, Cs5g33450, Cs3g06900, Cs4g06650, Cs4g06630, Cs4g06670, Cs4g06690 and orange1.1t02719), degradation (Cs8g11330, orange1.1t01738 and Cs7g21940), biosynthesis (Cs2g01430, Cs5g33680 and Cs3g24380) and depolymerization (Cs2g10560) and three upregulated genes (Cs2g16380, Cs7g16400 and orange1.1t00214) involved in pectin biosynthesis from +Al *C. grandis* roots, but all the four DEGs (orange1.1t00214, orange1.1t01727, Cs3g06900 and Cs5g33420) related to pectin deesterification were upregulated in +Al *C. sinensis* roots. Thus, the elevated mRNA levels of four genes might contribute to the higher Al-tolerance of *C. sinensis*.

The modifications of the cellulose-xyloglucan network (primary cell walls) are required for plant cell expansion (Yang J.L. et al., 2011). The modifications of primary cell walls are catalyzed by various enzymes, including xyloglucan endotransglucosylase/hydrolase (XTH) family protein. XTHs catalyze either endotransglycosylation of xyloglucan *via* xyloglucan endotransglucosylase (XET) activity and/or the hydrolysis of xyloglucan *via* xyloglucan endohydrolase (XEH) activity, thereby contributing to cell wall loosing (Chandran et al., 2008). Yang J.L. et al. (2011) showed that the Al-induced inhibition of XET activity was the key process causing the inhibition of root growth under Al-stress. Here, we identified nine downregulated *XTH* (*XET*) and three downregulated genes encoding xyloglucanspecific endoglucanase inhibitor protein from +Al *C. grandis* roots, but only one upregulated *xyloglucan-specific endoglucanase inhibitor protein* from +Al *C. sinensis* roots, suggesting that the downregulation of *XTH* (*XET*) played a role in the Al-induced inhibition of *C. grandis* roots. This agrees with our data that Al-treatment only reduced *C. grandis* root DW (**Figure 1A**), and that *callose synthase 5-like* was upregulated only in +Al *C. grandis* roots because the Al-induced inhibition of XET activity and the deposition of callose in *Arabidopsis* roots was paralleled (Yang J.L. et al., 2011).

Expansins, which serve as wall-loosening proteins, play a role in plant growth and response to abiotic stresses (Cosgrove, 2015). As shown in Supplementary Table S9, we isolated five downregulated *expansins* from +Al *C. grandis* roots, but four upregulated and five downregulated *expansins* from +Al *C. sinensis* roots. Also, we isolated two downregulated *extensins* involved in root hair morphogenesis and elongation (Baumberger et al., 2001) from +Al *C. grandis* roots. Thus, the downregulation of *expansins* and *extensins* might play a role in the Al-induced inhibition of root growth and Al-sensitivity in *C. grandis* seedlings. However, recent work showed that an Al-inducible expansin gene, *OsEXPA10* was necessary for normal root growth, but its contribution to the high Al-tolerance of rice was small (Che et al., 2016).

Ascorbate oxidase (AO) has been suggested to increase cell elongation *via* producing monodehydroascorbate and dehydroascorbate (Pignocchi et al., 2003). Our results showed that *L-ascorbate oxidase homolog* was inhibited and induced in +Al *C. grandis* and *C. sinensis* roots, respectively, which agrees with our data that root growth inhibition only occurred in +Al *C. grandis* (**Figure 1A**).

In addition to scavenging H_2_O_2_, PODs also produce H_2_O_2_ at the expense of NAD(P)H. The total activities of PODs increase when exposed to Al (Cakmak and Horst, 1991). The exact roles of the Al-induced increase in PODs-mediated H_2_O_2_ production in roots are unclear (Maron et al., 2008). PODs play a role in the oxidative cross-linking of cell wall compoments, thus increasing cell wall stiffening and decreasing cell wall extensibility, which has been associated with the Al-induced inhibiton of root growth (Ma et al., 2012). POD-mediated oxidative cross-linking, however, has been demonstrated to be a potential mechanism of Al-tolerance (Ma et al., 2004; Maron et al., 2008). Plants may use oxalate oxidase (OXO)- and/or POD-dependent production of H_2_O_2_ to restructure the cell walls and restrict Al entry *via* decreasing cell wall porosity (Delisle et al., 2001; Maron et al., 2008). Tamás et al. (2005) showed that the Al-induced cell death of barley-root border cells due to the increase in POD- and OXO-mediated production of H_2_O_2_ played a role in protecting root tips against Al-toxicity *via* chelating Al in the dead cells. Here, we identified five upregulated and four downregulated *PODs* from +Al *C. grandis* roots, but 11 upregulated *PODs* from +Al *C. sinensis* roots, indicating that PODs might be involved in the Al-tolerance of *C. sinensis*. Similarly, the expression levels of all genes encoding the germin-like proteins (GLPs), the H_2_O_2_ generating enzymes with OXO and superoxide dismutase (SOD) activities, were increased in +Al *C. sinensis* and *C. grandis* roots except for three and four downregulated *GLP 9-3* in +Al *C. sinensis* and *C. grandis* roots, respectively. Maron et al. (2008) observed that the expression levels of *OXOs* were constitutively higher and were increased by Al only in Al-tolerant maize roots. Thus, GLPs might play a role in the higher Al-tolerance of *C. sinensis*.

Cytoskeleton has been demonstrated to be a target of Al-toxicity in plants. In the early response to Al, a close relationship between the root growth inhibition and the impairments of the integrity of cytoskeletal elements (microtubules and actin microfilaments) was observed, specifically in the distal part of the transition zone in the Al-sensitive maize (Sivaguru et al., 1999). Here, we isolated four downregulated (Cs5g03620, Cs8g03680, Cs4g12990, and Cs8g09980) and two upregulated (Cs8g09980 and Cs1g15130) genes related to cytoskeleton metabolism from +Al *C. grandis* roots, indicating that cytoskeleton might be impaired in roots, thereby inhibiting root growth. By contrast, we isolated 11 upregulated and one dowregulated cytoskeleton metabolism-related genes from +Al *C. sinensis* roots.

These results strongly suggested that Al-treatment impaired polysaccharide, cell wall and cytoskeleton metabolism in *C. grandis* roots, thus inhibiting root growth and lowering Al-tolerance.

### Genes Related to Carbohydrate and Energy Metabolsim

Energy shortage is a common phenomenon for stressed plants. A close relation exists between stress tolerance and energy availability (Baena-González and Sheen, 2008). As shown in Supplementary Table S10, we isolated 6 downregulated (Cs2g03260, Cs8g06950, orange1.1t05835, Cs3g22480, Cs2g13410 and Cs5g30520) and two upregulated (Cs4g08150 and Cs4g01490) genes related to aerobic glycolysis from +Al *C. grandis* roots, but only six upregulated genes (Cs5g30520, Cs4g01490, Cs1g15970, Cs4g13070, Cs3g18540 and Cs6g08840) from +Al *C. sinensis* roots. Thus, glycolysis might be repressed in +Al *C. grandis* roots, but induced in +Al *C. sinensis* roots. Wang et al. (2014) found that the majority of the Al-responsive proteins related to glycolysis were upregulated in Al-tolerant rice cultivar. Thus, the higher glycolysis in +Al *C. sinensis* roots might contribute to the Al-tolerance of *C. sinensis* by keeping basic root respiration and meeting the increased requirement for engery (Wang et al., 2014). Interestingly, the mRNA level of phosphoenolpyruvate carboxykinase (PEPCK) [ATP]-like (a key regulatory enzyme in gluconeogenesis) gene was repressed in +Al *C. sinensis* roots. This agrees with the report that PEPCK was severely reduced in Al-stressed *Pseudomonas fluorescens* cells (Lemire et al., 2008). PEPCK plays a role in the catabolism of malate/citrate during fruit ripening (Baldicchi et al., 2015). Malate level was enhanced in the ripening fruits of *PEPCK*-RNAi tomato lines relative to the wild type (Huang et al., 2015). Thus, the down-regulation of *PEPCK [ATP]-like* might promote malate/citrate accumulation in +Al *C. sinensis* roots. By contrast, the expression of *NADP-malic enzyme 4* (*NADP-ME4*) was upregulated in +Al *C. sinensis* roots. Overexpression of an Al-induced ME gene (*SgME1*) from *Stylosanthes* increased malate synthesis and prevented yeast, *Arabidopsis* and bean hairy roots from Al-toxicity through increased root malate synthesis and/or accompanied root malate exudation (Sun et al., 2014). Thus, root levels or secretion of malate/citrate might be enhanced due to decreased expression of *PEPCK* and increased expression of *NADP-ME4*, hencing conferring Al-tolerance of *C. sinensis*. This agrees with our report that +Al *C. sinensis* seedlings had higher or similar root levels of malate, citrate and malate + citrate and increased root secretion of malate and citrate than +Al *C. grandis* ones (**Figure 3**; Yang L.T. et al., 2011), suggesting that *C. sinensis* roots might have a more efficient chelation system, thus protecting roots against Al-toxicity. In addition, the Al-induced alterations of other genes related to energy and carbohydrate metabolism differed between the two citrus species. For example, we isolated two upregulated *alcohol dehydrogenase* (*ADH*) and one downregulated *ADH class-3* (*ADH-3*) related to anaerobic glycolysis from +Al *C. sinensis* roots. This agrees with the reports that ADH activity was greatly increased in Al-treated wheat roots (Copeland and De Lima, 1992). Study showed that STOP1 regulated multiple gene expression, thus protecting plants against H^+^ and Al toxicities. Genes involved in pH-regulating metabolism such as *ME, pyruvate decarbohylase (PDC)* and *ADH* [or *lactate dehydrogenase* (*LDH*)] were downregulated in *stop1* mutant (Sawaki et al., 2009). Thus, the Al-induced upregulation of *ADH*, *NADP-ME4* and *thiamine pyrophosphate dependent pyruvate decarboxylase family protein* might contribute to the Al-tolerance of *C. sinensis via* keeping the pH homeostasis of cells. In short, we obtained more upregulated (18) than downregulated (10) genes related to energy and carbohydrate metabolism from +Al *C. sinensis* roots, but more downregulated (19) than upregulated (9) genes from +Al *C. grandis* roots. The energy and carbohydrate metabolism displayed higher adaptive responses to Al in *C. sinensis* than in *C. grandis* roots (Jiang et al., 2015).

### Genes Related to Protein and Amino Acid Metabolism

We isolated 88 and 56 Al-responsive genes related to protein metabolism from *C. grandis* and *C. sinenis* roots, respectively (Supplementary Table S11), which might be related to the higher Al-tolerance of *C. sinensis*. This disagrees with our reports that more Al-responsive proteins related to protein metabolism were identified in *C. sineneis* than in *C. grandis* roots (Jiang et al., 2015). Most of these genes were involved in protein degradation, followed by protein ubiquitination-related genes, heat shock protein (HSP)/chaperone genes and protein biosynthesis-related genes. However, our previous study showed that over 50% of Al-responsive genes related to protein metabolism were involved in protein biosynthesis in *C. sinensis* and *C. grandis* roots. The differences between the Al-responsive proteins obtained by iTRAQ (Jiang et al., 2015) and the DEGs revealed by RNA-Seq might be due to PTMs.

Protein degradation can not only supply respiratory substrates for stressed plants, but also trigger adaptive responses to stressed conditions by nutrient recycling (Araújo et al., 2011). Proteases (also called peptidases and proteinases) play crucial roles in the selective degradation of specific proteins and the strict control of protein quality in plants when exposed to stressed conditions. The ubiquitin proteosome pathway may degradate futile and inactive proteins in a more specific manner relative to proteases (Kumari et al., 2008). Here, we identified 45 downregulated and 26 upregulated, and 28 downregulated and 18 upregulated genes related to protein degradation in +Al *C. grandis* and *C. sinensis* roots, respectively. The more downregulation of protein degradation-related genes might be responsible for the less Al-tolerance of *C. grandis*.

Amino acids play important roles in plant responses to different stresses. Here, we isolated eight upregulated and four downregulated, and 21 downregulated and four upregulated amino acid metabolism-related genes from +Al *C. sinensis* and *C. grandis* roots, respectively (Supplementary Table S11), demonstrating that amino acid metabolism might be involved in the higher Al-tolerance of *C. sinensis*.

### Genes Related to Lipid Metabolism

The Al-induced alterations of the total and the relative abundance of lipids (particularly phospholipids) as well as the fatty acid composition and the degree of fatty acid unsaturation differed between Al-tolerant and Al-sensitive cultivars (Peixoto et al., 1999; Huynh et al., 2012). Huynh et al. (2012) reported that phospholipids and MGDG in roots decreased in Al-sensitive rice cultivars when exposed to Al, while the amount of lipid classes kept stable in Al-tolerant ones. Here, we isolated 39 downregulated and 19 upregulated, and 12 downregulated and 28 upregulated genes related to metabolisms of lipids and fatty acids from +Al *C. grandis and C. sinensis* roots, respectively (Supplementary Table S12), demonstrating that lipid metabolism was less affected by Al in *C. sinensis* roots, and that lipid metabolism might be upregulated and downregulated in +Al *C. sinensis* and *C. grandis* roots, respectively. This might be involved in the Al-tolerance of *C. sinensis*.

All the four DEGs related to jasmonic acid (JA) biosynthesis were upregulated in +Al *C. sinensis* roots, but only two upregulated lipoxygenase (LOX) and two downregulated [*LOX3* and *allene oxide synthase* (AOS)] genes were isolated from +Al *C. grandis* roots (Supplementary Table S12). This agrees with the report that both JA biosynthesis and level might be elevated in +Al *C. sinensis* roots (Jiang et al., 2015). Peixoto et al. (2001) reported that both LOX activity and Al-induced increase in LOX activity were greater in Al-tolerant than in Al-sensitive sorghum cultivar. Therefore, JA metabolism might be upregulated in +Al *C. sinensis* roots, thereby contributing to the higher Al-tolerance of *C. sinensis*.

GDSL esterases/lipases play key role in plant abiotic stresses. Overexpression of *LTL1* encoding a GDSL-motif lipase conferred the tolerance of salt and other environmental stresses in transgenic yeast and *Arabidopsis* plants (Jiang et al., 2012; Naranjo et al., 2006). Here, we identified four upregulated and two downregulated, and four downregulated *GDSL lipases* in +Al *C. sinensis* and *C. grandis* roots, respectively, demonstrating the possible involvement of *GDSL lipases* in citrus Al-tolerance.

We isolated 11 downregulated genes (Cs4g04540, Cs6g22090, orange1.1t02015, Cs2g06240, Cs3g15530, Cs3g20840, Cs4g17260, Cs5g01990, Cs6g08600, orange1.1t02024 and Cs4g06470) from +Al *C. grandis* roots, and four upregulated (Cs3g20840, orange1.1t00556, Cs8g20310 and Cs4g06430) and one downregulated (Cs4g06470) genes involved in fatty acid biosynthesis from +Al *C. sinensis* roots (Supplementary Table S12). Thus, fatty acid biosynthesis might be downregulated and upregulated in +Al *C. grandis* and *C. sinensis* roots, respectively. This agrees with the report that the levels of linolenic and palmitic acids, which comprised together about 65–71% of total fatty acids in the root plasma membrane fraction, increased in the Al-tolerant sorghum cultivar roots but decreased in the Al-sensitive ones (Peixoto et al., 2001).

Zhang et al. (2016) demonstrated that overexpression of *OsMGD* conferred Al-tolerance in transgenic tobacco plants *via* the regulation of galactolipid biosynthesis. However, the different Al-tolerance between the two citrus species could not be explained in this way because the Al-induced upregulation of *MGD2* was greater in *C. grandis* than in *C. sinensis* roots.

### Genes Related to Signal Transduction

As shown in Supplementary Table S13, 104 downregulated and 70 upregulated, and 55 downregulated and 43 upregulated genes related to signal transduction were isolated from +Al *C. grandis* and *C. sinensis* roots, respectively. Over two-thirds of these DEGs belonged to protein kinases, followed by DEGs in Ca/calmodulim- and hormone-medicated signal transduction, and protein dephosphorylation. For the optimal regulation, a proper balance must be struck between protein kinases and phosphatase in any given plant cell. Jiang et al. (2015) demonstrated that Al-tolerant *C. sinensis* roots could keep a better balance between phosphorylation and dephosphorylation than *C. grandis* roots when exposed to Al. Here, we isolated 76 downregulated and 54 upregulated genes related to protein phosphorylation, and five downregulated and three upregulated genes related to dephosphorylation from +Al *C. grandis* roots, but only 36 downregulated and 29 upregulated genes related to protein phosphorylation, and two upregulated and two downregulated genes related to dephosphorylation from +Al *C. sinensis* roots. Root protein phosphorylation/dephosphorylation was less affected in +Al *C. sineneis* roots. This might be associated with the Al-tolerance of *C. sinensis*. However, the exact roles of these kinases and phosphatases require further investigation. For example, Sivaguru et al. (2003) showed that overexpression of an Al-inducible *wall-associated receptor kinase 1* (*WAK1*) conferred Al-tolerance in transgenic *Arabidopsis*, suggesting that *WAK1* was one of the key candidates for Al-tolerance. Here, we identified seven upregulated and three downregulated *WAKs* in +Al *C. grandis* roots, but only four downregulated *WAKs* in +Al *C. sinensis* roots. Therefore, the difference in the Al-tolerance between the two citrus species can not be explained in this way.

Disruption of cytoplasmic Ca homeostasis has been proposed to be a primary trigger of Al-toxicity (Rengel and Zhang, 2003). Okekeogbu et al. (2014) found that the abundances of several Ca-binding proteins were elevated in the Al-treated tomato radicles, suggesting that Ca-binding proteins might play a role in tomato Al-tolerance. Here, we identified seven upregulated and two downregulated genes involved in Ca/calmodulim-medicated signal transduction in +Al *C. sinensis* roots, demonstrating that Al might trigger Ca/calmodulim-medicated signal pathways in these roots, thereby contributing to the Al-tolerance of *C. sinensis*. By contrast, we obtained 12 downregulated and four upregulated genes related to Ca/calmodulim-medicated signal transduction from +Al *C. grandis* roots, indicating that Al might impair Ca/calmodulim-medicated signal pathways in these roots, thus lowering the Al-tolerance of *C. grandis*.

Plant hormones, which play crucial roles in signal transduction, are involved in Al-tolerance. As shown in Supplementary Table S13, all the 10 DEGs related to hormone-mediated signal transduction were upregulated in +Al *C. sinensis* roots, indicating that Al might activate hormone-mediated signal pathways, thus enhancing *C. sinensis* Al-tolerance. By contrast, we isolated six downregulated and three upregulated genes from +Al *C. grandis* roots, implying that hormone-mediated signal pathways might be disturbed in these roots, thus affecting the Al-tolerance of *C. grandis*.

### Transcription Factors

As shown in Supplementary Table S14, 51 downregulated and 44 upregulated, and 37 downregulated and 33 upregulated *TFs* were identified in +Al *C. grandis* and *C. sinensis* roots, respectively. Most of them belonged to myeloblastosis (MYB), Apetala2 (AP2)/ethylene-responsive transcription factor (ERF), zinc finger [C-x8-C-x5-C-x3-H (CCCH) and Cys2/His2 (C2H2)], WRKY, homeobox, helix-loop-helix (bHLH) and NAC families. Similar results have been obtained on Al-treated soybean (You et al., 2011) and *Arabidopsis* (Kumari et al., 2008) roots.

STOP1, a C2H2-zinc-finger protein, played a key role in H^+^- and Al-tolerance (Sawaki et al., 2009). However, this could not explain the difference in the Al-tolerance between the two citrus species, because the Al-induced upregulation of two protein SENSITIVE TO PROTON RHIZOTOXICITY 1-like genes was greater in *C. grandis* than in *C. sinensis* roots. Also, ART1, a C2H2-type zinc-finger TF, has been demonstrated to regulate the expression of multiple Al-tolerance genes required for rice Al-tolerance (Yamaji et al., 2009). Here, we isolated one downregulated *C2H2-type zinc finger protein* and one upregulated *C2H2-like zinc finger protein* from Al-treated *C. grandis* and *C. sinensis* roots, respectively, implying that C2H2 zinc finger TFs might be involved in the higher Al-tolerance of *C. sinensis*.

Arenhart et al. (2014) found that the expression level of *ABA stress ripening 5* (*ASR5*) was not altered in the Al-sensitive rice cultivar roots when exposed to Al, but was upregulated in the Al-tolerant ones. Further analysis demonstrated that *ASR5* played a key role in Al-tolerance by mediating Al-responsive gene expression. Here, we identified three upregulated genes encoding ASR-related protein from +Al *C. grandis* roots, but only one downregulated ASR-related genes from +Al *C. sinensis* roots. Roselló et al. (2015) indicated that that Al increased *ASR1* expression level up to six fold in the roots of Al-sensitive rice cultivar, but not in Al-tolerant cultivar. Further studies are needed to elucidate the roles of ASR genes in plant Al-tolerance.

Plant WRKY TFs have been shown to play important roles in the responses to abiotic stresses including Al-toxicity (Chen L. et al., 2012). Mattiello et al. (2010) found that a member of the WRKY transcriptional family was upregulated only in Al-tolerant maize line roots when grown in acidic soil with elevated Al level. Here, we isolated two upregulated and four downregulated *WRKYs* from +Al *C. grandis* roots, and four upregulated and two downregulated *WRKYs* from +Al *C. sinensis* roots. The Al-induced upregulation of *WRKYs* might play a role in the Al-tolerance of *C. sinensis*. However, Ding et al. (2013) showed that *WRKY46* was repressed by Al and negatively regulated the expression of *ALMT1*. Mutation of *WRKY46* led to increased Al-tolerance by enhancing Al-induced secretion of malate and decreasing Al accumulation in root apices.

MYB TFs, especially the large family of plant-specific R2R3-MYB genes play a role in plant responses to environmental stresses. Dai et al. (2012) observed that the expression of *OsMYB2P-1*, an R2R3 MYB TF, was induced by P-deficiency, mainly in rice roots and stems, and that transgenic *Arabidopsis* and rice plants overexpressing *OsMYB2P-1* had enhanced tolerance to P-deficiency, while *OsMYB2P-1* RNAi transgenic rice plants were more sensitive to P-deficiency relative to wild-type plants. Here, we identified seven upregulated and three downregulated *MYBs* in +Al *C. sinensis* roots, and nine upregulated and 11 downregulated *MYBs* in +Al *C. grandis* roots. It is worth mentioning that the expression of *R2R3-MYB TF* and *R2R3 TF MYB108-like protein 1* was upregulated in +Al *C. sinensis* roots, while the expression of *R2R3 TF MYB108-like protein 1* and *R2R3-MYB TF* was downregulated in +Al *C. grandis* roots. Thus, the Al-induced upregulation of *MYB TFs* might contribute to the Al-tolerance of *C. sinensis via* enhancing the tolerance to P-starvation.

To conclude, the great difference in the Al-induced alterations of *TF* expression profiles in the two citrus species implies that TFs might play a role in the Al-tolerance of *C. sinensis*.

## Conclusion

**FIGURE 6:**
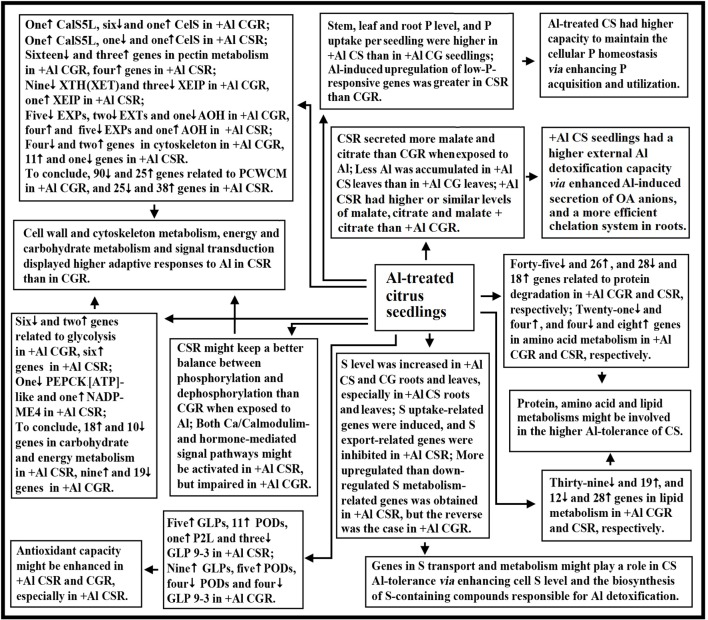
**A potential responses of citrus seedlings to Al.** AOH, ascorbate oxidase homolog; CG, *C. grandis*; CGR, *C. grandis* roots; CS, *C. sinensis*; CSR, *C. sinensis* roots; EXPs, expansins; EXTs, extensins; PCWCM, polysaccharide, cell wall and cytoskeleton metabolism; XEIP, xyloglucan-specific endoglucanase inhibitor protein.

Using RNA-seq, we isolated 1905 and 2670 Al-responsive genes from *C. sinensis* and *C. grandis* roots, respectively. Among these DEGs, only 649 DEGs were shared by the both. Through the integration of the present findings and the available data in the previous reports, a model for the adaptive responses of citrus seedlings to Al was proposed (**Figure 6**). There were common and unique mechanisms for Al-tolerance in citrus plants. The following several aspects might account for the higher Al-tolerance of *C. sinensis*: (a) +Al *C. sinensis* seedlings displayed a more efficient exclusion mechanism, a higher antioxidant capacity and a more efficient chelation system in roots; (b) +Al *C. sinensis* seedlings had higher capacity to maintain the cellular P homeostasis *via* improving P acquisition and utilization; (c) Genes in S transport and metabolism might contribute the Al-tolerance of *C. sinensis via* enhancing cell S level and the biosynthesis of S-containing compounds responsible for Al detoxification; (d) Al impaired polysaccharide, cell wall and cytoskeleton metabolism in *C. grandis* roots, thus lowering Al-tolerance; (e) Energy and carbohydrate metabolism, and signal transduction displayed higher adaptive responses to Al in *C. sinensis* than in *C. grandis* roots; (f) Genes involved in fatty acid, protein and amino acid metabolisms might be involved in the Al-tolerance of *C. sinensis*. To conclude, we first comparatively investigated the transcriptomic responses of citrus roots to Al using two citrus species with different Al-tolerance.

## Data Access

RNAseq are submitted to Gene Expression Omnibus (GEO) under accession no GSE85958 (http://www.ncbi.nlm.nih.gov/geo/query/acc.cgi?acc = GSE85958).

## Author Contributions

PG carried out most of the experiments and drafted the manuscript. Y-PQ participated in the design of the study. L-TY participated in the design and coordination of the study. N-WL participated in the analysis of data. XY participated in the analysis of Al and P. YY participated in the cultivation of seedlings. L-SC designed and directed the study and revised the manuscript. All authors have read and approved the final manuscript.

## Conflict of Interest Statement

The authors declare that the research was conducted in the absence of any commercial or financial relationships that could be construed as a potential conflict of interest.

## Footnotes

1 http://www.phytozome.net/search.php?method=Org_Csinensis

2 http://www.uniprot.org/

3 http://www.kegg.jp/

4 http://www.geneontology.org/
