# Supplementary material for: Root Adaptive Responses to Aluminum-Treatment Revealed by RNA-Seq in Two Citrus Species With Different Aluminum-Tolerance
Source: Front Plant Sci. 2017 Mar 8;8:330. doi: 10.3389/fpls.2017.00330 (PMC5340773; doi:10.3389/fpls.2017.00330)
Supplement: Supplementary file 1 [file Table_1.DOC]

**TABLE S1| Specific primer pairs used for qRT-PCR expression analysis.**

| **Gene ID** | **Forward primer** | **Reverse primer** |
| --- | --- | --- |
| *C. sinensis* |  |  |
| Cs9g07780 | TGCTTGAGGATGGTGTT | TGAATGGAAGGATGTTTAG |
| Cs3g14760 | ACAGTAAGAAAAGGCTCC | CAAATGCGATAATCAACA |
| Cs3g20800 | CTTTACTTCCCTTTGCC | ATGTTGCTTTCTTCTGATT |
| Cs8g01840 | GAGGCTCAGCACAGAA | TCAAGCAAGTCATCAAAC |
| Cs1g13970 | GCGACAGCGGTCTAAA | GGCTCTTCGTTGGGTT |
| Cs2g06650 | ATGGCATTAGCAAGGTT | CAAGGGAAGGTGAGAAA |
| Cs5g08370 | AGTGGAGCGAAGAAGAT | TGTTGAACCCGAATAAA |
| Cs2g02080 | TCTGCTCAATGGGTGTC | CCCTCTGCCAAAGTCGT |
| Cs2g29820 | CCAACACCACGAGAAGT | CAGCCTCAATAAACAAGAC |
| Cs3g10920 | GGGGCACCTTATCTCAG | GCATCCAATCACCATCTT |
| Cs4g02000 | CTGGCTTGCGAAGAGTG | ATTCATCGTCGTTAGGG |
| Cs4g04460 | AGAGTTTGCTGGAGTGTT | CTTCAATGGTGATGCTTA |
| Cs4g05660 | CACTTGCCACTTCTGTTG | ACCTGTCCTTCACCCAC |
| Cs4g17050 | ACATTAGAAGGCAGCAG | CCACGGGTAGTAAAACA |
| Cs4g19200 | GCACAACCTTCGCCAATA | CGCCTGAAAATGCTCCC |
| Cs5g03070 | AGCAGTTTCCAGCATTT | TTGTTCATTGGGGTTCT |
| Cs5g06930 | GGAACCGTATCAAGTATGC | CGACAGGACCCGTAATC |
| Cs5g09050 | TTCATTGCTGACACCTT | TCTGATTCCGCTCTTG |
| Cs5g29860 | GGTGATAAGTTGGGTAGAA | CAGAAACGAAAGAAGCAG |
| Cs5g30300 | ATGCGGCGAGTTCAGA | CCCCAGGGTGGTTGTC |
| Cs6g09960 | CTGCTGTAGCGGGATT | GGCTGATTTGGTAAGGA |
| Cs6g12750 | GCCATCACTTTGGTCTC | CCTATTCAATCCTTTTCCTA |
| Cs6g21460 | ATTGGTCTCATCCGTTCA | GGAGGGCAAAAGTTAGTG |
| Cs7g07000 | GAAAACAAAGGCACTCG | TCTTATCAAAGCCAAAACT |
| Cs8g20150 | CCGTTCCTCTGGCTGTT | GCTCCGTCGCATTACC |
| Cs9g02910 | TCAGCCTATTTTATGGTT | GAGTTCAGGATTGGGAC |
| orange1.1t01729 | CCTCCTGAACTGGTGC | TTGGGTTTGGTTATCG |
| orange1.1t02668 | ATCCTCCCACTTCTCAA | TCCTGCTCCACTCGTA |
| orange1.1t03629 | GGGTGATTTGGGCTCT | CAAGGGATTCTGTGGC |
| orange1.1t05568 | AGGACAAGGAAGGGATT | GACGAAGAACCAAGTGC |
|  |  |  |
| *C. grandis* |  |  |
| Cs3g19060 | GGTCCCCAAGGCATCA | AGCCGCTCCCCAGTTA |
| Cs9g05680 | GATTTTGGCACCTTCTA | CTCCTATTTGTTCCTTTCT |
| Cs9g17990 | CAGGAAAGTCACCGAA | ATCCACAAGCACCTCTAT |
| Cs3g14760 | ACAGTAAGAAAAGGCTCC | CAAATGCGATAATCAACA |
| Cs2g03260 | GCTTCTACTTCGGCATC | CTCAGCAATAGGGGTGT |
| Cs8g06950 | GCAATCCGAAGTGAGA | TTTACCAAAGTTTTAGCAG |
| Cs7g05690 | GACGGATAGAGGCTGAG | TTACAACCATACGAGGC |
| Cs3g19250 | TTCTTGGCAATCATCTTC | CAAATCCCACCACTAAA |
| Cs7g02820 | TCTTTGCCGTCTACTCC | AATGTGCTCAGCCTTCT |
| Cs5g08370 | AGTGGAGCGAAGAAGAT | TGTTGAACCCGAATAAA |
| Cs1g05600 | ATCCAAGAAGCAGCAGT | GCAAATAAGCATAATCGTA |
| Cs1g15090 | TATCTGCTCCTAAAAGTCG | GAACAAATATGCCACCC |
| Cs1g16030 | GCCACAGACCAACTCA | TCACAGCATTCCCAAA |
| Cs1g17380 | TGCCGAATGTTGTTAGC | GGTGGGAAAGCCTGAA |
| Cs1g25930 | CTAAAGGAAGAAAAGATGG | TTTGGAAGGAGGAACAG |
| Cs3g13720 | TCTCGCTTTTCCTTGTC | AGCCATTTGGGTCTGT |
| Cs3g18650 | AATCAATCCCTCATCTGT | GCTTCCGTCAATCCTAT |
| Cs3g20770 | TCCATTGTCCGTTCTG | ACGCTTTTCTTTCTCCT |
| Cs3g21500 | AGCGGTGACTATGGCA | GCTCTGGGTCCTTTTCT |
| Cs3g21510 | GTGACTATGGCAGGAAGG | CTCGTGGAACGCAAAT |
| Cs4g01990 | TTCTTCACTTCATAAACGAG | AATAACAAGCCGAGCC |
| Cs4g12860 | TCTCCTTCATCGTCCTC | AATGTCCAATCCACCTC |
| Cs5g01190 | CTGTTTCTGTTTGTTTCGCTAT | CCGCCGTCACCTTTTC |
| Cs5g04280 | CACCTACCGCATCTACG | CCAGTTGGGTTGTCTCAT |
| Cs8g05320 | GGTAGACATAGCCAGAATC | GGGTAAATGGAGGGTT |
| Cs8g13540 | TCAAAAGCAAAGACACTG | TATCCAAACTCAAAGCAC |
| Cs9g04030 | CGACAACAGAGGCTACG | GGTCTTGGAGGAATAAACA |
| orange1.1t04027 | AGGGTAGTGCCAGAGTT | CGTTGGTAAGCATAGGA |
| orange1.1t04194 | TACGCAGGATTGGACC | GAAGAGTAATGCCGAGAC |
| orange1.1t06069 | AGAGTGGCAAAGCAGG | TATGAGCCGACGAGGA |
|  |  |  |
| JN580571 (*β-tublin*) | CCCTTTACGACATCTGTTTCCG | TGGCATCCCACATTTGCTG |
| JN191387 (*actin*) | AGAACTATGAACTGCCTGATGGC | GCTTGGAGCAAGTGCTGTGATT |
| GU362416 (*polyubiquitin*) | TCTTCGCAGGAAAGCAACTCGAG | CCTCAGACGCAAAACCAGATGAAG |
|  |  |  |
