# Supplementary material for: Root Adaptive Responses to Aluminum-Treatment Revealed by RNA-Seq in Two Citrus Species With Different Aluminum-Tolerance
Source: Front Plant Sci. 2017 Mar 8;8:330. doi: 10.3389/fpls.2017.00330 (PMC5340773; doi:10.3389/fpls.2017.00330)
Supplement: Supplementary file 5 [file Table_5.doc]

**TABLE S5 | DEGs involved in cellular transport in Al-treated *C. sinensis* and *C. grandis* roots.**

| **ID** | **Description** | ***C. grandis*** | |  | ***C. sinensis*** | |
| --- | --- | --- | --- | --- | --- | --- |
|  |  | **log2 (FC)** | **FDR** |  | **log2 (FC)** | **FDR** |
| **Cs5g31410** | **Aluminum-activated malate transporter 13-like** | **1.118850895** | **0.007858** |  | **-1.764418449** | **0.0111773** |
| **Cs6g12780** | **Aluminum activated malate transporter family protein** | **8.500301503** | **2.98E-80** |  | **6.577990223** | **4.17E-100** |
| **Cs6g12800** | **Aluminum-activated malate transporter 2** | **-1.744692344** | **0.000367** |  | **-1.153449736** | **0.040493** |
| Cs5g01700 | Aluminum-activated malate transporter 10 |  |  |  | -1.633550318 | 2.40E-30 |
| Cs6g12750 | Aluminum activated malate transporter family protein |  |  |  | 3.143334448 | 2.44E-07 |
| Cs9g16870 | Multidrug and toxin extrusion protein 2-like | -1.259931264 | 2.60E-5 |  |  |  |
| Cs3g16650 | Multidrug and toxin extrusion protein 1 |  |  |  | -1.087579569 | 6.98E-19 |
| Cs1g26350 | MATE efflux family protein |  |  |  | 1.459690009 | 2.77E-11 |
| orange1.1t03616 | MATE efflux family protein isoform 3 |  |  |  | 1.530236543 | 0.033841 |
| orange1.1t03623 | MATE efflux family protein isoform 1 |  |  |  | 1.213854868 | 0.000292 |
| **Cs7g01770** | **Ferric reductase defective 3b** | **4.460134012** | **1.91E-103** |  | **3.310928564** | **0** |
| **Cs6g09440** | **Iron regulated 1 protein** | **4.190615178** | **1.44E-76** |  | **2.487911975** | **8.42E-88** |
| XLOC_029822 | Metal transporter Nramp6 | -1.01700463 | 0.012270 |  |  |  |
| **Cs3g18230** | **Aquaporin, MIP family, NIP subfamily** | **-3.634548151** | **6.43E-47** |  | **-2.631230309** | **1.27E-79** |
| Cs6g11670 | Aquaporin 2 | -2.693394112 | 0.027782 |  |  |  |
| Cs8g17900 | Aquaporin TIP1-3-like | -2.577276216 | 9.65E-42 |  |  |  |
| Cs6g21290 | Aquaporin NIP2-1-like |  |  |  | -1.351883635 | 1.87E-50 |
| Cs9g06260 | Aquaporin, MIP family, NIP subfamily |  |  |  | 1.458482371 | 0.00049 |
| **Cs3g21080** | **ABC transporter G family member 22-like** | **-5.284167499** | **3.63E-8** |  | **-10.26274321** | **0.005296** |
| **Cs4g04420** | **ABC transporter I family member 17** | **2.297835532** | **3.31E-34** |  | **1.84324521** | **2.14E-166** |
| Cs6g20280 | ABC transporter family protein isoform 1 |  |  |  | -1.374462747 | 3.69E-79 |
| **Cs1g16850** | **Multidrug/pheromone exporter, MDR family, ABC transporter family** | **-1.803868142** | **1.25E-14** |  | **-1.590981082** | **5.75E-17** |
| Cs4g15420 | Multidrug/pheromone exporter, MDR family, ABC transporter family |  |  |  | 2.116598009 | 7.29E-51 |
| Cs2g30650 | ABC-2 type transporter family protein isoform 2 | 4.32064844 | 3.39E-9 |  |  |  |
| Cs5g18680 | ABC-2 type transporter family protein |  |  |  | 1.329518105 | 4.85E-05 |
| Cs1g15320 | Major facilitator superfamily protein | -1.147635465 | 0.000362 |  |  |  |
| Cs4g17460 | Major facilitator superfamily protein isoform 2 | -1.876764685 | 6.44E-5 |  |  |  |
| Cs4g19660 | Major facilitator superfamily protein | 1.790866883 | 4.61E-6 |  |  |  |
| **Cs5g32060** | **Major facilitator superfamily protein isoform 1** | **-1.733681314** | **2.66E-9** |  | **1.375570535** | **9.46E-34** |
| **Cs7g20780** | **Major facilitator superfamily protein isoform 1** | **-1.28201297** | **1.32E-8** |  | **-1.306272056** | **1.07E-24** |
| Cs7g22000 | Major facilitator protein | 1.05821306 | 7.87E-5 |  |  |  |
| Cs8g02840 | Major facilitator superfamily protein | -1.426451507 | 3.56E-14 |  |  |  |
| Cs8g04000 | Major facilitator superfamily protein | -1.287265224 | 0.000120 |  |  |  |
| Cs8g15700 | Major facilitator superfamily protein | -1.358172191 | 6.59E-10 |  |  |  |
| Cs1g23890 | Major facilitator superfamily protein |  |  |  | -1.163708231 | 0.027952 |
| Cs1g25570 | Major facilitator superfamily protein |  |  |  | 2.025966549 | 4.63E-43 |
| Cs4g04910 | MFS |  |  |  | -1.051456923 | 1.21E-18 |
| Cs7g05210 | Major facilitator superfamily protein isoform 2 |  |  |  | -1.478838837 | 0.024603 |
| Cs8g18970 | Major facilitator superfamily protein isoform 1 |  |  |  | -1.571253487 | 8.32E-26 |
| Cs9g06620 | Major facilitator superfamily protein |  |  |  | 1.175201286 | 8.50E-100 |
| orange1.1t02578 | Major facilitator protein |  |  |  | -1.26004931 | 5.61E-07 |
| Cs5g30300 | Magnesium transporter MRS2-2 isoform 2 |  |  |  | -1.696751637 | 0.009998 |
| Cs2g11370 | Potassium transporter 11-like | 109.69965 | 1.81E-14 |  |  |  |
| Cs2g11380 | Potassium transporter 11-like | 8.72688 | 4.68E-14 |  |  |  |
| Cs7g02320 | Potassium transporter 5-like |  |  |  | 1.336814106 | 1.81E-06 |
| **Cs4g16770** | **Sulfate transporter 3,5 isoform 1** | **4.486832362** | **3.15E-83** |  | **6.263690581** | **2.24E-119** |
| Cs3g18650 | Sulfate transporter 1,3 isoform 1 | -1.832851322 | 6.53E-21 |  |  |  |
| **Cs5g30850** | **Sulfate transporter 3.1-like** | **-1.533121642** | **0.010108** |  | **1.29049926** | **1.13E-47** |
| Cs1g14530 | Sulfate/bicarbonate/oxalate exchanger and transporter sat-1 | 1.231052557 | 9.31E-8 |  |  |  |
| Cs9g07580 | Sulfate/bicarbonate/oxalate exchanger and transporter sat-1 | 2.078571297 | 1.04E-18 |  |  |  |
| **Cs1g15070** | **Sulfite exporter TauE/SafE family protein** | **1.411555146** | **1.70E-09** |  | **-1.196948974** | **9.84E-06** |
| Cs1g15090 | Sulfite exporter TauE/SafE family protein | 2.616647048 | 6.51E-09 |  |  |  |
| Cs2g04480 | Na^+^/Pi symporter | -1.753668955 | 8.40E-5 |  |  |  |
| Cs3g27660 | Phosphate transporter | 2.253809446 | 6.31E-22 |  |  |  |
| **Cs7g19830** | **Phosphate carrier protein, mitochondrial** | **1.642105882** | **9.93E-05** |  | **1.157367019** | **2.70E-08** |
| **Cs9g18560** | **Phosphate transporter** | **-3.890870837** | **3.53E-24** |  | **-2.397887581** | **2.11E-124** |
| Cs5g29860 | Phosphate transporter |  |  |  | 1.100392613 | 1.97E-51 |
| Cs1g06320 | Cyclic nucleotide-gated ion channel 4 isoform 1 | 1.464071926 | 3.69E-17 |  |  |  |
| Cs9g18130 | Cyclic nucleotide-gated ion channel 1-like | 1.112722743 | 0.041174 |  |  |  |
| Cs9g18200 | Cyclic nucleotide gated channel 1 isoform 1 | 1.200860993 | 0.000190 |  |  |  |
| orange1.1t00485 | Cyclic nucleotide-gated channel 13 | 1.27933816 | 1.66E-6 |  |  |  |
| Cs9g18180 | Cyclic nucleotide-gated ion channel 1-like |  |  |  | 4.333489752 | 7.38E-07 |
| Cs9g18250 | Cyclic nucleotide gated channel 1 isoform 3 |  |  |  | 1.11121191 | 4.06E-37 |
| Cs1g17345 | Cation calcium exchanger 4 |  |  |  | 1.538974052 | 0.020302 |
| Cs8g20150 | Ca^2+^ antiporter/cation exchanger |  |  |  | -1.31802215 | 0.034578 |
| **Cs9g01620** | **Probable boron transporter 2-like** | **-1.040907818** | **0.000417** |  | **1.004532572** | **4.29E-25** |
| Cs3g08550 | ATPase E1-E2 type family protein / haloacid dehalogenase-like hydrolase family protein | -2.190766774 | 0.001570 |  |  |  |
| Cs3g10680 | ATPase E1-E2 type family protein / haloacid dehalogenase-like hydrolase family protein | 2.659619621 | 3.60E-18 |  |  |  |
| Cs3g08600 | ATPase E1-E2 type family protein / haloacid dehalogenase-like hydrolase family protein |  |  |  | 1.127823084 | 0.048099 |
| **Cs5g08370** | **H^+^-ATPase 11 isoform 1** | **-4.787326267** | **1.42E-80** |  | **-2.388716417** | **1.02E-72** |
| Cs5g30450 | V-type proton ATPase subunit F-like isoform 1 |  |  |  | -1.168465295 | 4.14E-24 |
| **Cs5g30640** | **Calcium-transporting ATPase 12, plasma membrane-type-like** | **1.563843465** | **3.99E-12** |  | **1.032679141** | **2.46E-17** |
| **Cs4g03870** | **Cadmium/zinc-transporting ATPase 3 isoform 1** | **-1.760675459** | **6.71E-8** |  | **-2.25699064** | **1.45E-54** |
| Cs9g07790 | Autoinhibited calcium ATPase |  |  |  | 1.509187667 | 6.22E-34 |
| Cs1g13320 | Anion exchanger family protein | 2.353783937 | 0.001493 |  |  |  |
| Cs1g13690 | Pleiotropic drug resistance 12 | -1.105854767 | 1.84E-6 |  |  |  |
| **Cs1g18920** | **Pleiotropic drug resistance 12** | **-4.249792729** | **4.69E-17** |  | **-2.025179057** | **0.000366** |
| Cs4g20440 | Pleiotropic drug resistance protein 1-like | 1.355622457 | 4.42E-12 |  |  |  |
| **orange1.1t01531** | **Pleiotropic drug resistance 6** | **-3.384373211** | **4.89E-41** |  | **-1.59970784** | **6.34E-19** |
| Cs1g13620 | Pleiotropic drug resistance 12 |  |  |  | -1.004680144 | 0.045828 |
| Cs1g13660 | Pleiotropic drug resistance protein |  |  |  | -1.513979883 | 2.32E-92 |
| Cs5g13760 | Pleiotropic drug resistance protein 3-like |  |  |  | -2.468196115 | 9.09E-09 |
| Cs9g01400 | Pleiotropic drug resistance protein 3-like |  |  |  | -1.121025481 | 0.021097 |
| **Cs1g14580** | **Coatomer subunit beta'-1-like** | **3.193818702** | **3.42E-25** |  | **1.318114717** | **4.07E-7** |
| Cs1g15440 | Delta tonoplast integral protein | -1.02559856 | 4.56E-8 |  |  |  |
| Cs1g18400 | Calcineurin B-like protein 10 | 1.486744636 | 1.55E-12 |  |  |  |
| **Cs1g19190** | **Ammonium transporter 3 member 1-like** | **2.329176814** | **3.19E-24** |  | **1.082716457** | **3.69E-09** |
| Cs1g26160 | Ammonium transporter 3 member 1-like | -2.312707588 | 6.55E-6 |  |  |  |
| **Cs5g03120** | **Ammonium transporter** | **-5.61659445** | **1.96E-52** |  | **-2.530382962** | **4.97E-21** |
| **Cs5g25370** | **AMT1;2** | **-2.513481703** | **5.67E-27** |  | **-1.945118448** | **3.28E-109** |
| Cs5g25380 | Ammonium transporter | 1.658594191 | 2.18E-8 |  |  |  |
| **Cs5g30580** | **Ammonium transporter 2** | **-6.140282087** | **2.34E-104** |  | **-2.653031961** | **2.30E-61** |
| **Cs7g04990** | **Ammonium transporter 3 member 1-like** | **-4.659333928** | **2.68E-42** |  | **-2.48963924** | **7.40E-84** |
| orange1.1t01808 | Ammonium transporter 2 | -7.100223088 | 8.36E-22 |  |  |  |
| Cs3g10730 | Ammonium transporter 2 |  |  |  | -2.539512522 | 0.000190 |
| orange1.1t03479 | AMT1;1 |  |  |  | 1.03438369 | 7.50E-20 |
| **Cs2g01740** | **Oligopeptide transporter isoform 1** | **2.471455932** | **2.50E-35** |  | **1.371969176** | **1.10E-127** |
| Cs3g16870 | Oligopeptide transporter OPT family | -3.561480812 | 2.10E-20 |  |  |  |
| Cs2g04290 | Probable peptide/nitrate transporter At1g27040-like | -2.419526201 | 5.64E-33 |  |  |  |
| Cs5g29300 | Peptide transporter PTR3-A | -1.067052259 | 7.73E-5 |  |  |  |
| Cs2g05170 | Glutamate-gated kainate-type ion channel receptor subunit GluR5 | -1.888866272 | 1.79E-8 |  |  |  |
| Cs7g06960 | Glutamate receptor 2.9 | 1.024144312 | 0.031303 |  |  |  |
| Cs8g01200 | Glutamate receptor 2 isoform 1 | 1.205844953 | 4.32E-11 |  |  |  |
| Cs4g02830 | Glutamate receptor 2.7-like | 1.338854817 | 2.44E-8 |  |  |  |
| Cs1g26650 | Glutamate receptor isoform 1 |  |  |  | 1.283312364 | 9.45E-50 |
| Cs4g02850 | Glutamate receptor 2.7-like |  |  |  | 1.391192306 | 0.001729 |
| Cs7g07000 | Glutamate receptor 2.9 |  |  |  | 1.979962494 | 4.68E-06 |
| Cs9g07780 | ATP sulfurylase 1, chloroplastic-like |  |  |  | 1.861149273 | 0.034102 |
| Cs2g06890 | ARM repeat superfamily protein | 1.044723536 | 3.57E-11 |  |  |  |
| Cs9g07980 | ARM repeat superfamily protein isoform 2 |  |  |  | 1.647656074 | 8.61E-07 |
| Cs2g10420 | Gamma-soluble NSF attachment protein isoform 1 | 1.228352288 | 4.68E-10 |  |  |  |
| **Cs2g10720** | **Zinc transporter 10 precursor** | **-2.854554142** | **5.27E-28** |  | **-2.384376328** | **2.25E-163** |
| **Cs2g11620** | **Zinc transporter 1-like** | **-5.379598509** | **4.69E-22** |  | **-3.891118942** | **3.78E-22** |
| **Cs4g18450** | **Zinc transporter 4** | **-2.171419214** | **2.94E-29** |  | **-1.775461502** | **6.39E-73** |
| Cs2g11610 | Zinc transporter 5 isoform 1 | 1.506555697 | 4.99E-8 |  |  |  |
| Cs6g11460 | Zinc transporter 5 precursor |  |  |  | -2.06354964 | 1.17E-11 |
| Cs8g20480 | ZIP transporter |  |  |  | -1.367827575 | 5.14E-26 |
| Cs8g18810 | ZRT/IRT-like protein 2 isoform 1 |  |  |  | -1.662871349 | 1.03E-37 |
| **Cs8g19450** | **Copper transporter 1-like** | **-3.52069094** | **0.000194** |  | **-4.264501629** | **2.01E-08** |
| **Cs8g19460** | **Copper transporter 1-like** | **-1.920714382** | **1.31E-7** |  | **-4.097763691** | **8.12E-61** |
| **Cs8g19470** | **Copper transporter 1-like** | **-1.805665736** | **0.005077** |  | **-3.164356502** | **5.87E-38** |
| Cs4g04460 | Sodium transporter hkt1-like protein |  |  |  | -1.329817375 | 7.71E-10 |
| Cs2g11770 | Cationic amino acid transporter 5 | -1.173347941 | 8.22E-5 |  |  |  |
| Cs2g11840 | Vacuolar sorting receptor 7 | 1.022565363 | 1.96E-9 |  |  |  |
| Cs2g30260 | Dicarboxylate transporter 1 isoform 1 | -1.140295572 | 3.16E-10 |  |  |  |
| **Cs3g12520** | **Sugar transporter ERD6-like 5-like** | **1.370328482** | **0.008493** |  | **-1.192245246** | **0.008894** |
| **Cs3g16120** | **Exocyst subunit exo70 family protein G1** | **-4.221233453** | **6E-35** |  | **-1.261084856** | **1.95E-06** |
| Cs6g22190 | Exocyst subunit exo70 family protein E1 | -1.204583987 | 2.73E-8 |  |  |  |
| Cs8g17810 | Exocyst complex component | -2.77787992 | 4.58E-24 |  |  |  |
| Cs8g20880 | Exocyst subunit exo70 family protein B1 | -1.01249397 | 7.58E-6 |  |  |  |
| orange1.1t01333 | Exocyst subunit exo70 family protein G1 |  |  |  | 1.105091407 | 9.80E-05 |
| Cs3g16570 | Amino acid permease 6 | -1.278924151 | 1.96E-9 |  |  |  |
| Cs4g19070 | Amino acid permease | 8.845817756 | 0.008580 |  |  |  |
| **Cs8g02760** | **Amino acid permease 2-like** | **-1.034894418** | **0.000492** |  | **-1.816054595** | **1.21E-22** |
| Cs4g07790 | Amino acid permease isoform 2 |  |  |  | -1.174713594 | 1.54E-16 |
| Cs4g07810 | Amino acid permease |  |  |  | -1.138454203 | 0.009047 |
| Cs6g21320 | Amino acid transporter isoform 2 | -2.189436925 | 7.99E-23 |  |  |  |
| Cs6g21330 | Amino acid transporter isoform 2 | 1.836223303 | 0.000714 |  |  |  |
| Cs7g03780 | Amino acid transporter |  |  |  | -1.660054337 | 7.30E-15 |
| Cs8g02010 | Transmembrane amino acid transporter family protein |  |  |  | 1.898337341 | 8.24E-132 |
| Cs3g17390 | Mitochondrial uncoupling protein 5-like | -1.944772738 | 2.21E-22 |  |  |  |
| Cs3g19250 | Auxin influx carrier protein | -1.114201778 | 1.57E-5 |  |  |  |
| Cs3g22560 | Citrus sucrose transporter 1 | -1.078701131 | 9.42E-6 |  |  |  |
| Cs4g15780 | Polyol transporter 5-like | 1.090416038 | 5.16E-6 |  |  |  |
| Cs4g16170 | Probable peptide transporter At1g52190 | 2.298123788 | 9.82E-18 |  |  |  |
| Cs5g01190 | Heavy metal transport/detoxification superfamily protein | 1.260844562 | 1.28E-9 |  |  |  |
| Cs3g18690 | Heavy metal transport/detoxification superfamily protein |  |  |  | -1.141415613 | 9.05E-09 |
| Cs5g03070 | Heavy metal transport/detoxification superfamily protein isoform 1 |  |  |  | -1.56178973 | 1.61E-61 |
| XLOC_027092 | Heavy metal transport/detoxification superfamily protein |  |  |  | -2.128702961 | 0.00084 |
| Cs5g03430 | Divalent ion symporter | 4.49307061 | 5.52E-6 |  |  |  |
| Cs9g17690 | Divalent ion symporter isoform 1 | 1.679709455 | 1.59E-22 |  |  |  |
| Cs5g04280 | Nitrate transporter 1:2 isoform 3 | -1.620106522 | 1.41E-6 |  |  |  |
| **Cs5g06250** | **Nitrate transmembrane transporters** | **-1.415005282** | **2.99E-13** |  | **-1.002768769** | **3.93E-57** |
| Cs7g13200 | Probable nitrite transporter At1g68570-like | 1.992095309 | 1.49E-28 |  |  |  |
| **Cs7g22760** | **Nitrate transporter 1.5-like** | **1.37163952** | **5.58E-12** |  | **-1.344048784** | **5.63E-55** |
| **Cs8g16010** | **Nitrate transporter** | **-1.882755655** | **1.20E-21** |  | **-2.222334916** | **1.07E-128** |
| Cs5g09050 | Nitrate transporter 1.1 isoform 1 |  |  |  | 1.014667039 | 5.70E-34 |
| orange1.1t02415 | Nitrate transporter 2.4 |  |  |  | -1.181456295 | 6.31E-10 |
| Cs5g06420 | Proline transporter 1-lik | 1.809373032 | 0.007303 |  |  |  |
| **Cs5g08710** | **Membrane channel protein** | **-1.609648119** | **4.12E-15** |  | **-1.91828791** | **3.56E-223** |
| **Cs6g11660** | **Plasma intrinsic protein 2,2** | **-3.274963342** | **5.91E-16** |  | **-1.274196489** | **1.97E-20** |
| **Cs6g11690** | **Plasma intrinsic protein 2,2** | **-3.508841491** | **3.72E-41** |  | **-1.689532862** | **5.67E-72** |
| **Cs6g11700** | **Plasma intrinsic protein 2,2** | **-3.370844464** | **1.42-30** |  | **-1.335301715** | **4.55E-54** |
| Cs7g02180 | Plasma-membrane choline transporter family protein isoform 1 | 1.288886522 | 2.48E-9 |  |  |  |
| Cs8g02530 | Plasma membrane intrinsic protein 3 isoform 2, partial | -1.085313167 | 2.66E-7 |  |  |  |
| Cs7g04650 | Vps51/Vps67 family (components of vesicular transport) protein | -9.459454161 | 4.60E-8 |  |  |  |
| Cs7g23090 | Cationic amino acid transporter | -1.214415861 | 2.48E-5 |  |  |  |
| Cs8g06810 | Mannitol transporter | 1.016988101 | 7.58E-6 |  |  |  |
| Cs8g06820 | Mannitol transporter | 1.79048611 | 4.22E-18 |  |  |  |
| orange1.1t05405 | Mannitol transporter | 1.911520807 | 0.024451 |  |  |  |
| orange1.1t05715 | Mannitol transporter | 1.443345864 | 3.69E-6 |  |  |  |
| Cs4g15770 | Polyol/monosaccharide transporter 5 isoform 1 | 1.048960163 | 7.22E-05 |  |  |  |
| orange1.1t04492 | Vacuolar cation/proton exchanger 2 | 4.464418734 | 2.69E-32 |  |  |  |
| Cs3g17390 | Mitochondrial uncoupling protein 5-like | -1.944772738 | 2.21E-22 |  |  |  |
| **Cs8g20830** | **Cation/H^+^ antiporter 24** | **-2.907676417** | **7.78E-8** |  | **-1.300446948** | **0.033383** |
| Cs1g18890 | Solute carrier family 22 member 3-like |  |  |  | -1.444538893 | 3.51E-42 |
| XLOC_028937 | Solute carrier family 22 member 12 |  |  |  | -1.146363139 | 4.99E-05 |
| Cs2g01380 | NF-X1-type zinc finger protein NFXL1-like |  |  |  | 1.405684243 | 3.76E-5 |
| Cs3g04570 | Transport protein sec24-like CEF |  |  |  | -1.747226543 | 6.62E-5 |
| Cs3g14500 | Nodulin MtN3 family protein isoform 1 |  |  |  | -1.767102114 | 6.68E-5 |
| Cs3g14550 | Nodulin MtN3 family protein isoform 1 |  |  |  | -1.21697945 | 0.001334 |
| Cs3g19460 | Nodulin MtN21 /EamA-like transporter family protein |  |  |  | -1.221058375 | 3.38E-82 |
| Cs9g18460 | Nodulin MtN3 family protein |  |  |  | 10.05003024 | 0.015257 |
| Cs9g17980 | Got1/Sft2-like vescicle transport protein family |  |  |  | 1.028493348 | 0.000935 |
| **orange1.1t00127** | **STR** | **-2.30890287** | **1.25E-24** |  | **-1.645591401** | **1.86E-37** |
| orange1.1t00417 | Intracellular protein transport protein USO1-like |  |  |  | 1.119247879 | 0.004661 |
| orange1.1t02271 | UDP-galactose transporter 2-like |  |  |  | 1.20681268 | 1.58E-22 |
| Cs2g05530 | Glucose-6-phosphate/phosphate translocator-related |  |  |  | 1.210614127 | 5.55E-13 |

*DEGs shared by the two citrus species were highlighted in bold. FC, fold change; FDR, false discovery rate.*

**TABLE S6 | Low-P-responsive genes in Al-treated *C. sinensis* and *C. grandis* roots.**

| **ID** | **Description** | ***C. grandis*** | |  | ***C. sinensis*** | |  |
| --- | --- | --- | --- | --- | --- | --- | --- |
|  |  | **log2 (FC)** | **FDR** |  | **log2 (FC)** | **FDR** |  |
| Cs1g12410 | Phosphatidic acid phosphatase-related protein | -1.615872706 | 6.87E-17 |  |  |  |  |
| Cs5g27880 | Purple acid phosphatase 15 isoform 1 | 1.113756518 | 5.66E-11 |  |  |  |  |
| **Cs7g02530** | **Purple acid phosphatase 27 isoform 1** | **-4.248133286** | **9.54E-34** |  | **-4.0277364** | **1.38E-24** |  |
| **Cs8g08070** | **Purple acid phosphatase** | **1.036537653** | **0.009990** |  | **1.608303262** | **0.012876** |  |
| Cs8g11340 | Purple acid phosphatase 23 isoform 1 | 1.453821125 | 1.16E-07 |  |  |  |  |
| **orange1.1t02445** | **Purple acid phosphatase 15-like** | **1.357985009** | **1.14E-09** |  | **1.332273144** | **7.75E-09** |  |
| Cs8g08300 | Purple acid phosphatase 2 isoform 1 |  |  |  | 1.135016709 | 6.83E-21 |  |
| Cs3g25470 | Purple acid phosphatase 12 isoform 1 | 2.563558394 | 2.94E-17 |  | 1.274248065 | 2.37E-06 |  |
| Cs8g08310 | Purple acid phosphatase 10 |  |  |  | 1.186619334 | 2.24E-27 |  |
| orange1.1t02668 | Purple acid phosphatase 17 isoform 1 |  |  |  | 1.505686509 | 0.000424 |  |
| Cs3g27520 | Glyceraldehyde-3-phosphate dehydrogenase B subunit | -1.814343002 | 0.039425 |  |  |  |  |
| Cs2g14940 | Glyceraldehyde-3-phosphate dehydrogenase | 3.856383691 | 1.50E-09 |  |  |  |  |
| **Cs4g01490** | **NADP-dependent glyceraldehyde-3-phosphate dehydrogenase-like** | **2.845271187** | **2.80E-13** |  | **1.23105656** | **2.06E-05** |  |
| **Cs5g30520** | **Pyruvate kinase isozyme A, chloroplastic isoform 1** | **-1.854935055** | **0.007858** |  | **1.11623874** | **6.43E-12** |  |
| Cs7g02950 | Glycerophosphodiester phosphodiesterase GDE1 | 2.276277943 | 2.38E-21 |  |  |  |  |
| **Cs7g03770** | **Ribonuclease T2** | **1.924069854** | **5.09E-18** |  | **1.489860265** | **4.23E-28** |  |
| Cs7g22250 | Haloacid dehalogenase-like hydrolase (HAD) superfamily protein | 1.259505882 | 9.52E-12 |  |  |  |  |
| Cs2g11750 | Haloacid dehalogenase-like hydrolase domain-containing protein 3-like |  |  |  | 1.466015228 | 2.33E-50 |  |
| Cs8g18750 | Haloacid dehalogenase-like hydrolase superfamily protein |  |  |  | 1.176986581 | 9.55E-23 |  |
| Cs8g18760 | Haloacid dehalogenase-like hydrolase superfamily protein |  |  |  | 2.026074232 | 5.13E-43 |  |
| Cs8g12690 | Phosphoenolpyruvate carboxylase kinase 1 | 2.276846958 | 3.88E-28 |  |  |  |  |
| Cs3g16700 | Phosphoenolpyruvate carboxykinase [ATP]-like |  |  |  | -1.076974408 | 1.64E-39 |  |
| Cs1g15970 | 6-phosphofructokinase 3 |  |  |  | 1.223502829 | 4.48E-13 |  |
| Cs4g13070 | 6-phosphofructokinase 3 |  |  |  | 1.134091069 | 1.62E-85 |  |
| Cs3g03130 | Alkaline-phosphatase-like family protein isoform 1 |  |  |  | 1.062645397 | 5.20E-21 |  |
| Cs2g04480 | Na^+^/Pi symporter | -1.753668955 | 8.40E-5 |  |  |  |  |
| Cs3g27660 | Phosphate transporter | 2.253809446 | 6.31E-22 |  |  |  |  |
| **Cs9g18560** | **Phosphate transporter** | **-3.890870837** | **3.53E-24** |  | **-2.397887581** | **2.11E-124** |  |
| Cs5g29860 | Phosphate transporter |  |  |  | 1.100392613 | 1.97E-51 |  |
| **Cs7g19830** | **Phosphate carrier protein, mitochondrial** | **1.642105882** | **9.93E-05** |  | **1.157367019** | **2.70E-08** |  |
| **Cs4g17870** | **SPX domain gene 2** | **3.463223553** | **1.97E-70** |  | **1.556103893** | **1.97E-70** |  |
| orange1.1t00194 | SPX domain-containing protein 3-like | 3.15105559 | 2.23E-59 |  |  |  |  |
| Cs6g13210 | SPX (SYG1/Pho81/XPR1) domain-containing protein / zinc finger (C3HC4-type RING finger) protein-related | 1.472200061 | 0.011547 |  |  |  |  |

*DEGs shared by the two citrus species were highlighted in bold. FC, fold change; FDR, false discovery rate.*

**TABLE S7 | DEGs related to S transport and metabolism in Al-treated *C. sinensis* and *C. grandis* roots.**

| **ID** | **Description** | ***C. grandis*** | |  | ***C. sinensis*** | |
| --- | --- | --- | --- | --- | --- | --- |
|  |  | **log2 (FC)** | **FDR** |  | **log2 (FC)** | **FDR** |
| Transport |  |  |  |  |  |  |
| **Cs4g16770** | **Sulfate transporter 3,5 isoform 1** | **4.486832362** | **3.15E-83** |  | **6.263690581** | **2.24E-119** |
| Cs3g18650 | Sulfate transporter 1,3 isoform 1 | -1.832851322 | 6.53E-21 |  |  |  |
| **Cs5g30850** | **Sulfate transporter 3.1-like** | **-1.533121642** | **0.010108** |  | **1.29049926** | **1.13E-47** |
| Cs1g14530 | Sulfate/bicarbonate/oxalate exchanger and transporter sat-1 | 1.231052557 | 9.31E-8 |  |  |  |
| Cs9g07580 | Sulfate/bicarbonate/oxalate exchanger and transporter sat-1 | 2.078571297 | 1.04E-18 |  |  |  |
| **Cs1g15070** | **Sulfite exporter TauE/SafE family protein** | **1.411555146** | **1.70E-09** |  | **-1.196948974** | **9.84E-06** |
| Cs1g15090 | Sulfite exporter TauE/SafE family protein | 2.616647048 | 6.51E-09 |  |  |  |
| Cs3g21890 | S-adenosylmethionine carrier 1 | 1.278881102 | 0.000272 |  |  |  |
|  |  |  |  |  |  |  |
| Metabolism |  |  |  |  |  |  |
| Cs9g07780 | ATP sulfurylase 1, chloroplastic-like |  |  |  | 1.861149273 | 0.034102 |
| **Cs1g16430** | **Sulfotransferase 2A** | **-2.072522162** | **0.013232** |  | **1.890201213** | **2.10E-45** |
| Cs2g02980 | 5'-adenylylsulfate reductase 3, chloroplastic-like isoform 1 | -1.733194029 | 1.22E-19 |  |  |  |
| Cs9g16940 | S-adenosyl-L-methionine-dependent methyltransferases superfamily protein |  |  |  | 1.358641447 | 6.22E-26 |
| orange1.1t00081 | S-adenosyl-L-methionine-dependent methyltransferases superfamily protein isoform 1 |  |  |  | 1.029244146 | 3.44E-48 |
| orange1.1t00305 | S-adenosyl-L-methionine-dependent methyltransferases superfamily protein isoform 1 |  |  |  | 1.058307514 | 4.35E-15 |
| orange1.1t00778 | S-adenosylmethionine-dependent methyltransferase |  |  |  | 1.916940318 | 6.57E-06 |
| **orange1.1t00787** | **S-adenosylmethionine-dependent methyltransferase** | **1.797792** | **0.002429** |  | **2.46483072** | **6.64E-07** |
| orange1.1t02389 | S-adenosylmethionine-dependent methyltransferase |  |  |  | 1.114325099 | 1.68E-29 |
| Cs4g01110 | S-adenosyl-L-methionine-dependent methyltransferases superfamily protein | -1.131253167 | 0.000561 |  |  |  |
| XLOC_029805 | S-adenosyl-L-methionine-dependent methyltransferases superfamily protein | 2.207482315 | 0.049632 |  |  |  |
| orange1.1t04948 | S-adenosyl-L-methionine-dependent methyltransferases superfamily protein | 3.382865067 | 0.018310 |  |  |  |
| Cs9g01410 | S-adenosylmethionine synthetase | -1.201982333 | 2.23E-08 |  |  |  |
| Cs4g02260 | S-adenosylmethionine decarboxylase proenzyme-like | -1.271311845 | 3.86E-09 |  |  |  |
| orange1.1t03455 | Glutathione S-transferase tau 7 |  |  |  | 1.366040005 | 3.13E-25 |
| orange1.1t03618 | Glutathione transferase, partial |  |  |  | 1.153170884 | 1.86E-06 |
| orange1.1t03629 | Glutathione S-transferase tau 7 |  |  |  | 1.379843931 | 2.12E-21 |
| **orange1.1t05889** | **Glutathione transferase, partial** | **1.057212874** | **0.037916** |  | **1.706044866** | **7.93E-12** |
| **Cs8g19400** | **Glutathione S-transferase U7 isoform 1** | **2.107755884** | **6.76E-17** |  | **1.125558225** | **1.53E-10** |
| **Cs7g14120** | **Probable glutathione S-transferase** | **-5.216932383** | **5.55E-57** |  | **-3.404722761** | **2.36E-13** |
| **Cs7g14180** | **Probable glutathione S-transferase** | **-3.184121145** | **1.50E-10** |  | **-2.109314431** | **4.59E-10** |
| **Cs7g14300** | **Glutathione S-transferase TAU 19** | **-2.521433132** | **0.020104** |  | **-4.212637493** | **0.000458** |
| **orange1.1t04722** | **Probable glutathione S-transferase** | **-3.948785197** | **1.14E-25** |  | **-2.727568454** | **1.24E-32** |
| **orange1.1t04916** | **Glutathione S-transferase TAU 19** | **-4.857716426** | **2.51E-58** |  | **-3.038463665** | **5.42E-63** |
| Cs5g32780 | Phi class glutathione transferase GSTF3 | -1.079293401 | 0.044488 |  |  |  |
| orange1.1t00238 | Glutathione S-transferase zeta-class 1 isoform 1 | 1.173982378 | 8.75E-06 |  |  |  |
| orange1.1t03456 | Glutathione transferase, partial | 8.28747 | 9.56E-09 |  |  |  |
| orange1.1t03610 | Glutathione S-transferase tau 7 | 1.121912443 | 0.020273 |  |  |  |
| Cs2g11200 | Sulfiredoxin isoform 2 |  |  |  | -1.296916049 | 1.48E-05 |
| Cs5g35220 | Peptide methionine sulfoxide reductase B3, chloroplastic-like |  |  |  | -1.252797929 | 0.000500 |
| Cs8g03105 | Peptide methionine sulfoxide reductase B3, chloroplastic-like |  |  |  | -1.026952554 | 1.12E-08 |
| Cs1g26070 | Protein-methionine-s-oxide reductase | -1.02741367 | 3.15E-06 |  |  |  |
| Cs9g05400 | Peptide methionine sulfoxide reductase msrB | 1.115693 | 6.44E-10 |  |  |  |
| **Cs9g07420** | **Homocysteine S-methyltransferase** | **-1.104352292** | **7.98E-07** |  | **-1.223863343** | **6.89E-40** |
| Cs7g13320 | Homocysteine S-methyltransferase 1 | -1.102982387 | 0.037034 |  |  |  |
| Cs9g05680 | Glutamine synthetase 2 | -1.812197633 | 2.40E-20 |  |  |  |
| Cs8g12450 | Class I glutamine amidotransferase-like superfamily protein | -1.225231937 | 4.36E-09 |  |  |  |
| Cs4g02230 | Class I glutamine amidotransferase-like superfamily protein | 2.247823272 | 0.009319 |  |  |  |
| Cs9g17990 | NADH-dependent glutamate synthase 1 isoform 1 | -1.371926726 | 1.44E-08 |  |  |  |
| Cs4g13930 | Atypical CYS HIS rich thioredoxin 2 | 1.015838563 | 0.000999 |  |  |  |
| Cs8g12640 | Threonine aldolase 1 isoform 1 | 1.416904015 | 1.47E-10 |  |  |  |
|  |  |  |  |  |  |  |
| Cs8g06060 | Thioredoxin superfamily protein | -1.100767683 | 0.000983 |  |  |  |
| orange1.1t02220 | WCRKC thioredoxin 1 isoform 1 | 1.178865321 | 0.000121 |  |  |  |
| Cs1g26810 | NADPH-dependent thioredoxin reductase A | -2.220400659 | 1.33E-18 |  |  |  |
| Cs2g05460 | Thioredoxin superfamily protein | -1.496814146 | 1.10E-07 |  |  |  |
| Cs2g16150 | Glutaredoxin | -1.639314567 | 5.54E-14 |  |  |  |
| Cs2g13810 | Glutaredoxin-C1 | -1.626638075 | 0.003253 |  |  |  |
| Cs2g31250 | Serine-glyoxylate aminotransferase-like | -2.591834882 | 2.00E-14 |  |  |  |
| Cs4g13090 | Gamma-glutamyl transpeptidase 4 | -1.128074035 | 0.002196 |  |  |  |
| Cs5g03060 | ACC synthase | -1.135367249 | 4.77E-07 |  |  |  |
| orange1.1t00416 | ACC synthase |  |  |  | 1.151969425 | 0.000633 |

*DEGs shared by the two citrus species were highlighted in bold. FC, fold change; FDR, false discovery rate*.

**TABLE S8 | DEGs related to antioxidation and detoxification in Al-treated *C. sinensis* and *C. grandis* roots.**

| **ID** | **Description** | ***C. grandis*** | |  | ***C. sinensis*** | |
| --- | --- | --- | --- | --- | --- | --- |
|  |  | **log2 (FC)** | **FDR** |  | **log2 (FC)** | **FDR** |
| Cs2g11900 | Peroxidase 7-like |  |  |  | 1.351073133 | 0.003408 |
| Cs3g02270 | Lignin-forming anionic peroxidase-like |  |  |  | 2.401960078 | 8.46E-05 |
| Cs3g02280 | Lignin-forming anionic peroxidase-like |  |  |  | 1.874601919 | 1.65E-06 |
| Cs3g02310 | Lignin-forming anionic peroxidase-like |  |  |  | 1.7868186 | 2.17E-31 |
| Cs3g26600 | Peroxidase 64-like isoform 1 |  |  |  | 1.12380635 | 1.04E-12 |
| Cs4g03740 | Peroxidase 45-like |  |  |  | 1.14351271 | 1.76E-45 |
| Cs6g09680 | Peroxidase superfamily protein |  |  |  | 1.102017114 | 1.33E-30 |
| Cs7g13530 | Peroxidase 51-like |  |  |  | 1.633145383 | 2.33E-05 |
| Cs7g20700 | Peroxidase N1 |  |  |  | 1.514079389 | 1.59E-35 |
| **Cs9g05130** | **Peroxidase superfamily protein** | **1.325344149** | **0.000638** |  | **1.352233162** | **2.12E-59** |
| **orange1.1t05402** | **Cationic peroxidase 1-like** | **2.018385382** | **4.96E-07** |  | **1.47147719** | **2.29E-31** |
| orange1.1t02033 | Peroxidase superfamily protein | 1.548796734 | 9.30E-18 |  |  |  |
| orange1.1t02038 | Peroxidase superfamily protein | 1.622027219 | 6.03E-05 |  |  |  |
| orange1.1t02225 | Peroxidase precursor | 1.366050443 | 6.43E-06 |  |  |  |
| Cs1g25930 | Peroxidase 64 | -2.183597275 | 0.001307 |  |  |  |
| Cs2g03110 | Secretory peroxidase | -1.063358765 | 2.37E-05 |  |  |  |
| Cs3g20770 | Peroxidase | -8.350742537 | 0.047100 |  |  |  |
| Cs9g16590 | Peroxidase 66-like | -1.116915267 | 0.004739 |  |  |  |
| Cs8g10680 | Peroxiredoxin-2B-like |  |  |  | 3.862115785 | 1.79E-05 |
| Cs1g17550 | Germin-like protein |  |  |  | 1.5821396 | 1.03E-07 |
| Cs3g04870 | Germin-like protein subfamily T member 2-like |  |  |  | 3.421406709 | 1.97E-11 |
| Cs3g04880 | Germin-like protein subfamily T member 2 |  |  |  | 1.112215817 | 8.25E-11 |
| Cs5g25660 | Germin-like protein |  |  |  | 2.651747007 | 0.014349 |
| **XLOC_021846** | **Germin-like protein 9-3** | **-2.151597185** | **5.32E-17** |  | **1.30712287** | **6.96E-17** |
| **Cs5g07750** | **Germin-like protein 9-3** | **-1.730381807** | **1.13E-09** |  | **-1.485274696** | **0.002502** |
| **Cs7g20270** | **Germin-like protein 9-3** | **-4.067189815** | **1.05E-23** |  | **-2.510835241** | **9.34E-09** |
| **XLOC_020823** | **Germin-like protein 9-3** | **-5.410815085** | **4.78E-76** |  | **-5.093280746** | **0** |
| Cs5g14180 | Germin-like protein subfamily 1 member 14 | 2.139139926 | 2.75E-17 |  |  |  |
| Cs5g14210 | Germin-like protein subfamily 1 member 14 | 2.162281585 | 7.15E-19 |  |  |  |
| Cs5g14230 | Germin-like protein subfamily 1 member 14 | 1.437582719 | 1.66E-10 |  |  |  |
| Cs5g14290 | Germin-like protein subfamily 1 member 14 | 1.723789053 | 3.29E-05 |  |  |  |
| Cs5g14300 | Germin-like protein subfamily 1 member 14 | 2.991859602 | 7.35E-08 |  |  |  |
| Cs5g14330 | Germin-like protein subfamily 1 member 18 | 1.150844167 | 0.000221 |  |  |  |
| Cs5g25740 | Germin-like protein | 2.785272181 | 7.51E-05 |  |  |  |
| Cs5g25760 | Germin-like protein | 2.742205863 | 0.001300 |  |  |  |
| Cs5g25810 | Germin-like protein subfamily 1 member 14 | 1.106423771 | 3.60E-11 |  |  |  |
| Cs6g09150 | Ferretin 1 isoform 1 | 1.422260344 | 1.71E-14 |  |  |  |
| Cs3g20800 | Alcohol dehydrogenase |  |  |  | 2.313098306 | 5.29E-218 |
| Cs3g20810 | Alcohol dehydrogenase |  |  |  | 1.832569477 | 2.25E-41 |
| orange1.1t04627 | Alcohol dehydrogenase class-3 |  |  |  | -1.2311327 | 2.24E-05 |
| Cs2g30190 | Aldehyde dehydrogenase family 3 member F1 |  |  |  | -2.427091434 | 0.003446 |
| Cs4g16680 | Aldehyde dehydrogenase family 2 member C4-like |  |  |  | -1.876501972 | 0.012321 |
| Cs8g10170 | Aldo/keto reductase AKR | -1.241911206 | 2.90E-05 |  |  |  |
| Cs5g01410 | Beta-carotene isomerase D27, chloroplastic-like |  |  |  | 1.47906182 | 5.63E-06 |
| orange1.1t02108 | Phytoene synthase 2, chloroplastic-like |  |  |  | 1.231103531 | 2.25E-12 |
| **orange1.1t02112** | **Phytoene synthase 2, chloroplastic-like** | **4.18548374** | **4.92E-40** |  | **1.74823836** | **8.56E-08** |
| Cs8g18780 | ABA 8'-hydroxylase |  |  |  | 2.239994415 | 1.72E-05 |
| **Cs3g21210** | **Abscisic acid 8'-hydroxylase 3-like** | **-2.750644643** | **3.63E-24** |  | **-1.911080681** | **2.21E-27** |
| **Cs1g25490** | **Abscisic acid 8'-hydroxylase 4** | **1.812264984** | **6.07E-05** |  |  |  |
| orange1.1t02361 | Phytoene desaturase | 1.047692366 | 4.74E-08 |  |  |  |
| Cs3g11040 | Zeta-carotene desaturase | 2.336927494 | 3.01E-05 |  |  |  |
| Cs3g11170 | Zeta-carotene desaturase | 1.067943203 | 3.46E-08 |  |  |  |
| Cs3g11180 | Zeta-carotene desaturase | 1.017248474 | 1.94E-09 |  |  |  |
| Cs3g13040 | ZDS | 1.680327462 | 0.005202 |  |  |  |
| orange1.1t06069 | ZDS | 10.9260884 | 0.016971 |  |  |  |
| Cs5g24730 | 15-cis-zeta-carotene isomerase | 1.04583848 | 1.65E-07 |  |  |  |
| Cs4g14850 | Lycopene epsilon-cyclase | 1.585338621 | 0.002835 |  |  |  |
| Cs4g19460 | Carotenoid cleavage dioxygenase 8, chloroplastic-like | 2.331282466 | 3.42E-29 |  |  |  |
| Cs4g19530 | Carotenoid 9,10(9',10')-cleavage dioxygenase 1-like | 2.241975873 | 0.002280 |  |  |  |
| Cs5g26080 | Violaxanthin de-epoxidase | 1.737842634 | 0.001341 |  |  |  |
| Cs2g03270 | 9-cis-epoxycarotenoid dioxygenase 2 | -1.11455966 | 0.003760 |  |  |  |
| orange1.1t04051 | Zeaxanthin epoxidase | -4.678249955 | 2.55E-09 |  |  |  |

*DEGs shared by the two citrus species were highlighted in bold. FC, fold change; FDR, false discovery rate.*

**TABLE S9 | DEGs related to polysaccharide, cell wall and cytoskeleton metabolism in Al-treated *C. sinensis* and *C. grandis* roots.**

| **ID** | **Description** | ***C. grandis*** | |  | ***C. sinensis*** | |
| --- | --- | --- | --- | --- | --- | --- |
|  |  | **log2(FC)** | **FDR** |  | **log2(FC)** | **FDR** |
| Polysaccharide and cell wall metabolism | | |  |  |  |  |
| Cs1g05830 | Callose synthase 5-like | 1.178261755 | 0.000331 |  |  |  |
| Cs1g16550 | Pectinesterase | -1.364945602 | 2.67E-09 |  |  |  |
| Cs1g16560 | Pectinesterase 3 | -1.825688909 | 1.01E-21 |  |  |  |
| Cs2g16380 | Pectinesterase 11 | 1.741392301 | 1.26E-05 |  |  |  |
| **orange1.1t00214** | **Pectinesterase** | **1.211806582** | **2.16E-06** |  | **1.194702239** | **1.21E-39** |
| orange1.1t01727 | Pectinesterase |  |  |  | 2.335451805 | 0.014109 |
| Cs5g33450 | Probable pectinesterase/pectinesterase inhibitor 40-like | -1.604712917 | 0.000216 |  |  |  |
| **Cs3g06900** | **Probable pectinesterase/pectinesterase inhibitor 25-like** | **-1.472301701** | **0.001433** |  | **1.108846385** | **1.77E-12** |
| Cs4g06650 | Pectinesterase/pectinesterase inhibitor PPE8B | -1.55063 | 7.14E-06 |  |  |  |
| Cs4g06630 | Pectin methylesterase 3 | -1.88872 | 0.012377 |  |  |  |
| Cs4g06670 | Plant invertase/pectin methylesterase inhibitor superfamily | -1.07215 | 0.001391 |  |  |  |
| Cs4g06690 | Plant invertase/pectin methylesterase inhibitor superfamily | -1.17736 | 0.000101 |  |  |  |
| orange1.1t02719 | Plant invertase/pectin methylesterase inhibitor superfamily isoform 1 | -1.188123505 | 2.51E-08 |  |  |  |
| Cs5g33420 | Plant invertase/pectin methylesterase inhibitor superfamily |  |  |  | 2.08415309 | 4.85E-179 |
| Cs8g11330 | Pectate lyase 1-27 | -1.160311566 | 1.19E-09 |  |  |  |
| orange1.1t01738 | Pectin lyase-like superfamily protein | -1.869961607 | 0.002684 |  |  |  |
| Cs7g21940 | Pectin lyase-like superfamily protein isoform 1 | -1.282095628 | 6.40E-12 |  |  |  |
| Cs2g01430 | Galacturonosyltransferase 4 isoform 1 | -1.044704446 | 2.81E-06 |  |  |  |
| Cs5g33680 | Galacturonosyltransferase-like 7 isoform 1 | -1.279256107 | 5.07E-11 |  |  |  |
| Cs3g24380 | Glycosyltransferase family GT8 protein | -2.03861548 | 7.08E-28 |  |  |  |
| Cs7g16400 | Xylogalacturonan beta-1,3-xylosyltransferase | 1.044149414 | 2.48E-08 |  |  |  |
| Cs2g10560 | Exopolygalacturonase-like | -1.020774279 | 0.025491 |  |  |  |
| **Cs2g02960** | **Probable rhamnogalacturonate lyase B-like** | **-2.796355868** | **6.03E-08** |  | **-1.869757959** | **0.000202** |
| Cs3g16950 | Probable rhamnogalacturonate lyase B-like |  |  |  | 1.385206969 | 0.007909 |
| Cs2g04590 | Cellulose synthase A1 | -1.107338326 | 1.88E-06 |  |  |  |
| Cs3g09000 | Cellulose synthase-like D3 | -2.527339995 | 9.97E-27 |  |  |  |
| Cs3g16380 | Cellulose synthase-like protein | -1.703764018 | 4.52E-16 |  |  |  |
| Cs4g01990 | Cellulose synthase 3 | -1.380278282 | 3.42E-10 |  |  |  |
| Cs5g33670 | Cellulose synthase-like D5 | -1.317639939 | 1.34E-06 |  |  |  |
| orange1.1t04027 | Cellulose synthase | -2.717544338 | 5.35E-05 |  |  |  |
| orange1.1t05015 | Cellulose synthase like E1 isoform 1 | 1.174927894 | 0.003095 |  |  |  |
| Cs4g02000 | Cellulose synthase 3 |  |  |  | 3.658821354 | 0.020231 |
| Cs8g01130 | Cellulose synthase-like protein D4-like |  |  |  | -1.257141446 | 0.004893 |
| Cs5g01400 | Acidic cellulase |  |  |  | -2.012434367 | 0.005437 |
| Cs4g03050 | Xyloglucan endotransglucosylase/hydrolase family protein | -2.4203 | 1.14E-17 |  |  |  |
| Cs4g03060 | Xyloglucan endotransglucosylase/hydrolase family protein | -1.81539 | 4.07E-12 |  |  |  |
| Cs4g03130 | Xyloglucan endotransglucosylase/hydrolase family protein | -1.99871 | 2.69E-14 |  |  |  |
| Cs4g03140 | Xyloglucan endotransglucosylase/hydrolase family protein | -2.22126 | 4.89E-26 |  |  |  |
| Cs4g03200 | Xyloglucan endotransglucosylase/hydrolase family protein | -1.099646871 | 1.10E-06 |  |  |  |
| Cs4g03220 | Probable xyloglucan endotransglucosylase/hydrolase protein 23-like precursor | -2.032738542 | 3.84E-13 |  |  |  |
| Cs8g12020 | Xyloglucan endotransglycosylase | -1.061503389 | 0.041821 |  |  |  |
| Cs8g15720 | Xyloglucan endotransglycosylase 1 | -1.624640701 | 3.48E-12 |  |  |  |
| orange1.1t02385 | Xyloglucan endotransglycosylase 3 | -1.154207137 | 0.000123 |  |  |  |
| Cs9g06240 | Xyloglucan-specific endoglucanase inhibitor protein | -1.76343 | 6.29E-20 |  |  |  |
| Cs9g06180 | Xyloglucan-specific endoglucanase inhibitor protein | -2.06197 | 2.93E-09 |  |  |  |
| **Cs9g06230** | **Xyloglucan-specific endoglucanase inhibitor protein** | **-1.55926** | **3.59E-15** |  | **1.137022676** | **6.26E-56** |
| Cs5g26290 | Alpha-expansin 1 precursor | -1.13853376 | 1.19E-07 |  |  |  |
| Cs8g01880 | Expansin-like protein isoform 1 | -1.20458 | 8.76E-08 |  |  |  |
| orange1.1t00855 | Expansin B3, BETA 1.6 isoform 1 | -2.462304386 | 4.90E-12 |  |  |  |
| **XLOC_004133** | **Expansin-like B1, BETA 3.1,EXLB1** | **-2.576744237** | **1.48E-07** |  | **-1.615340676** | **4.07E-17** |
| Cs5g07852 | Expansin-like B1, BETA 3.1,EXLB1 |  |  |  | -1.32823 | 2.54E-12 |
| Cs5g07854 | Expansin-like B1, BETA 3.1,EXLB1 |  |  |  | -1.15673 | 1.57E-54 |
| Cs5g07856 | Expansin-like B1, BETA 3.1,EXLB1 |  |  |  | -1.51043 | 2.90E-47 |
| Cs7g22280 | Expansin-like B1 |  |  |  | -1.08417595 | 4.05E-18 |
| **Cs1g07100** | **Expansin-A12-like** | **-3.001995722** | **0.002122** |  | **1.458199861** | **5.21E-05** |
| Cs8g02110 | Expansin-like A2 |  |  |  | 2.669734201 | 3.88E-80 |
| orange1.1t00614 | Expansin-A31 |  |  |  | 1.587242156 | 0.000600 |
| Cs6g21460 | Expansin protein |  |  |  | 3.616389311 | 0.019771 |
|  |  |  |  |  |  |  |
| XLOC_011383 | Extensin-like protein | -1.369602774 | 2.38E-05 |  |  |  |
| Cs4g01020 | Leucine-rich repeat/extensin 2 | -1.125592009 | 5.07E-06 |  |  |  |
| Cs8g01960 | L-ascorbate oxidase homolog | -1.978108064 | 1.20E-14 |  | 1.390178172 | 3.04E-111 |
| Cs2g11900 | Peroxidase 7-like |  |  |  | 1.351073133 | 0.003408 |
| Cs3g02270 | Lignin-forming anionic peroxidase-like |  |  |  | 2.401960078 | 8.46E-05 |
| Cs3g02280 | Lignin-forming anionic peroxidase-like |  |  |  | 1.874601919 | 1.65E-06 |
| Cs3g02310 | Lignin-forming anionic peroxidase-like |  |  |  | 1.7868186 | 2.17E-31 |
| Cs3g26600 | Peroxidase 64-like isoform 1 |  |  |  | 1.12380635 | 1.04E-12 |
| Cs4g03740 | Peroxidase 45-like |  |  |  | 1.14351271 | 1.76E-45 |
| Cs6g09680 | Peroxidase superfamily protein |  |  |  | 1.102017114 | 1.33E-30 |
| Cs7g13530 | Peroxidase 51-like |  |  |  | 1.633145383 | 2.33E-05 |
| Cs7g20700 | Peroxidase N1 |  |  |  | 1.514079389 | 1.59E-35 |
| **Cs9g05130** | **Peroxidase superfamily protein** | **1.325344149** | **0.000638** |  | **1.352233162** | **2.12E-59** |
| **orange1.1t05402** | **Cationic peroxidase 1-like** | **2.018385382** | **4.96E-07** |  | **1.47147719** | **2.29E-31** |
| orange1.1t02033 | Peroxidase superfamily protein | 1.548796734 | 9.30E-18 |  |  |  |
| orange1.1t02038 | Peroxidase superfamily protein | 1.622027219 | 6.03E-05 |  |  |  |
| orange1.1t02225 | Peroxidase precursor | 1.366050443 | 6.43E-06 |  |  |  |
| Cs1g25930 | Peroxidase 64 | -2.183597275 | 0.001307 |  |  |  |
| Cs2g03110 | Secretory peroxidase | -1.063358765 | 2.37E-05 |  |  |  |
| Cs3g20770 | Peroxidase | -8.350742537 | 0.047100 |  |  |  |
| Cs9g16590 | Peroxidase 66-like | -1.116915267 | 0.004739 |  |  |  |
| Cs1g17550 | Germin-like protein |  |  |  | 1.5821396 | 1.03E-07 |
| Cs3g04870 | Germin-like protein subfamily T member 2-like |  |  |  | 3.421406709 | 1.97E-11 |
| Cs3g04880 | Germin-like protein subfamily T member 2 |  |  |  | 1.112215817 | 8.25E-11 |
| **Cs5g07750** | **Germin-like protein 9-3** | **-1.730381807** | **1.13E-09** |  | **-1.485274696** | **0.002502** |
| **Cs7g20270** | **Germin-like protein 9-3** | **-4.067189815** | **1.05E-23** |  | **-2.510835241** | **9.34E-09** |
| **XLOC_020823** | **Germin-like protein 9-3** | **-5.410815085** | **4.78E-76** |  | **-5.093280746** | **0** |
| **XLOC_021846** | **Germin-like protein 9-3** | **-2.151597185** | **5.32E-17** |  | **1.30712287** | **6.96E-17** |
| Cs5g25660 | Germin-like protein |  |  |  | 2.651747007 | 0.014349 |
| Cs5g14180 | Germin-like protein subfamily 1 member 14 | 2.139139926 | 2.75E-17 |  |  |  |
| Cs5g14210 | Germin-like protein subfamily 1 member 14 | 2.162281585 | 7.15E-19 |  |  |  |
| Cs5g14230 | Germin-like protein subfamily 1 member 14 | 1.437582719 | 1.66E-10 |  |  |  |
| Cs5g14290 | Germin-like protein subfamily 1 member 14 | 1.723789053 | 3.29E-05 |  |  |  |
| Cs5g14300 | Germin-like protein subfamily 1 member 14 | 2.991859602 | 7.35E-08 |  |  |  |
| Cs5g14330 | Germin-like protein subfamily 1 member 18 | 1.150844167 | 0.000221 |  |  |  |
| Cs5g25740 | Germin-like protein | 2.785272181 | 7.51E-05 |  |  |  |
| Cs5g25760 | Germin-like protein | 2.742205863 | 0.001300 |  |  |  |
| Cs5g25810 | Germin-like protein subfamily 1 member 14 | 1.106423771 | 3.60E-11 |  |  |  |
| **Cs8g01810** | **Acidic class II chitinase** | **-1.16071** | **4.01E-10** |  | **-1.29199889** | **2.47E-67** |
| **Cs8g17580** | **Chitinase 1** | **-3.500164416** | **9.71E-20** |  | **-2.379358601** | **4.14E-17** |
| XLOC_012032 | Chitinase | 3.811283209 | 1.57E-05 |  |  |  |
| **orange1.1t00435** | **Acidic endochitinase** | **-4.603713356** | **5.39E-48** |  | **-1.323302626** | **1.32E-34** |
| orange1.1t01780 | Class II chitinase | -1.257093911 | 3.80E-05 |  |  |  |
| **orange1.1t05124** | **Acidic class II chitinase** | **-1.395575138** | **1.34E-12** |  | **-1.26860658** | **1.37E-45** |
| **Cs1g05710** | **Chitinase 2-like** | **-10.22844414** | **0.002895** |  | **-2.086741982** | **0.000357** |
| Cs8g01840 | Chitinase |  |  |  | -2.40613 | 1.22E-124 |
| Cs8g01850 | Acidic class I chitinase |  |  |  | -1.31326 | 6.97E-69 |
| XLOC_009942 | Glycosyl hydrolase family protein with chitinase insertion domain | -1.368290734 | 1.76E-10 |  |  |  |
| Cs9g02570 | Arabinofuranosidase | 1.32181551 | 0.000533 |  |  |  |
| Cs3g27500 | Glucan endo-1,3-beta-glucosidase 13 | -1.703438985 | 6.51E-05 |  |  |  |
| Cs9g05770 | Endo-1,3;1,4-beta-D-glucanase-like | -1.70356 | 7.05E-11 |  |  |  |
| Cs9g05790 | Endo-1,3;1,4-beta-D-glucanase-like | -1.05596 | 0.006006 |  |  |  |
| orange1.1t00634 | Beta-1,3-glucanase | 4.544996 | 0.000959 |  |  |  |
| **orange1.1t00635** | **Beta-1,3-glucanase** | **2.549647** | **0.000254** |  | **-1.205896892** | **8.59E-05** |
| **orange1.1t00636** | **Beta-1,3-glucanase** | **2.475196** | **0.007405** |  | **-1.603935594** | **0.000114** |
| orange1.1t00643 | Beta-1,3-glucanase | -1.17375 | 3.58E-07 |  |  |  |
| orange1.1t00647 | Beta-1,3-glucanase | -1.17755 | 2.44E-08 |  |  |  |
| orange1.1t01658 | Beta-xylosidase 2 | -1.303614899 | 6.86E-06 |  |  |  |
| orange1.1t02294 | Polygalacturonase | -9.43661994 | 0.007779 |  |  |  |
| orange1.1t02296 | Polygalacturonase | -8.942823831 | 0.039131 |  |  |  |
| Cs7g01980 | Polygalacturonase-inhibiting protein | -1.520964777 | 2.16E-10 |  |  |  |
| orange1.1t05473 | Polygalacturonase QRT3-like |  |  |  | -1.476407322 | 0.031452 |
| Cs4g02450 | UDP-D-glucuronate 4-epimerase 1 | -1.928995331 | 1.83E-19 |  |  |  |
| orange1.1t01884 | UDP-D-glucuronate 4-epimerase 6 | -1.025954995 | 2.97E-06 |  |  |  |
| Cs7g03050 | UDP-glucose 6-dehydrogenase family protein | -1.4208177 | 4.38E-09 |  |  |  |
| **Cs7g05750** | **Glucuronidase 3 isoform 1** | **7.513061881** | **3.91E-125** |  | **5.613233553** | **7.09E-201** |
| Cs3g18050 | EXORDIUM like 2 | -1.339753788 | 1.87E-10 |  |  |  |
| orange1.1t02003 | EXORDIUM like 2 | -1.025809643 | 1.99E-07 |  |  |  |
| orange1.1t00255 | Fasciclin-like arabinogalactan protein 10-like | -1.243304651 | 1.79E-10 |  |  |  |
| Cs2g12170 | Beta-hexosaminidase 3 isoform 1 |  |  |  | 1.052921641 | 3.17E-32 |
| **Cs7g01060** | **Beta-hexosaminidase-like** | **-2.640800576** | **1.14E-26** |  | **-1.503885828** | **2.04E-24** |
| Cs4g03145 | Brassinosteroid-regulated protein BRU1 | -3.030886944 | 3.12E-06 |  |  |  |
| Cs8g18200 | Thaumatin-like protein | -1.095500916 | 1.83E-07 |  |  |  |
| Cs9g06210 | Basic 7S globulin-like | -2.63275 | 3.49E-23 |  |  |  |
| Cs9g06030 | Basic 7S globulin-like | -1.15383 | 0.029499 |  |  |  |
| **Cs9g06040** | **Basic 7S globulin-like** | **-2.65763** | **1.29E-16** |  | **1.005726193** | **4.60E-19** |
| **Cs9g06060** | **Basic 7S globulin-like** | **-2.21888** | **1.51E-11** |  | **1.20107504** | **5.92E-80** |
| **Cs9g06160** | **Basic 7S globulin-like** | **3.469115** | **1.40E-06** |  | **1.578600097** | **1.90E-11** |
| Cs9g06170 | Basic 7S globulin-like | -1.78621 | 6.60E-10 |  |  |  |
| **orange1.1t05612** | **Basic 7S globulin-like** | **-1.637904618** | **0.011652** |  | **1.230202053** | **1.92E-08** |
| Cs7g03525 | Cinnamyl alcohol dehydrogenase | 1.347541736 | 0.008060 |  |  |  |
| Cs4g04530 | Cinnamate 4-hydroxylase CYP73 |  |  |  | 1.149636145 | 7.25E-15 |
| Cs9g16630 | Peptidoglycan-binding LysM domain-containing protein |  |  |  | -1.258894938 | 7.47E-23 |
| Cs8g17630 | Laccase-17 |  |  |  | 1.403730978 | 1.33E-23 |
| Cs8g20850 | Laccase 3 |  |  |  | 1.255566649 | 5.86E-47 |
| Cs4g07350 | Subtilase family protein | -1.292598482 | 2.64E-09 |  |  |  |
| Cs3g15800 | Subtilisin-like protease-like |  |  |  | -1.191982799 | 2.78E-104 |
| Cs8g08590 | Subtilisin-like protease-like |  |  |  | -1.000829235 | 1.26E-09 |
| Cs8g08580 | Subtilisin-like protease-like | 1.006866688 | 0.012901 |  |  |  |
| **Cs9g06190** | **Eukaryotic aspartyl protease family protein isoform 1** | **-3.388279351** | **7.03E-28** |  | **1.21892** | **7.99E-30** |
| **Cs9g06200** | **Eukaryotic aspartyl protease family protein isoform 1** | **-2.659712914** | **4.25E-09** |  | **2.586757** | **3.66E-49** |
| Cs1g16180 | Eukaryotic aspartyl protease family protein | -1.01972577 | 0.000154 |  |  |  |
| Cs5g08910 | Eukaryotic aspartyl protease family protein | -1.115936934 | 9.59E-09 |  |  |  |
| XLOC_019281 | Eukaryotic aspartyl protease family protein isoform 2 | -1.164712228 | 1.79E-07 |  |  |  |
| orange1.1t05508 | COBRA-like extracellular glycosyl-phosphatidyl inositol-anchored protein family isoform 1 | -1.399337752 | 0.001450 |  |  |  |
| Cs8g15710 | Exostosin family protein | -1.129218783 | 1.76E-08 |  |  |  |
|  |  |  |  |  |  |  |
| Cytoskeleton metabolisom | |  |  |  |  |  |
| Cs5g03620 | Beta-tubulin 10 | -1.190825709 | 1.45E-05 |  |  |  |
| Cs8g03680 | Alpha tubulin | -1.221058631 | 1.71E-08 |  |  |  |
| Cs5g12860 | ATP binding microtubule motor family protein isoform 1 | -1.031154373 | 0.026980 |  |  |  |
| Cs4g12990 | Formin-like protein 18 | -1.477441405 | 5.29E-06 |  |  |  |
| Cs8g09980 | Microtubule associated protein family protein isoform 4 | 1.300798119 | 0.029954 |  |  |  |
| Cs1g15130 | Dynein light chain LC6, flagellar outer arm-like | 2.020784967 | 9.76E-07 |  |  |  |
| orange1.1t03723 | Dynein light chain type 1 family protein |  |  |  | 1.179074253 | 0.032978 |
| Cs8g08430 | Kinesin-related protein 11-like isoform X1 |  |  |  | 1.094580912 | 2.76E-29 |
| Cs9g17560 | Kinesin-like protein KIF22-like |  |  |  | 2.144146801 | 0.001343 |
| Cs5g06940 | Kinesin-like protein |  |  |  | 1.003732964 | 0.015395 |
| Cs8g14100 | Kinesin-like protein |  |  |  | 1.241914333 | 3.75E-05 |
| Cs3g14860 | Kinesin-4-like |  |  |  | 1.235081239 | 1.11E-07 |
| Cs9g01520 | Phragmoplast orienting kinesin 1 |  |  |  | 1.209648005 | 1.31E-14 |
| Cs3g17000 | Di-glucose binding protein with Kinesin motor domain |  |  |  | 1.114197144 | 0.000918 |
| orange1.1t03949 | Centromere-associated protein E-like |  |  |  | 1.525674421 | 0.031212 |
| orange1.1t03950 | Centromere-associated protein E-like |  |  |  | 1.035578348 | 0.019848 |
| Cs6g22060 | TPX2 (targeting protein for Xklp2) family protein |  |  |  | 1.011353943 | 1.75E-08 |
| Cs5g05840 | Cell division cycle protein 48 homolog |  |  |  | -1.269136026 | 1.00E-66 |

*DEGs shared by the two citrus species were highlighted in bold. FC, fold change; FDR, false discovery rate.*

**TABLE S10 | DEGs related to carbohydrate and energy metabolism in Al-treated *C. sinensis* and *C. grandis* roots.**

| **ID** | **Description** | ***C. grandis*** | |  | ***C. sinensis*** | |  |
| --- | --- | --- | --- | --- | --- | --- | --- |
|  |  | **log2 (FC)** | **FDR** |  | **log2 (FC)** | **FDR** |  |
| Cs2g03260 | Pyruvate dehydrogenase E1 component subunit beta | -1.616617135 | 7.62E-15 |  |  |  |  |
| Cs8g06950 | Pyruvate dehydrogenase E1 component subunit beta-like isoform 2 | -1.547569726 | 4.88E-13 |  |  |  |  |
| orange1.1t05835 | Pyruvate dehydrogenase E1 component subunit alpha | -1.057171117 | 2.00E-07 |  |  |  |  |
| Cs3g22480 | Dihydrolipoyllysine-residue acetyltransferase component of pyruvate dehydrogenase complex, mitochondrial-like isoform 1 | -1.204769583 | 2.30E-09 |  |  |  |  |
| Cs2g13410 | Probable fructose-bisphosphate aldolase 3, chloroplastic | -1.047198284 | 3.54E-07 |  |  |  |  |
| **Cs5g30520** | **Pyruvate kinase isozyme A, chloroplastic isoform 1** | **-1.854935055** | **0.007858** |  | **1.11623874** | **6.43E-12** |  |
| Cs4g08150 | Aldolase superfamily protein | 2.754116733 | 0.011421 |  |  |  |  |
| **Cs4g01490** | **NADP-dependent glyceraldehyde-3-phosphate dehydrogenase-like** | **2.845271187** | **2.80E-13** |  | **1.23105656** | **2.06E-05** |  |
| Cs1g15970 | 6-phosphofructokinase 3 |  |  |  | 1.223502829 | 4.48E-13 |  |
| Cs4g13070 | 6-phosphofructokinase 3 |  |  |  | 1.134091069 | 1.62E-85 |  |
| Cs3g18540 | Phosphoglycerate mutase family protein isoform 2, partial |  |  |  | 1.750853556 | 1.57E-65 |  |
| Cs6g08840 | Thiamine pyrophosphate dependent pyruvate decarboxylase family protein |  |  |  | 1.263246445 | 1.40E-85 |  |
| Cs3g16700 | Phosphoenolpyruvate carboxykinase [ATP]-like |  |  |  | -1.076974408 | 1.64E-39 |  |
| Cs3g20800 | Alcohol dehydrogenase |  |  |  | 2.313098306 | 5.29E-218 |  |
| Cs3g20810 | Alcohol dehydrogenase |  |  |  | 1.832569477 | 2.25E-41 |  |
| orange1.1t04627 | Alcohol dehydrogenase class-3 |  |  |  | -1.2311327 | 2.24E-05 |  |
| Cs2g30190 | Aldehyde dehydrogenase family 3 member F1 |  |  |  | -2.427091434 | 0.003446 |  |
| Cs4g16680 | Aldehyde dehydrogenase family 2 member C4-like |  |  |  | -1.876501972 | 0.012321 |  |
| Cs2g14940 | Glyceraldehyde-3-phosphate dehydrogenase | 3.856383691 | 1.50E-09 |  |  |  |  |
| Cs3g27520 | Glyceraldehyde-3-phosphate dehydrogenase B subunit | -1.814343002 | 0.039425 |  |  |  |  |
| Cs3g19080 | 6-phosphogluconolactonase | -1.301900229 | 0.000965 |  |  |  |  |
| XLOC_015721 | Glucose-6-phosphate dehydrogenase, partial | 1.310235566 | 0.006039 |  |  |  |  |
| Cs5g19060 | Sucrose-phosphate synthase family protein isoform 1 | 1.423814801 | 8.16E-12 |  |  |  |  |
| Cs7g05690 | Sucrose phosphate synthase 1F | 1.66821658 | 3.46E-18 |  |  |  |  |
| Cs9g03980 | Sucrose synthase 2 isoform 1 |  |  |  | 1.039309519 | 3.03E-08 |  |
| Cs3g13670 | Glycogenin-1 | 1.114213816 | 0.000234 |  |  |  |  |
| Cs8g07230 | ADP-glucose pyrophosphorylase large subunit | 1.186839095 | 1.57E-13 |  |  |  |  |
| **Cs2g06700** | **Beta-galactosidase 8** | **-2.605514483** | **6.86E-16** |  | **-2.102373091** | **5.17E-17** |  |
| Cs3g25410 | Beta-galactosidase BG1-like precursor | -1.418426452 | 5.36E-12 |  |  |  |  |
| Cs5g17540 | Beta-galactosidase isoform 1 | -2.028899816 | 0.004149 |  |  |  |  |
| Cs5g17230 | Alpha-galactosidase-like | -2.141482447 | 5.30E-21 |  |  |  |  |
| Cs4g05490 | Raffinose synthase family protein isoform 1 | -1.414667274 | 2.78E-10 |  |  |  |  |
| Cs5g05900 | Raffinose synthase family protein |  |  |  | -1.741541637 | 2.97E-12 |  |
| Cs5g26450 | O-Glycosyl hydrolases family 17 protein | -1.275107825 | 4.69E-08 |  |  |  |  |
| Cs8g18530 | O-Glycosyl hydrolases family 17 protein isoform 1 | -1.077933861 | 4.12E-08 |  |  |  |  |
| orange1.1t01756 | O-Glycosyl hydrolases family 17 protein | -3.610071406 | 2.64E-11 |  |  |  |  |
| Cs2g10900 | Glycosyl hydrolases family 31 protein isoform 1 | -2.178383306 | 5.68E-19 |  |  |  |  |
| Cs8g08680 | Glycosyl hydrolase family protein | -1.014006877 | 0.002094 |  |  |  |  |
| Cs1g18220 | Glycosyl hydrolases family 32 protein isoform 1 |  |  |  | 1.372476574 | 1.26E-96 |  |
| XLOC_022884 | ATP synthase subunit alpha | 1.579738821 | 0.003804 |  |  |  |  |
| orange1.1t01461 | NADH dehydrogenase [ubiquinone] 1 alpha subcomplex subunit 13-B-like | -1.131899625 | 0.044498 |  |  |  |  |
| Cs2g06650 | Alternative NAD(P)H dehydrogenase 1 |  |  |  | 2.012524189 | 8.32E-270 |  |
| Cs4g02730 | Trehalose-6-phosphate synthase |  |  |  | 1.103228701 | 6.98E-23 |  |
| Cs4g19200 | NADP-malic enzyme 4 |  |  |  | 1.279479897 | 1.59E-125 |  |
| orange1.1t06022 | Sorbitol dehydrogenase-like protein |  |  |  | 3.854399698 | 0.007157 |  |
| Cs3g25730 | Galactose oxidase |  |  |  | 1.068639171 | 2.73E-26 |  |
| Cs5g02660 | Galactose mutarotase-like superfamily protein |  |  |  | -1.253595432 | 6.99E-06 |  |
| Cs3g15460 | Galactinol synthase 3 |  |  |  | 1.274375046 | 3.73E-44 |  |
| orange1.1t01729 | Galactinol synthase 6 |  |  |  | 1.914533016 | 3.07E-05 |  |
| Cs5g16880 | EG |  |  |  | 10.98692284 | 0.000335 |  |
| Cs5g06930 | Malate synthase, glyoxysomal-like |  |  |  | -1.358195477 | 0.001866 |  |
| Cs7g01370 | Vicianin hydrolase-like |  |  |  | -1.034013742 | 6.00E-14 |  |
| Cs9g16680 | L-idonate 5-dehydrogenase |  |  |  | -1.234649867 | 4.82E-57 |  |

*DEGs shared by the two citrus species were highlighted in bold. FC, fold change; FDR, false discovery rate.*

**TABLE S11 | DEGs involved in protein and amino acid metabolism in Al-treated *C. sinensis* and *C. grandis* roots.**

| **ID** | **Description** | ***C. grandis*** | |  | ***C. sinensis*** | |  |
| --- | --- | --- | --- | --- | --- | --- | --- |
|  |  | **log2 (FC)** | **FDR** |  | **log2 (FC)** | **FDR** |  |
| Ubiquitin proteosome pathway | |  |  |  |  |  |  |
| Cs5g29920 | RING/U-box superfamily protein |  |  |  | 1.597336045 | 6.15E-05 |  |
| Cs4g01190 | RING/U-box superfamily protein |  |  |  | -1.008378551 | 0.000726 |  |
| Cs2g30000 | RING/U-box superfamily protein isoform 1 | 1.187030618 | 5.38E-11 |  |  |  |  |
| Cs4g20130 | RING finger and CHY zinc finger domain-containing protein 1-like |  |  |  | -1.486532455 | 4.31E-92 |  |
| Cs1g05020 | RING zinc finger protein | 1.879539237 | 0.002364 |  |  |  |  |
| Cs2g04430 | Plant U-box 17 | -1.218888247 | 2.18E-08 |  |  |  |  |
| Cs6g13640 | Plant U-box 23 | -3.259944164 | 7.90E-57 |  |  |  |  |
| Cs3g21130 | U-box domain-containing protein 29-like | -1.61367531 | 7.71E-15 |  |  |  |  |
| Cs8g01550 | U-box domain-containing protein 15 isoform 1 | -1.175845823 | 0.008329 |  |  |  |  |
| Cs5g05260 | U-box domain-containing protein 19-like | -1.477047143 | 1.64E-10 |  |  |  |  |
| Cs6g09780 | U-box domain-containing protein 16-like | -1.093473446 | 7.25E-08 |  |  |  |  |
| orange1.1t00371 | U-box domain-containing protein 33-like | 1.437566783 | 7.57E-14 |  |  |  |  |
| Cs2g01590 | U-box domain-containing protein 33 | 1.855942926 | 7.77E-15 |  |  |  |  |
| Cs4g04570 | U-box domain-containing protein 35-like | 1.565452778 | 0.033499 |  |  |  |  |
| Cs3g13560 | E3 ubiquitin-protein ligase Topors-like | 1.408114064 | 1.408114 |  |  |  |  |
| orange1.1t03837 | E3 ubiquitin-protein ligase ATL42 | 2.237844939 | 5.73E-19 |  |  |  |  |
| Cs5g29440 | E3 ubiquitin-protein ligase BAH1 |  |  |  | -1.265067567 | 5.36E-06 |  |
| orange1.1t05194 | E3 ubiquitin-protein ligase |  |  |  | -1.508675061 | 4.15E-09 |  |
| Cs9g02150 | Probable ubiquitin-conjugating enzyme E2 23-like | 1.448038407 | 0.005278 |  |  |  |  |
| Cs2g06030 | Probable ubiquitin-conjugating enzyme E2 24-like |  |  |  | -1.423475952 | 4.73E-69 |  |
| Cs2g30950 | Ubiquitin-conjugating enzyme E2 19-like |  |  |  | 1.020165672 | 4.13E-21 |  |
| orange1.1t05710 | Polyubiquitin | -1.034206813 | 0.000203 |  |  |  |  |
| orange1.1t05568 | Polyubiquitin |  |  |  | 14.49507115 | 6.33E-06 |  |
| Cs4g06500 | F-box family protein isoform 1 |  |  |  | -1.437279707 | 3.98E-08 |  |
| XLOC_005464 | F-box family protein |  |  |  | 2.102484344 | 0.003452 |  |
| Cs1g23680 | RUB1 conjugating enzyme 1 isoform 2 | 1.383036612 | 0.001515 |  |  |  |  |
| Cs4g05010 | OS-9 | 1.169371705 | 6.43E-12 |  |  |  |  |
| orange1.1t00374 | SKP1-like protein 21 isoform 2 | 1.014849995 | 0.029980 |  |  |  |  |
| Cs2g11740 | 20S proteasome alpha subunit C1 |  |  |  | 1.435330785 | 9.90E-05 |  |
| Cs1g24230 | Bg55 | 1.123929653 | 1.33E-07 |  |  |  |  |
|  |  |  |  |  |  |  |  |
| Proteases (also termed proteinases and peptidases) and inhibitors | | |  |  |  |  |  |
| **Cs9g06190** | **Eukaryotic aspartyl protease family protein isoform 1** | **-3.388279351** | **7.03E-28** |  | **1.21892** | **7.99E-30** |  |
| **Cs9g06200** | **Eukaryotic aspartyl protease family protein isoform 1** | **-2.659712914** | **4.25E-09** |  | **2.586757** | **3.66E-49** |  |
| Cs1g16180 | Eukaryotic aspartyl protease family protein | -1.01972577 | 0.000154 |  |  |  |  |
| Cs5g08910 | Eukaryotic aspartyl protease family protein | -1.115936934 | 9.59E-09 |  |  |  |  |
| XLOC_019281 | Eukaryotic aspartyl protease family protein isoform 2 | -1.164712228 | 1.79E-07 |  |  |  |  |
| Cs2g10830 | Aspartyl protease family protein |  |  |  | 7.966153567 | 0.043442 |  |
| orange1.1t00502 | Protein ASPARTIC PROTEASE IN GUARD CELL 1-like | -1.060079382 | 2.08E-08 |  |  |  |  |
| Cs7g13360 | Aspartic proteinase nepenthesin-1-like | -1.250107438 | 9.36E-11 |  |  |  |  |
| Cs7g15810 | Aspartic proteinase nepenthesin-1 | 1.702385922 | 0.008807 |  |  |  |  |
| Cs2g02620 | Subtilisin-like protease SDD1 | -3.108990934 | 0.005911 |  |  |  |  |
| Cs3g06710 | Subtilisin-like protease-like | -1.019974809 | 0.002856 |  |  |  |  |
| Cs8g08580 | Subtilisin-like protease-like | 1.006866688 | 0.012901 |  |  |  |  |
| **Cs3g23350** | **Subtilisin-like protease-like** | **-4.548834963** | **9.50E-82** |  | **-1.814890262** | **9.49E-67** |  |
| **Cs3g23360** | **Subtilisin-like protease** | **-4.521743845** | **1.18E-89** |  | **-2.388269039** | **1.90E-231** |  |
| **Cs8g05500** | **Subtilisin-like protease-like** | **-2.20152008** | **6.40E-12** |  | **-1.490290861** | **7.32E-06** |  |
| **Cs8g06080** | **Subtilisin-like protease-like** | **-1.980605852** | **1.91E-15** |  | **-1.362807258** | **3.06E-97** |  |
| orange1.1t00282 | Subtilisin-like protease-like |  |  |  | 1.235051965 | 3.82E-19 |  |
| Cs3g26520 | Subtilisin-like serine endopeptidase family protein isoform 1 |  |  |  | -1.487590012 | 4.87E-12 |  |
| Cs3g15800 | Subtilisin-like protease-like |  |  |  | -1.191982799 | 2.78E-104 |  |
| Cs8g08590 | Subtilisin-like protease-like |  |  |  | -1.000829235 | 1.26E-09 |  |
| **Cs8g02980** | **Subtilase family protein** | **-5.326719981** | **1.85E-55** |  | **-2.872976772** | **1.22E-15** |  |
| Cs4g07350 | Subtilase family protein | -1.292598482 | 2.64E-09 |  |  |  |  |
| Cs1g16670 | Subtilase family protein isoform 1 | -1.068078233 | 2.80E-07 |  |  |  |  |
| Cs9g06210 | Basic 7S globulin-like | -2.63275 | 3.49E-23 |  |  |  |  |
| Cs9g06030 | Basic 7S globulin-like | -1.15383 | 0.029499 |  |  |  |  |
| **Cs9g06040** | **Basic 7S globulin-like** | **-2.65763** | **1.29E-16** |  | **1.005726193** | **4.60E-19** |  |
| **Cs9g06060** | **Basic 7S globulin-like** | **-2.21888** | **1.51E-11** |  | **1.20107504** | **5.92E-80** |  |
| **Cs9g06160** | **Basic 7S globulin-like** | **3.469115** | **1.40E-06** |  | **1.578600097** | **1.90E-11** |  |
| Cs9g06170 | Basic 7S globulin-like | -1.78621 | 6.60E-10 |  |  |  |  |
| **orange1.1t05612** | **Basic 7S globulin-like** | **-1.637904618** | **0.011652** |  | **1.230202053** | **1.92E-08** |  |
| **Cs2g04940** | **Senescence-associated gene 12** | **-4.446725564** | **5.18E-28** |  | **-2.074053173** | **3.08E-68** |  |
| **Cs2g15480** | **Senescence-associated gene 12** | **-4.478214178** | **1.52E-29** |  | **-1.558286993** | **3.40E-64** |  |
| **Cs2g15490** | **Senescence-associated gene 12** | **-4.317663587** | **6.83E-26** |  | **-1.517321036** | **2.29E-93** |  |
| **Cs2g15700** | **Senescence-associated gene 12** | **-4.508086816** | **2.21E-46** |  | **-1.869758909** | **1.27E-80** |  |
| orange1.1t05706 | Senescence-associated gene 12 | -4.899568836 | 8.50E-49 |  |  |  |  |
| Cs8g03860 | Serine carboxypeptidase-like 18-like | 3.210324337 | 3.87E-35 |  |  |  |  |
| XLOC_004154 | Serine carboxypeptidase II-3-like | 1.573047246 | 0.018045 |  |  |  |  |
| orange1.1t04346 | Serine carboxypeptidase S28 family protein | -2.55056087 | 0.012827 |  |  |  |  |
| **XLOC_015629** | **Serine carboxypeptidase-like 46-like** | **-3.470926158** | **1.27E-57** |  | **-2.321738835** | **9.29E-59** |  |
| Cs2g06490 | Serine carboxypeptidase-like 31-like |  |  |  | -2.441116843 | 6.08E-26 |  |
| Cs3g16220 | Serine carboxypeptidase-like 18-like |  |  |  | -1.448455469 | 4.31E-06 |  |
| orange1.1t03718 | Cysteine proteinase | 11.53246137 | 4.90E-05 |  |  |  |  |
| orange1.1t05099 | Cysteine proteinase | 11.72453986 | 2.74E-05 |  |  |  |  |
| orange1.1t05314 | Cysteine proteinase | 10.56700014 | 0.001001 |  |  |  |  |
| Cs8g01170 | Xylem cysteine proteinase 1-like | -1.506701302 | 6.54E-15 |  |  |  |  |
| **Cs2g04950** | **KDEL-tailed cysteine endopeptidase CEP1-like** | **-4.102129613** | **2.67E-28** |  | **-2.800173756** | **3.67E-26** |  |
| Cs9g02840 | KDEL-tailed cysteine endopeptidase CEP1-like | -9.398097006 | 0.002364 |  |  |  |  |
| Cs1g17850 | Puromycin-sensitive aminopeptidase-like | 1.184760111 | 4.69E-09 |  |  |  |  |
| Cs1g23280 | Protease 2-like | 1.049676509 | 0.000477 |  |  |  |  |
| orange1.1t05346 | Metalloendoproteinase 1-like |  |  |  | 1.44686929 | 5.92E-13 |  |
| Cs6g20450 | ATP-dependent zinc metalloprotease FtsH-like | 1.521940618 | 1.25E-08 |  |  |  |  |
| Cs9g16770 | ATP-dependent Clp protease ATP-binding subunit clpX | 3.962671547 | 1.03E-13 |  |  |  |  |
| Cs8g12960 | GPI-anchor transamidase-like isoform X1 |  |  |  | 1.231436637 | 0.020474 |  |
| **Cs5g13890** | **Trypsin inhibitor** | **-3.272131863** | **8.10E-26** |  | **-4.970470272** | **2.96E-267** |  |
| **Cs5g13960** | **Trypsin inhibitor** | **-10.54042837** | **0.010316** |  | **-4.681119033** | **3.11E-31** |  |
| **Cs9g08680** | **Trypsin inhibitor** | **-2.376975278** | **2.46E-20** |  | **-2.375585354** | **4.13E-08** |  |
| Cs9g08650 | Inhibitor of trypsin and hageman factor | -3.423073679 | 0.018815 |  |  |  |  |
| Cs9g08660 | Serine protease inhibitor |  |  |  | -1.173817793 | 0.010402 |  |
| Cs9g08670 | Serine protease inhibitor |  |  |  | -1.765681234 | 6.88E-31 |  |
| **Cs5g16770** | **Miraculin-like protein 2** | **4.785773527** | **1.73E-22** |  | **3.112296677** | **3.49E-14** |  |
| **orange1.1t05560** | **Type I proteinase inhibitor-like protein** | **-1.5332147** | **1.12E-06** |  | **-2.835895447** | **5.35E-110** |  |
| Cs4g14780 | Cystatin-like protein |  |  |  | 1.123511391 | 0.001537 |  |
| Cs8g19350 | Proteasome inhibitor-related | 1.641521983 | 1.92E-07 |  |  |  |  |
| Cs6g11570 | Protease inhibitor/seed storage/LTP family protein | -1.742490696 | 2.64E-19 |  |  |  |  |
| orange1.1t04805 | Kunitz family trypsin and protease inhibitor protein |  |  |  | 2.913178779 | 4.94E-08 |  |
|  |  |  |  |  |  |  |  |
| Protein biosynthesis | |  |  |  |  |  |  |
| **Cs4g06460** | **Eukaryotic initiation factor 4F subunit p150 isoform 1** | **-3.351926854** | **9.71E-47** |  | **-1.881125444** | **1.95E-39** |  |
| Cs4g05590 | Eukaryotic initiation factor 4A-15-like isoform 1 |  |  |  | 1.023827294 | 1.26E-05 |  |
| Cs2g01750 | Translation initiation factor IF-2-like |  |  |  | -1.316248251 | 6.34E-13 |  |
| Cs5g13870 | Ribosomal protein L14 | -1.926253542 | 0.015992 |  |  |  |  |
| Cs7g15650 | 50S ribosomal protein L14-like | 1.998789417 | 0.011652 |  |  |  |  |
| Cs3g21570 | 60S ribosomal protein L3 |  |  |  | 1.135152367 | 0.000162 |  |
| Cs6g21070 | 40S ribosomal protein S9-2-like |  |  |  | 2.776569098 | 0.036839 |  |
| Cs5g32630 | Peptide chain release factor 1 | 1.053595214 | 0.004957 |  |  |  |  |
|  |  |  |  |  |  |  |  |
| Protein folding | |  |  |  |  |  |  |
| **orange1.1t05332** | **Protein disulfide isomerase-like 1-4-like isoform X2** | **-2.541301606** | **2.08E-08** |  | **-1.87339106** | **7.02E-05** |  |
| orange1.1t03984 | Protein disulfide isomerase-like 1-3-like isoform 1 | -2.906831843 | 0.015345 |  |  |  |  |
| orange1.1t05377 | Peptidyl-prolyl cis-trans isomerase | 15.11534242 | 0.045716 |  |  |  |  |
|  |  |  |  |  |  |  |  |
| Heat shock proteins/chaperones | |  |  |  |  |  |  |
| Cs5g18500 | ATHSP22.0 |  |  |  | -1.092131211 | 0.021259 |  |
| Cs8g19520 | HSP20-like chaperones superfamily protein |  |  |  | 1.133014825 | 2.05E-06 |  |
| Cs1g16820 | Chaperone protein dnaJ |  |  |  | -1.01726224 | 2.45E-32 |  |
| Cs1g24490 | Probable mitochondrial chaperone bcs1-like |  |  |  | 1.280544039 | 7.73E-17 |  |
| XLOC_017339 | Chaperone protein dnaJ-related | -1.303143522 | 3.21E-08 |  |  |  |  |
| Cs7g07590 | Chaperone protein dnaJ-related protein | 1.439041277 | 3.99E-07 |  |  |  |  |
| orange1.1t02054 | DnaJ/Hsp40 cysteine-rich domain superfamily protein | 1.105736287 | 3.31E-06 |  |  |  |  |
| Cs4g06060 | DNAJ heat shock N-terminal domain-containing protein | 1.826581337 | 0.000234 |  |  |  |  |
| orange1.1t05694 | HSP20-like chaperones superfamily protein | 1.047711859 | 0.027142 |  |  |  |  |
| Cs9g06800 | Heat-shock protein 70T-2 | -1.195997286 | 3.16E-05 |  |  |  |  |
| Cs5g26370 | Heat shock protein 60-3A isoform 3 | 1.144259589 | 0.001679 |  |  |  |  |
| Cs5g03150 | Heat shock protein 83-like | 1.42898148 | 4.51E-07 |  |  |  |  |
| Cs7g06350 | Calmodulin-binding heat shock protein | 2.934777727 | 0.010453 |  |  |  |  |
| Cs6g13450 | Iron-sulfur cluster co-chaperone protein HscB, mitochondrial-like | 1.250437251 | 6.03E-05 |  |  |  |  |
|  |  |  |  |  |  |  |  |
| Amino acid metabolism | |  |  |  |  |  |  |
| Cs3g03620 | Aldolase-type TIM barrel family protein | 3.021025062 | 6.94E-19 |  |  |  |  |
| orange1.1t01400 | Prephenate dehydrogenase family protein | -1.060514666 | 4.16E-06 |  |  |  |  |
| Cs3g07770 | Arogenate/prephenate dehydratase | -2.025066403 | 1.47E-26 |  |  |  |  |
| Cs3g16430 | Branched-chain-amino-acid aminotransferase | -1.124021623 | 1.82E-05 |  |  |  |  |
| Cs3g16440 | Branched-chain-amino-acid aminotransferase | -1.473272604 | 0.028351 |  |  |  |  |
| Cs8g03420 | D-aminoacid aminotransferase-like PLP-dependent enzymes superfamily protein isoform 1 | -1.966288001 | 7.75E-07 |  |  |  |  |
| Cs2g31250 | Serine-glyoxylate aminotransferase-like | -2.591834882 | 2.00E-14 |  |  |  |  |
| **Cs4g05200** | **Arogenate dehydrogenase** | **-1.713754348** | **1.91E-05** |  | **1.537818717** | **7.57E-140** |  |
| Cs3g25080 | Ornithine carbamoyltransferase, chloroplastic | -1.005394304 | 1.02E-05 |  |  |  |  |
| orange1.1t03535 | Ornithine decarboxylase | -1.699352767 | 0.041105 |  |  |  |  |
| Cs8g12640 | Threonine aldolase 1 isoform 1 | 1.416904015 | 1.47E-10 |  |  |  |  |
| Cs8g20410 | Asparagine synthetase | -1.134957251 | 4.11E-08 |  |  |  |  |
| Cs6g09690 | Aspartate aminotransferase, mitochondrial-like |  |  |  | 2.697644326 | 6.71E-12 |  |
| Cs7g06000 | Aspartate aminotransferase, chloroplastic |  |  |  | -1.147176113 | 1.11E-89 |  |
| Cs9g05680 | Glutamine synthetase 2 | -1.812197633 | 2.40E-20 |  |  |  |  |
| Cs4g02230 | Class I glutamine amidotransferase-like superfamily protein | 2.247823272 | 0.009319 |  |  |  |  |
| Cs8g12450 | Class I glutamine amidotransferase-like superfamily protein | -1.225231937 | 4.36E-09 |  |  |  |  |
| Cs4g02260 | S-adenosylmethionine decarboxylase proenzyme-like | -1.271311845 | 3.86E-09 |  |  |  |  |
| Cs9g01410 | S-adenosylmethionine synthetase | -1.201982333 | 2.23E-08 |  |  |  |  |
| Cs7g13320 | Homocysteine S-methyltransferase 1 | -1.102982387 | 0.037034 |  |  |  |  |
| **Cs9g07420** | **Homocysteine s-methyltransferase** | **-1.104352292** | **7.98E-07** |  | **-1.223863343** | **6.89E-40** |  |
| **Cs3g12230** | **Bifunctional 3-dehydroquinate dehydratase/shikimate dehydrogenase, chloroplastic** | **1.125177066** | **1.46E-08** |  | **1.116582435** | **1.94E-39** |  |
| Cs9g17990 | NADH-dependent glutamate synthase 1 isoform 1 | -1.371926726 | 1.44E-08 |  |  |  |  |
| Cs5g06000 | Copper amine oxidase family protein isoform 1 | -1.01756098 | 1.25E-05 |  |  |  |  |
| Cs2g30610 | Tyrosine decarboxylase | -2.02292782 | 0.022754 |  | 1.474105355 | 1.74E-90 |  |
| Cs3g22910 | Tyrosine decarboxylase | -1.820882027 | 0.021207 |  | 2.018724235 | 8.20E-228 |  |
| Cs5g05650 | Tryptophan biosynthesis 1 |  |  |  | 1.149757918 | 2.55E-56 |  |
| Cs3g20490 | Thiamin diphosphate-binding fold superfamily protein |  |  |  | -1.019237116 | 8.77E-35 |  |
| **Cs5g35310** | **Chlorsulfuron/imidazolinone resistant 1** | **-4.72075416** | **4.87E-21** |  | **-2.312390557** | **5.31E-14** |  |
| Cs6g11950 | Phenylalanine-ammonia lyase |  |  |  | 1.352644541 | 4.16E-123 |  |
| Cs3g18540 | Phosphoglycerate mutase family protein isoform 2, partial |  |  |  | 1.750853556 | 1.57E-65 |  |

*DEGs shared by the two citrus species were highlighted in bold. FC, fold change; FDR, false discovery rate.*

**TABLE S12 | DEGs involved in lipid metabolism in Al-treated *C. sinensis* and *C. grandis* roots.**

| **ID** | **Description** | ***C. grandis*** | |  | ***C. sinensis*** | |
| --- | --- | --- | --- | --- | --- | --- |
|  |  | **log2 (FC)** | **FDR** |  | **log2 (FC)** | **FDR** |
| Jasmonic acid metabolism | |  |  |  |  |  |
| Cs1g17380 | Lipoxygenase 3 | -2.305185545 | 3.04E-18 |  |  |  |
| Cs3g13930 | Lipoxygenase | 1.540820244 | 7.97E-08 |  |  |  |
| orange1.1t03775 | Lipoxygenase | 1.669502344 | 6.03E-05 |  |  |  |
| Cs3g24230 | Allene oxide synthase | -1.709779915 | 2.53E-18 |  |  |  |
| XLOC_026240 | Lipoxygenase LOX1 |  |  |  | 1.749450072 | 1.31E-06 |
| Cs5g28310 | Allene oxide synthase, chloroplastic-like |  |  |  | 3.529515737 | 4.36E-08 |
| Cs3g27010 | OPC-8:0 CoA ligase1 isoform 1 |  |  |  | 1.311982222 | 2.23E-110 |
| Cs5g17920 | 12-oxophytodienoate reductase 2 |  |  |  | 1.302358865 | 1.69E-28 |
|  |  |  |  |  |  |  |
| **Lipid degradation** | |  |  |  |  |  |
| Cs1g17700 | GDSL esterase/lipase 1-like | -3.967255546 | 6.41E-05 |  |  |  |
| orange1.1t00452 | GDSL esterase/lipase At4g01130-like | -1.338018592 | 9.41E-05 |  |  |  |
| **Cs4g06810** | **GDSL esterase/lipase 5** | **-2.276727809** | **1.42E-24** |  | **-2.076809809** | **1.47E-42** |
| **Cs8g01950** | **GDSL esterase/lipase At5g33370-like** | **-2.781895029** | **8.45E-37** |  | **-2.307341515** | **3.82E-110** |
| Cs1g16710 | GDSL-motif lipase 5 |  |  |  | 1.256846016 | 0.000190 |
| Cs2g05250 | GDSL esterase/lipase At5g45950-like isoform 2 |  |  |  | 1.748091026 | 0.000228 |
| Cs3g19630 | GDSL-like Lipase/Acylhydrolase superfamily protein |  |  |  | 1.236186464 | 3.80E-09 |
| Cs5g17950 | GDSL esterase/lipase At1g71691-like |  |  |  | 2.058841564 | 1.81E-14 |
| **Cs1g22570** | **Phospholipase A1-IIbeta-like** | **-1.776294989** | **2.38E-11** |  | **1.285291594** | **1.71E-71** |
| Cs2g11280 | Phospholipase A 2A, IIA,PLA2A |  |  |  | 1.169319514 | 0.026140 |
| Cs7g14760 | Phospholipase C 2 | 1.231436744 | 1.20E-09 |  |  |  |
| Cs8g20250 | Phosphatidylinositol-speciwc phospholipase C4 |  |  |  | 1.052401334 | 0.009097 |
| **Cs3g14760** | **Phospholipase D P2** | **4.46269977** | **5.61E-75** |  | **2.458377404** | **1.78E-44** |
| Cs1g18410 | Phospholipase D alpha 4 | 1.34652685 | 0.004506 |  |  |  |
| Cs5g31250 | PATATIN-like protein 9, IIIB isoform 1 | -2.490041109 | 4.58E-30 |  |  |  |
| Cs2g10480 | Patatin group A-3-like | -1.54524875 | 1.86E-14 |  |  |  |
| orange1.1t05309 | Patatin group A-3-like |  |  |  | -1.87339106 | 7.02E-05 |
| **Cs8g17090** | **Patatin-like protein** | **-1.185596955** | **5.84E-14** |  | **1.167662832** | **1.06E-26** |
| Cs8g17080 | Patatin-like protein |  |  |  | 1.174514228 | 1.67E-05 |
| Cs5g08030 | Alpha/beta-Hydrolases superfamily protein | **-2.712036615** | **1.30E-42** |  |  |  |
| Cs7g06350 | Calmodulin-binding heat shock protein | 2.934777727 | 0.010453 |  |  |  |
| Cs3g23440 | Feruloyl esterase A |  |  |  | 2.057019781 | 1.36E-55 |
|  |  |  |  |  |  |  |
| Phospholipid biosynthesis | |  |  |  |  |  |
| **Cs6g20410** | **Glycerol-3-phosphate acyltransferase 6** | **-3.919230434** | **2.04E-39** |  | **-2.260841714** | **5.08E-24** |
| **Cs7g04640** | **Glycerol-3-phosphate acyltransferase 6** | **-3.461091041** | **6.20E-11** |  | **-1.841998224** | **0.000547** |
| orange1.1t02147 | Beta-hexosaminidase-like | -1.114244983 | 5.51E-05 |  |  |  |
| **Cs7g01060** | **Beta-hexosaminidase-like** | **-2.640800576** | **1.14E-26** |  | **-1.503885828** | **2.04E-24** |
| Cs2g12170 | Beta-hexosaminidase 3 isoform 1 |  |  |  | 1.052921641 | 3.17E-32 |
| orange1.1t03555 | Phosphatidylserine decarboxylase 2 | 1.810106769 | 1.26E-07 |  |  |  |
| orange1.1t04183 | Choline/ethanolamine kinase | 2.237532129 | 2.51E-19 |  |  |  |
| Cs2g11540 | Choline-phosphate cytidylyltransferase B-like isoform 1 | 1.155311354 | 0.002851 |  |  |  |
| Cs1g12410 | Phosphatidic acid phosphatase-related protein | -1.615872706 | 6.87E-17 |  |  |  |
|  |  |  |  |  |  |  |
| Glycolipid biosynthesis | |  |  |  |  |  |
| **Cs4g05250** | **Monogalactosyldiacylglycerol synthase 2** | **3.93812013** | **1.47E-67** |  | **1.332720729** | **1.37E-24** |
| Cs7g06070 | Monogalactosyldiacylglycerol synthase 2 | 6.654168074 | 6.00E-16 |  |  |  |
| Cs6g13680 | UDP-glycosyltransferase superfamily protein isoform 1 | 1.124759955 | 9.78E-10 |  |  |  |
|  |  |  |  |  |  |  |
| Steroid biosynthesis | |  |  |  |  |  |
| Cs8g03350 | Squalene monooxygenase 3 | -3.693093677 | 0.010552 |  |  |  |
| Cs8g03530 | Squalene monooxygenase 3 | -3.034759399 | 0.001150 |  |  |  |
| orange1.1t05606 | Squalene monooxygenase-like | -4.06443907 | 0.003783 |  |  |  |
| **Cs4g04640** | **Squalene epoxidase 1 protein** | **-4.276646107** | **2.31E-22** |  | **-2.387574731** | **3.47E-07** |
| Cs4g04650 | Squalene epoxidase 1 protein | -3.263071064 | 0.000622 |  |  |  |
| Cs4g04670 | Squalene epoxidase 1 protein | -5.439442003 | 2.35E-09 |  |  |  |
| Cs4g04710 | Squalene epoxidase 1 protein | -4.234210521 | 0.000680 |  |  |  |
| Cs2g03520 | Cytochrome P450, family 710, subfamily A, polypeptide 1 | -2.499335606 | 2.88E-30 |  |  |  |
| **Cs4g04630** | **Cycloartenol synthase-like** | **-4.636955085** | **3.07E-25** |  | **-1.400688544** | **3.95E-05** |
|  |  |  |  |  |  |  |
| Lipip transport | |  |  |  |  |  |
| Cs3g21010 | Pleckstrin homology domain-containing family A member 8-like | 1.432156491 | 1.78E-12 |  |  |  |
| XLOC_011317 | Bifunctional inhibitor/lipid-transfer protein/seed storage 2S albumin superfamily protein | 1.354422526 | 0.016606 |  |  |  |
| Cs5g31000 | Bifunctional inhibitor/lipid-transfer protein/seed storage 2S albumin superfamily protein |  |  |  | 1.067603337 | 3.85E-05 |
| Cs6g09960 | Lipid transfer protein precursor |  |  |  | 2.48784482 | 0.023042 |
| Cs6g09970 | Lipid transfer protein precursor |  |  |  | 1.919787483 | 0.013334 |
|  |  |  |  |  |  |  |
| Other genes related to lipid metabolism | |  |  |  |  |  |
| Cs1g24410 | Dolichyldiphosphatase 1 | 1.142533681 | 1.57E-05 |  |  |  |
| orange1.1t01392 | Glycerol kinase-like |  |  |  | 9.218405821 | 0.043442 |
| Cs5g12710 | UDP-N-acetylglucosamine-N-acetylmuramyl-(pentapeptide) pyrophosphoryl-undecaprenol N-acetylglucosamine transferase-like |  |  |  | 1.035123534 | 1.68E-15 |
|  |  |  |  |  |  |  |
| Fatty acid metabolism | |  |  |  |  |  |
| orange1.1t02015 | NAD(P)-binding Rossmann-fold superfamily protein isoform 1 | -1.249893003 | 1.51E-09 |  |  |  |
| Cs2g06240 | Sphingolipid delta(4)-desaturase DES1-like | -1.294546434 | 0.000719 |  |  |  |
| Cs3g15530 | Stearoyl-acyl-carrier protein desaturase | -2.119181812 | 0.000177 |  |  |  |
| **Cs3g20840** | **Stearoyl-ACP desaturase** | **-1.426399196** | **0.000385** |  | **2.260236071** | **1.53E-169** |
| Cs4g17260 | 3-ketoacyl-CoA synthase | -2.322392005 | 1.70E-33 |  |  |  |
| orange1.1t00556 | 3-ketoacyl-CoA synthase 1 |  |  |  | 1.197740825 | 4.19E-49 |
| Cs5g01990 | 3-ketoacyl-acyl carrier protein synthase I | -3.324988497 | 6.83E-50 |  |  |  |
| Cs6g08600 | Omega-3 fatty acid desaturase | -2.063012838 | 2.04E-20 |  |  |  |
| Cs8g20310 | Omega-3 fatty acid desaturase, chloroplastic-like |  |  |  | 1.062385058 | 6.08E-54 |
| orange1.1t02024 | Omega-6 fatty acid desaturase | -1.213510736 | 0.000483 |  |  |  |
| Cs4g04540 | Biotin carboxylase 1, chloroplastic isoform 1 | -1.098826825 | 7.98E-07 |  |  |  |
| Cs6g22090 | Biotin carboxyl carrier protein subunit of of Het-ACCase (BCCP2) | -1.10517754 | 2.20E-08 |  |  |  |
| **Cs4g06470** | **Acyl carrier protein 1** | **-4.325539162** | **2.07E-41** |  | **-2.656416486** | **1.37E-28** |
| Cs4g06430 | Fiddlehead-like protein |  |  |  | 3.008511743 | 0.001163 |
| Cs6g08630 | Acyl-CoA oxidase 4 | 1.140358862 | 2.71E-11 |  |  |  |
| Cs2g13450 | Myristoyl-acyl carrier protein thioesterase, chloroplastic | -2.937170727 | 7.79E-09 |  |  |  |
| Cs7g05030 | Acyl-protein thioesterase 2 | 1.129867448 | 0.000592 |  |  |  |
| orange1.1t05197 | Acyl-protein thioesterase 2 isoform 1 | 1.404942404 | 1.33E-06 |  |  |  |
| Cs3g13870 | Long-chain acyl-CoA synthetase 7 isoform 1 | 1.375224521 | 0.000276 |  |  |  |
| Cs3g19600 | Long chain acyl-CoA synthetase 9 isoform 1 | -1.428623115 | 4.16E-07 |  |  |  |
| Cs9g02550 | Medium-chain-fatty-acid-CoA ligase | -1.446911116 | 2.06E-05 |  |  |  |
| orange1.1t01519 | Cytochrome P450 77A3-like |  |  |  | -1.124411379 | 4.92E-20 |
| Cs9g02540 | Acetate/butyrate-CoA ligase AAE7, peroxisomal-like |  |  |  | -1.489013599 | 7.07E-07 |
| Cs3g20800 | Alcohol dehydrogenase |  |  |  | 2.313098306 | 5.29E-218 |
| Cs3g20810 | Alcohol dehydrogenase |  |  |  | 1.832569477 | 2.25E-41 |
| XLOC_018029 | Trans-2,3-enoyl-CoA reductase-like |  |  |  | -1.670712843 | 4.83E-19 |

*DEGs shared by the two citrus species were highlighted in bold. FC, fold change; FDR, false discovery rate.*

**TABLE S13 | DEGs related to signal transduction, hormone biosynthesis and degradation in Al-treated *C. sinensis* and *C. grandis* roots.**

| **ID** | **Description** | ***C. grandis*** | |  | ***C. sinensis*** | |
| --- | --- | --- | --- | --- | --- | --- |
|  |  | **log2 (FC)** | **FDR** |  | **log2 (FC)** | **FDR** |
| Protein phosphorylation | |  |  |  |  |  |
| **Cs1g04380** | **Serine/threonine-protein kinase** | **-3.208609544** | **1.66E-21** |  | **-1.106828005** | **5.99E-06** |
| **Cs3g21740** | **Serine/threonine-protein kinase** | **-1.475242007** | **5.15E-05** |  | **-1.623183805** | **0.006957** |
| **Cs4g05990** | **Serine/threonine-protein kinase** | **-3.671077008** | **1.15E-29** |  | **-2.88336706** | **8.35E-16** |
| Cs4g18970 | Serine/threonine-protein kinase Aurora-1 |  |  |  | 1.046806208 | 4.47E-16 |
| Cs3g16980 | Probable serine/threonine-protein kinase tsuA-like |  |  |  | 1.018337012 | 2.96E-17 |
| Cs2g12940 | Probable serine/threonine-protein kinase At1g18390 | -1.057352165 | 0.000204 |  |  |  |
| Cs2g13150 | Probable serine/threonine-protein kinase nek3-like | 1.061280679 | 0.029251 |  |  |  |
| Cs6g11640 | Serine/threonine-protein kinase-like protein CCR1-like |  |  |  | 1.355937819 | 3.93E-09 |
| Cs3g17530 | Serine/threonine-protein kinase-like protein At1g28390-like | -1.12946994 | 2.63E-07 |  |  |  |
| Cs3g11360 | Serine/threonine-protein kinase WNK1-like | -1.096137337 | 1.45E-05 |  |  |  |
| Cs3g14060 | Inactive serine/threonine-protein kinase At1g67470-like | 1.452815746 | 0.002357 |  |  |  |
| **Cs4g02880** | **LRR receptor-like serine/threonine-protein kinase** | **-1.366146397** | **0.000141** |  | **-1.47438296** | **0.002992** |
| Cs5g15210 | Leucine-rich repeat receptor-like serine/threonine-protein kinase At1g17230-like |  |  |  | -1.268369845 | 2.02E-06 |
| Cs3g18340 | Probable LRR receptor-like serine/threonine-protein kinase At4g08850-like |  |  |  | 3.450603253 | 0.033859 |
| Cs7g21340 | Probable LRR receptor-like serine/threonine-protein kinase At4g08850-like |  |  |  | 1.942042278 | 9.92E-09 |
| orange1.1t04224 | Probable LRR receptor-like serine/threonine-protein kinase At4g08850-like |  |  |  | 1.075688075 | 0.029365 |
| Cs4g12920 | Probable LRR receptor-like serine/threonine-protein kinase At4g08850-like | 2.614305619 | 0.047602 |  |  |  |
| Cs6g20930 | Probable LRR receptor-like serine/threonine-protein kinase At4g08850-like | 3.78149784 | 0.004770 |  |  |  |
| Cs5g29220 | Probable LRR receptor-like serine/threonine-protein kinase At4g08850-like | -2.433678113 | 4.18E-09 |  |  |  |
| Cs5g29260 | Probable LRR receptor-like serine/threonine-protein kinase At4g08850-like | -1.375932099 | 0.015138 |  |  |  |
| Cs5g24140 | Probable LRR receptor-like serine/threonine-protein kinase At4g08850-like | 1.598234482 | 0.008637 |  |  |  |
| orange1.1t03965 | Probable LRR receptor-like serine/threonine-protein kinase At4g08850-like | -3.26022709 | 0.011271 |  |  |  |
| TCONS_00016528 | Probable LRR receptor-like serine/threonine-protein kinase At4g08850-like | 2.678013227 | 1.70E-07 |  |  |  |
| Cs5g28620 | Probable LRR receptor-like serine/threonine-protein kinase At3g47570-like, partial |  |  |  | -1.450228948 | 0.001033 |
| Cs7g20870 | Probable LRR receptor-like serine/threonine-protein kinase At3g47570-like |  |  |  | 1.201464612 | 1.92E-33 |
| **orange1.1t04505** | **Probable LRR receptor-like serine/threonine-protein kinase At3g47570-like** | **-9.314902675** | **0.004069** |  | **1.430630159** | **0.025844** |
| Cs5g28640 | Probable LRR receptor-like serine/threonine-protein kinase At3g47570-like, partial | 1.209957947 | 0.000383 |  |  |  |
| Cs6g01370 | Probable LRR receptor-like serine/threonine-protein kinase At3g47570-like | -2.450578013 | 0.000686 |  |  |  |
| Cs7g20890 | Probable LRR receptor-like serine/threonine-protein kinase At3g47570-like | -2.133323616 | 3.94E-05 |  |  |  |
| orange1.1t01548 | Probable LRR receptor-like serine/threonine-protein kinase At3g47570-like | -2.346669218 | 1.30E-05 |  |  |  |
| Cs1g16130 | Probable LRR receptor-like serine/threonine-protein kinase At1g51880-like |  |  |  | -2.913424672 | 2.86E-11 |
| Cs1g11660 | Receptor-like serine/threonine-protein kinase SD1-8-like |  |  |  | -1.051055657 | 0.041580 |
| Cs1g16060 | Probable LRR receptor-like serine/threonine-protein kinase At1g51880-like | 1.016956193 | 0.002103 |  |  |  |
| Cs1g16090 | Probable LRR receptor-like serine/threonine-protein kinase At1g51880-like | 1.087012297 | 2.85E-06 |  |  |  |
| Cs7g07680 | Probable LRR receptor-like serine/threonine-protein kinase At4g31250-like | -2.64152156 | 1.26E-22 |  |  |  |
| Cs5g26630 | LRR receptor-like serine/threonine-protein kinase GSO1 | 1.035034962 | 3.02E-05 |  |  |  |
| Cs1g05960 | G-type lectin S-receptor-like serine/threonine-protein kinase At4g27290-like |  |  |  | 1.296331683 | 0.000142 |
| Cs4g11670 | G-type lectin S-receptor-like serine/threonine-protein kinase At4g27290-like |  |  |  | -1.728909425 | 0.000207 |
| Cs7g07320 | G-type lectin S-receptor-like serine/threonine-protein kinase At1g34300-like |  |  |  | -2.978019122 | 3.67E-05 |
| Cs7g07340 | G-type lectin S-receptor-like serine/threonine-protein kinase At1g34300-like |  |  |  | -1.635986022 | 0.016823 |
| TCONS_00008704 | G-type lectin S-receptor-like serine/threonine-protein kinase At5g24080-like |  |  |  | 1.139692427 | 4.24E-07 |
| **Cs5g08290** | **G-type lectin S-receptor-like serine/threonine-protein kinase RLK1-like** | **-2.109530095** | **3.47E-05** |  | **2.008119797** | **2.19E-20** |
| **Cs5g08310** | **G-type lectin S-receptor-like serine/threonine-protein kinase RLK1-like** | **-2.799758116** | **1.70E-07** |  | **1.365843482** | **0.003679** |
| Cs1g05200 | G-type lectin S-receptor-like serine/threonine-protein kinase At1g11330-like | 3.768087316 | 4.01E-07 |  |  |  |
| Cs1g11970 | G-type lectin S-receptor-like serine/threonine-protein kinase At1g11300-like | 3.712437257 | 0.033750 |  |  |  |
| Cs1g14920 | G-type lectin S-receptor-like serine/threonine-protein kinase CES101-like | 1.541784444 | 3.76E-10 |  |  |  |
| Cs4g11630 | G-type lectin S-receptor-like serine/threonine-protein kinase B120-like | 1.104965745 | 4.40E-08 |  |  |  |
| Cs4g11660 | G-type lectin S-receptor-like serine/threonine-protein kinase At4g27290-like | 1.953519857 | 0.002025 |  |  |  |
| Cs9g06740 | G-type lectin S-receptor-like serine/threonine-protein kinase RLK1-like | 8.745305243 | 0.014031 |  |  |  |
| Cs7g01300 | G-type lectin S-receptor-like serine/threonine-protein kinase RLK1-like | -1.235996161 | 0.000378 |  |  |  |
| **Cs7g05270** | **Probable L-type lectin-domain containing receptor kinase S.7-like** | **-3.937519097** | **5.07E-20** |  | **-2.372435725** | **4.12E-105** |
| **Cs7g05280** | **Probable L-type lectin-domain containing receptor kinase S.7-like** | **-3.356114804** | **1.26E-22** |  | **-2.09918961** | **1.05E-24** |
| Cs7g05260 | Probable L-type lectin-domain containing receptor kinase S.7-like | -3.222280484 | 0.028818 |  |  |  |
| Cs7g05290 | Probable L-type lectin-domain containing receptor kinase S.7-like | -4.010169661 | 7.87E-12 |  |  |  |
| Cs8g12370 | L-type lectin-domain containing receptor kinase IX.1-like | -1.827461129 | 1.16E-15 |  |  |  |
| **Cs4g11610** | **S-locus lectin protein kinase family protein** | **4.700593188** | **6.19E-33** |  | **3.033624817** | **0.011707** |
| **Cs4g11760** | **S-locus lectin protein kinase family protein** | **1.03729936** | **0.004997** |  | **-1.230629079** | **0.007637** |
| Cs4g11750 | S-locus lectin protein kinase family protein | 1.504865831 | 3.98E-05 |  |  |  |
| Cs4g11800 | S-locus lectin protein kinase family protein | 1.045918293 | 0.038688 |  |  |  |
| Cs4g11830 | S-locus lectin protein kinase family protein | 1.04778195 | 0.000442 |  |  |  |
| Cs3g19300 | S-locus lectin protein kinase family protein | 1.49085138 | 0.028313 |  |  |  |
| Cs3g19310 | S-locus lectin protein kinase family protein | 1.267268093 | 6.71E-10 |  |  |  |
| Cs2g31130 | Probable leucine-rich repeat receptor-like protein kinase At1g35710-like |  |  |  | 1.407027934 | 2.05E-08 |
| Cs2g31150 | Probable leucine-rich repeat receptor-like protein kinase At1g35710-like |  |  |  | 1.888917512 | 3.81E-54 |
| Cs4g02070 | Leucine-rich receptor-like protein kinase family protein |  |  |  | -1.767721144 | 1.69E-12 |
| **Cs5g17200** | **Probable leucine-rich repeat receptor-like protein kinase At1g68400-like** | **-3.119798561** | **3.13E-07** |  | **-1.587873746** | **0.035908** |
| **Cs4g04330** | **Leucine-rich repeat transmembrane protein kinase** | **-3.190009045** | **2.78E-08** |  | **-1.676515955** | **0.008860** |
| Cs5g13540 | Leucine-rich repeat transmembrane protein kinase isoform 1 |  |  |  | 1.919237967 | 1.91E-77 |
| Cs6g22320 | Leucine-rich repeat transmembrane protein kinase |  |  |  | -1.442866803 | 0.000509 |
| Cs8g12550 | Leucine-rich repeat protein kinase family protein | 1.434086568 | 0.013037 |  |  |  |
| Cs8g12570 | Leucine-rich repeat protein kinase family protein | 1.161762633 | 0.000733 |  |  |  |
| Cs2g31120 | Probable leucine-rich repeat receptor-like protein kinase At1g35710-like | -1.749039618 | 9.56E-09 |  |  |  |
| Cs2g31150 | Probable leucine-rich repeat receptor-like protein kinase At1g35710-like | -1.604597044 | 5.29E-06 |  |  |  |
| Cs3g23200 | Leucine-rich repeat protein kinase family protein isoform 1 | -1.461840116 | 2.69E-10 |  |  |  |
| Cs4g04320 | Leucine-rich repeat-containing protein 40-like | -1.614492474 | 0.000986 |  |  |  |
| Cs5g02650 | Leucine-rich repeat protein kinase family protein isoform 1 | -1.044688645 | 0.004136 |  |  |  |
| Cs5g06510 | Leucine-rich repeat protein kinase family protein | -1.791688403 | 1.17E-13 |  |  |  |
| Cs5g07100 | Leucine-rich repeat protein kinase family protein isoform 1 | -1.002740436 | 0.000843 |  |  |  |
| Cs5g12990 | Leucine-rich repeat transmembrane protein kinase | 2.163082627 | 0.031053 |  |  |  |
| Cs9g18620 | Leucine-rich repeat protein kinase family protein | -1.125487667 | 2.52E-07 |  |  |  |
| orange1.1t01599 | Leucine-rich repeat transmembrane protein kinase isoform 1 | -1.086016476 | 0.008790 |  |  |  |
| Cs4g19300 | Probably inactive leucine-rich repeat receptor-like protein kinase | 1.187406508 | 8.30E-05 |  |  |  |
| Cs4g18430 | Probable leucine-rich repeat receptor-like protein kinase At1g68400-like | -2.211665046 | 7.24E-12 |  |  |  |
| Cs3g16610 | Leucine-rich receptor-like protein kinase family protein, XI-23,RLK7 | -1.245611313 | 5.64E-09 |  |  |  |
| Cs3g26340 | Leucine-rich receptor-like protein kinase family protein | 1.011186226 | 0.000278 |  |  |  |
| Cs7g14680 | Leucine-rich receptor-like protein kinase family protein | -1.039546548 | 0.021006 |  |  |  |
| Cs1g25070 | Leucine-rich receptor-like protein kinase family protein | -1.156229811 | 0.002049 |  |  |  |
| Cs2g13380 | Kinase-like protein |  |  |  | 1.328197352 | 0.017099 |
| **Cs5g08140** | **Kinase-like protein** | **-2.354057573** | **2.65E-09** |  | **1.361388698** | **6.09E-11** |
| Cs5g08330 | Kinase-like protein |  |  |  | 1.354465359 | 3.59E-06 |
| Cs5g08320 | Kinase-like protein | -2.390318156 | 1.38E-35 |  |  |  |
| Cs2g30830 | Kinase family protein |  |  |  | 1.145464136 | 0.027118 |
| Cs3g22470 | Kinase family protein with leucine-rich repeat domain | -1.407791159 | 2.36E-09 |  |  |  |
| Cs9g04830 | Kinase superfamily protein isoform 1 |  |  |  | 1.498369348 | 4.41E-10 |
| Cs1g17460 | Kinase superfamily protein |  |  |  | -1.517672249 | 0.028546 |
| Cs5g15220 | Kinase superfamily protein | -1.332436929 | 0.000234 |  |  |  |
| Cs4g07400 | Kinase superfamily protein | -1.395099501 | 1.45E-11 |  |  |  |
| Cs3g07530 | Kinase superfamily protein | -1.223484286 | 1.79E-07 |  |  |  |
| orange1.1t01537 | Kinase superfamily protein | -1.690307644 | 0.013824 |  |  |  |
| **Cs3g06380** | **Kinase protein with adenine nucleotide alpha hydrolases-like domain** | **-1.034977796** | **7.05E-05** |  | **-1.560540983** | **4.51E-28** |
| Cs7g07690 | Kinase C-like zinc finger protein | 1.693142896 | 9.29E-19 |  |  |  |
| Cs1g23560 | Probable receptor-like protein kinase At1g49730 |  |  |  | -1.461336201 | 5.31E-08 |
| Cs5g12930 | Probable receptor-like protein kinase At1g67000-like |  |  |  | 1.195018543 | 4.07E-12 |
| **Cs5g26070** | **Probable receptor-like protein kinase At1g11050-like** | **-3.635829492** | **2.46E-26** |  | **-2.416167713** | **2.06E-30** |
| **Cs5g28800** | **Probable inactive receptor kinase RLK902-like** | **-2.989124405** | **0.000568** |  | **-3.791221274** | **0.011009** |
| **XLOC_008035** | **Probable inactive receptor-like protein kinase At1g65250-like, partial** | **2.37570879** | **9.23E-22** |  | **1.256263401** | **0.000686** |
| Cs7g21630 | Receptor protein kinase-like protein |  |  |  | 1.846671793 | 0.002970 |
| Cs2g12900 | Probable receptor-like protein kinase At1g67000-like | -1.792905933 | 0.008291 |  |  |  |
| Cs2g13320 | Probable receptor-like protein kinase At1g67000-like | 2.002347115 | 1.00E-05 |  |  |  |
| Cs4g15510 | Receptor-like protein kinase HSL1-like | -1.320373324 | 3.45E-08 |  |  |  |
| Cs4g17400 | Receptor protein kinase | -1.1538696 | 2.65E-06 |  |  |  |
| Cs5g02390 | Probable inactive receptor kinase At5g58300-like | -3.451567121 | 3.04E-11 |  |  |  |
| Cs5g07570 | Receptor protein kinase isoform 1 | -1.118155668 | 0.004651 |  |  |  |
| XLOC_030045 | Receptor protein kinase | -1.506524664 | 7.87E-07 |  |  |  |
| orange1.1t02709 | Receptor-like protein kinase FERONIA-like | -1.627094184 | 4.67E-11 |  |  |  |
| orange1.1t02715 | Receptor-like protein kinase FERONIA-like | -1.118562032 | 8.65E-08 |  |  |  |
| XLOC_006978 | Probable receptor-like protein kinase At1g11050 | -1.365867802 | 3.13E-08 |  |  |  |
| orange1.1t04474 | Somatic embryogenesis receptor kinase 2-like | -4.181277505 | 4.36E-19 |  |  |  |
| Cs6g10250 | Malectin/receptor-like protein kinase family protein | -1.199126141 | 5.32E-08 |  |  |  |
| Cs8g16140 | Receptor like protein kinase S.2-like | -1.561720785 | 7.09E-09 |  |  |  |
| **Cs2g12140** | **BAK1-interacting receptor-like kinase 1 isoform 1** | **-2.498009449** | **7.52E-19** |  | **-1.103372947** | **3.59E-14** |
| **Cs1g16040** | **Light repressible receptor protein kinase** | **1.56711967** | **0.000150** |  | **2.761567329** | **6.48E-12** |
| Cs6g09030 | Lectin receptor-like kinase |  |  |  | 4.45676827 | 0.000478 |
| Cs5g14090 | Cysteine-rich receptor-like protein kinase 10-like |  |  |  | -2.113340418 | 0.000127 |
| **Cs5g33490** | **Cysteine-rich receptor-like protein kinase 11-like** | **-4.75012684** | **6.84E-20** |  | **-2.62522801** | **1.83E-13** |
| Cs8g03430 | Cysteine-rich receptor-like protein kinase 29 | 1.779543789 | 0.000428 |  |  |  |
| Cs8g03480 | Cysteine-rich receptor-like protein kinase 29 | 2.300640536 | 4.47E-06 |  |  |  |
| Cs4g01430 | Cysteine-rich RLK 34 | 1.730722469 | 1.86E-05 |  |  |  |
| Cs3g20500 | Proline-rich receptor-like protein kinase | 1.01306507 | 2.41E-06 |  |  |  |
| Cs7g04160 | Protein kinase 2B, chloroplastic-like | -2.43694314 | 4.03E-05 |  |  |  |
| orange1.1t02215 | Probable protein kinase At2g41970-like |  |  |  | 2.470715636 | 0.023042 |
| Cs4g01210 | Dual specificity protein kinase pyk2 | -1.006008888 | 0.002104 |  |  |  |
| Cs2g01040 | NIMA-related kinase 7 | 1.522789551 | 4.84E-06 |  |  |  |
| Cs5g17510 | Cytokinin-regulated kinase 1 | -3.869699304 | 7.35E-48 |  |  |  |
| Cs5g32830 | Cyclin-dependent kinase 12-like | 1.721369237 | 1.99E-23 |  |  |  |
| Cs1g25650 | Histidine kinase 1 |  |  |  | -1.084778971 | 4.46E-31 |
| Cs9g16420 | PTI1-like tyrosine-protein kinase At3g15890-like | -1.019557145 | 0.034135 |  |  |  |
| orange1.1t01739 | Shaggy-related kinase 11, 11 isoform 1 | -1.011202592 | 1.45E-06 |  |  |  |
| orange1.1t03917 | No lysine kinase 4 isoform 1 | 1.87688319 | 8.45E-19 |  |  |  |
| **Cs3g04510** | **Protein NSP-INTERACTING KINASE 2-like** | **-3.30181304** | **1.71E-08** |  | **-2.826691145** | **3.52E-13** |
| **XLOC_011433** | **Concanavalin A-like lectin protein kinase family protein** | **-1.553659994** | **1.21E-08** |  | **-1.161236554** | **7.85E-13** |
| **Cs6g09730** | **CBL-interacting protein kinase 22** | **1.416596415** | **2.72E-17** |  | **1.091743441** | **1.94E-105** |
| **orange1.1t00555** | **CBL-interacting protein kinase 9** | **1.111556034** | **5.43E-05** |  | **1.892863899** | **3.00E-05** |
| orange1.1t01385 | Receptor kinase | 1.45359905 | 0.008447 |  |  |  |
| **orange1.1t00355** | **Seven transmembrane domain-containing tyrosine-protein kinase 1** | **-2.838561064** | **2.91E-11** |  | **-1.87392108** | **0.020481** |
| **Cs5g04260** | **Hypersensitive-induced response protein** | **-4.39316662** | **1.76E-73** |  | **-2.120770148** | **4.86E-51** |
| Cs1g19230 | Expressed protein | 1.869703324 | 2.87E-17 |  |  |  |
| Cs4g04570 | U-box domain-containing protein 35-like | 1.565452778 | 0.033499 |  |  |  |
| orange1.1t00371 | U-box domain-containing protein 33-like | 1.437566783 | 7.57E-14 |  |  |  |
| **Cs8g17360** | Mitogen-activated protein kinase 1 | -1.569855247 | 5.84E-14 |  |  |  |
| Cs2g02820 | Mitogen-activated protein kinase kinase kinase A-like | 1.605636558 | 7.94E-11 |  |  |  |
| Cs1g22540 | Mitogen-activated protein kinase kinase kinase 1-like | -2.713239406 | 3.94E-31 |  |  |  |
| Cs4g19370 | Mitogen-activated protein kinase kinase kinase | 1.552462112 | 0.020282 |  |  |  |
| orange1.1t03798 | Mitogen-activated protein kinase kinase kinase 1-like |  |  |  | -1.374987147 | 2.88E-05 |
| Cs3g05150 | Wall-associated receptor kinase-like 1-like |  |  |  | -2.225457126 | 1.24E-05 |
| Cs3g14050 | Wall-associated receptor kinase-like 8-like |  |  |  | -1.433418024 | 0.008532 |
| **Cs3g14840** | **Wall-associated receptor kinase-like 20** | **-3.470549963** | **3.14E-44** |  | **-1.010392618** | **4.02E-26** |
| Cs9g18010 | Wall-associated receptor kinase and RING-finger domain-containing protein |  |  |  | -1.045963179 | 0.029666 |
| Cs1g13880 | Wall-associated receptor kinase-like 10-like, partial | 7.725478738 | 0.027656 |  |  |  |
| Cs1g13900 | Wall-associated receptor kinase-like 22-like | 1.182944151 | 0.000215 |  |  |  |
| Cs1g13910 | Wall-associated receptor kinase-like 22-like | 4.270436291 | 1.12E-06 |  |  |  |
| Cs3g13910 | Wall-associated receptor kinase-like 8-like | 1.394651127 | 0.001737 |  |  |  |
| Cs5g01020 | Wall-associated receptor kinase-like 10-like | -2.688064127 | 1.62E-12 |  |  |  |
| Cs8g13940 | Wall-associated receptor kinase 3 | 3.469847965 | 0.017749 |  |  |  |
| XLOC_000633 | Wall-associated receptor kinase-like 9-like | 3.057118942 | 0.015645 |  |  |  |
| orange1.1t04255 | Wall-associated receptor kinase-like 10-like, partial | 2.074747785 | 0.001954 |  |  |  |
| orange1.1t04036 | Wall-associated receptor kinase-like 2-like | -1.802807368 | 1.43E-20 |  |  |  |
|  |  |  |  |  |  |  |
| Protein dephosphorylation | |  |  |  |  |  |
| Cs1g26640 | Probable protein phosphatase 2C 47-like |  |  |  | 1.068303905 | 0.000645 |
| Cs9g16360 | Probable protein phosphatase 2C 75-like |  |  |  | 1.192169398 | 3.38E-13 |
| Cs8g19140 | Highly ABA-induced PP2C gene 2 isoform 1 |  |  |  | -1.01097957 | 2.72E-21 |
| Cs9g03785 | Phosphatase 2C family protein isoform 1 |  |  |  | -1.279476473 | 5.72E-66 |
| Cs1g15750 | Phosphatase 2C family protein | 1.779968857 | 0.015503 |  |  |  |
| Cs5g19840 | Probable protein phosphatase 2C 2-like | 1.111034827 | 2.86E-06 |  |  |  |
| Cs7g21650 | dual protein phosphatase 4 | 1.116357732 | 1.24E-08 |  |  |  |
| Cs8g18430 | Probable protein phosphatase 2C 23-like isoform 2 | -1.73147919 | 3.08E-14 |  |  |  |
| Cs4g18770 | Probable protein phosphatase 2C 72-like | -1.470335672 | 1.68E-12 |  |  |  |
| Cs8g02370 | Serine/threonine protein phosphatase 2A 57 kDa regulatory subunit B' beta isoform 1 | -2.735561048 | 5.64E-08 |  |  |  |
| Cs8g20420 | Phosphatase 2C family protein isoform 3 | -1.80188962 | 1.46E-16 |  |  |  |
| Cs6g10580 | Type one serine/threonine protein phosphatase 4 | -2.736203672 | 0.000313 |  |  |  |
|  |  |  |  |  |  |  |
| Ca/calmodulin-mediated signal transduction | |  |  |  |  |  |
| Cs3g14940 | Calmodulin-binding protein isoform 1 |  |  |  | 1.104913579 | 4.13E-21 |
| Cs4g18280 | Calmodulin-binding family protein isoform 1 | -1.611569271 | 3.16E-14 |  |  |  |
| Cs3g19070 | EF hand calcium-binding family protein |  |  |  | -1.512077572 | 2.31E-36 |
| Cs3g24200 | Transducin/WD40 repeat-like superfamily protein |  |  |  | 1.885406246 | 0.035553 |
| Cs4g16180 | Transducin/WD40 repeat-like superfamily protein | -1.048353573 | 0.002446 |  |  |  |
| Cs4g14630 | Calmodulin-binding family protein |  |  |  | 2.40790351 | 7.06E-17 |
| Cs7g06350 | Calmodulin-binding heat shock protein | 2.934777727 | 0.010453 |  |  |  |
| Cs5g28300 | Calcium-binding protein CML24 |  |  |  | 1.886737187 | 0.008070 |
| **orange1.1t03719** | **Calmodulin 8 isoform 1** | **-1.666658517** | **0.005257** |  | **1.149372425** | **4.33E-07** |
| **Cs7g02680** | **Annexin-like protein RJ4 isoform 2** | **-2.010593928** | **4.85E-25** |  | **-1.154876593** | **3.76E-46** |
| **orange1.1t01651** | **Annexin D5** | **3.937924922** | **3.84E-39** |  | **1.461533541** | **0.045099** |
| Cs5g26910 | Annexin D8-like | -1.358742483 | 0.009208 |  |  |  |
| Cs7g02660 | Annexin D3-like | 1.340223878 | 2.88E-09 |  |  |  |
| Cs3g10710 | Calmodulin-like protein 11-like isoform 1 | 3.835247599 | 0.009298 |  |  |  |
| Cs4g01630 | Calmodulin-binding protein | -1.153258243 | 6.37E-07 |  |  |  |
| Cs5g07160 | Probable calcium-binding protein CML31-like | -3.293482346 | 1.13E-32 |  |  |  |
| orange1.1t04034 | Calcium-binding protein CML37 | -1.900995038 | 2.12E-23 |  |  |  |
| Cs9g07810 | Calmodulin-like protein 11-like isoform 2 | -1.212042205 | 5.94E-05 |  |  |  |
| orange1.1t00071 | Calcium-binding EF-hand family protein | -1.140534189 | 1.23E-07 |  |  |  |
| **Cs5g27350** | **C2 calcium/lipid-binding plant phosphoribosyltransferase family protein** | **-1.672886471** | **4.12E-09** |  | **1.034940668** | **4.48E-20** |
| orange1.1t00249 | Calmodulin-like protein 7-like | -1.535877497 | 0.035422 |  |  |  |
|  |  |  |  |  |  |  |
| Hormone-mediated signal transduction | |  |  |  |  |  |
| Cs1g13970 | Auxin-induced protein AUX22-like |  |  |  | 1.756228202 | 1.52E-07 |
| Cs3g10920 | AUX/IAA transcriptional regulator family protein isoform 1 |  |  |  | 1.17811186 | 2.37E-09 |
| Cs4g17050 | AUX/IAA transcriptional regulator family protein |  |  |  | 3.687760773 | 0.019771 |
| Cs2g02080 | Auxin-responsive family protein |  |  |  | 1.50511175 | 1.52E-05 |
| Cs9g08100 | Auxin-induced protein 22D |  |  |  | 1.355048464 | 9.84E-45 |
| Cs9g08110 | Auxin-responsive protein |  |  |  | 1.080842064 | 9.44E-32 |
| Cs5g30380 | Indole-3-acetic acid inducible 14 isoform 1 |  |  |  | 1.030471908 | 5.56E-18 |
| Cs1g13960 | AUX/IAA transcriptional regulator family protein | -1.13596025 | 0.000708 |  |  |  |
| Cs5g30390 | Auxin-induced protein 22B-like | -1.57238497 | 0.000173 |  |  |  |
| Cs3g19250 | Auxin influx carrier protein | -1.114201778 | 1.57E-5 |  |  |  |
| Cs4g16220 | Auxin-responsive protein IAA27 isoform 1 | 1.145964714 | 0.035715 |  |  |  |
| orange1.1t01850 | Type-a response regulator |  |  |  | 1.673240094 | 1.39E-14 |
| Cs6g20220 | Gibberellin-regulated protein 14-like |  |  |  | 1.073162212 | 7.17E-43 |
| Cs5g29870 | Ethylene response factor 10 |  |  |  | 1.107558857 | 5.56E-18 |
| Cs4g14980 | Abscisic acid insensitivity 1B | 1.158305966 | 6.58E-11 |  |  |  |
| Cs4g07130 | Plastid jasmonates ZIM-domain protein | -1.325628847 | 6.19E-10 |  |  |  |
| Cs7g02820 | Protein TIFY 9 | -1.368835034 | 1.54E-11 |  |  |  |
| Cs2g13120 | Lipid phosphate phosphatase 2 isoform 2 | 1.309401345 | 3.05E-15 |  |  |  |
| Cs4g18220 | Beta-1,4-N-acetylglucosaminyltransferase family protein | -1.493055345 | 7.35E-07 |  |  |  |
|  |  |  |  |  |  |  |
| Phosphatidylinositol signal system | |  |  |  |  |  |
| Cs5g25570 | Inositol-tetrakisphosphate 1-kinase 1-like |  |  |  | 1.381678335 | 0.000215 |
| Cs5g30950 | Type I inositol-1,4,5-trisphosphate 5-phosphatase CVP2-like |  |  |  | 1.378350918 | 8.10E-06 |
| Cs7g22650 | Type I inositol-1,4,5-trisphosphate 5-phosphatase 2-like |  |  |  | -1.454440073 | 1.56E-61 |
| Cs8g20250 | Phosphatidylinositol-speciwc phospholipase C4 |  |  |  | 1.052401334 | 0.009097 |
| Cs7g14760 | Phospholipase C 2 | 1.231436744 | 1.20E-09 |  |  |  |
| **Cs3g14760** | **Phospholipase D P2** | **4.46269977** | **5.61E-75** |  | **2.458377404** | **1.78E-44** |
| Cs1g18410 | Phospholipase D alpha 4 | 1.34652685 | 0.004506 |  |  |  |
|  |  |  |  |  |  |  |
| Other signal transduction pathways | |  |  |  |  |  |
| Cs3g03030 | Ras-related small GTP-binding family protein |  |  |  | -1.682548513 | 0.009998 |
| **Cs7g21880** | **Monocopper oxidase-like protein SKU5-like** | **-4.121989642** | **3.93E-30** |  | **-2.736300737** | **1.15E-17** |
| **Cs7g21890** | **Cupredoxin superfamily protein** | **-4.786504034** | **1.36E-47** |  | **-1.922894706** | **1.15E-15** |
| Cs3g26640 | DC1 domain-containing protein isoform 3 |  |  |  | -1.022646972 | 4.55E-25 |
| Cs1g06800 | Toll-Interleukin-Resistance domain family protein | -11.83794718 | 3.97E-05 |  |  |  |
| Cs2g16150 | Glutaredoxin | -1.639314567 | 5.54E-14 |  |  |  |
| Cs6g11040 | Protein NDR1-like | -1.178062488 | 8.01E-08 |  |  |  |
| orange1.1t04334 | Guanine nucleotide-binding protein alpha-2 subunit | 1.00111145 | 0.000594 |  |  |  |
| **Cs5g28250** | **Extra-large G-protein 1** | **2.397447884** | **5.87E-38** |  | **1.196252255** | **4.09E-36** |
| Cs5g28260 | Extra-large G-protein 1 | 1.309945318 | 8.37E-15 |  |  |  |
|  |  |  |  |  |  |  |
| Hormone biosynthesis and degradation | |  |  |  |  |  |
| Cs7g02410 | Cytokinin oxidase/dehydrogenase 6 isoform 1 |  |  |  | -1.208735664 | 0.009174 |
| Cs9g06010 | Cytokinin biosynthetic isopentenyltransferase |  |  |  | -1.253624078 | 1.92E-23 |
| orange1.1t01981 | UDP-glucosyltransferase family 1 protein | 2.025052453 | 0.010816 |  |  |  |
| orange1.1t01984 | UDP-glucosyltransferase family 1 protein | -1.728453564 | 2.41E-08 |  |  |  |
| Cs2g03380 | Gibberellin 2-beta-dioxygenase |  |  |  | 1.693238594 | 4.28E-71 |
| Cs4g20350 | Gibberellin 3-oxidase |  |  |  | -1.019232159 | 3.90E-09 |
| Cs5g14480 | Gibberellin 2-oxidase |  |  |  | 1.831926191 | 6.13E-08 |
| Cs9g16520 | Gibberellin 20-oxidase |  |  |  | -1.007014885 | 5.63E-09 |
| **orange1.1t00272** | **Gibberellin 20-oxidase 2** | **-1.863583005** | **0.008507** |  | **-2.138661745** | **0.000524** |
| Cs1g13750 | Gibberellin 20 oxidase 1-like | 1.523493823 | 4.71E-13 |  |  |  |
| Cs1g13820 | Gibberellin 20 oxidase 3 | 1.217925201 | 3.41E-11 |  |  |  |
| Cs1g13840 | gibberellin 3 oxidase 1 | -1.467124026 | 7.99E-07 |  |  |  |
| **Cs3g21210** | **Abscisic acid 8'-hydroxylase 3-like** | **-2.750644643** | **-2.750645** |  | **-1.911080681** | **2.21E-27** |
| Cs2g03270 | 9-cis-epoxycarotenoid dioxygenase 2 | -1.11455966 | 0.003760 |  |  |  |
| orange1.1t04051 | Zeaxanthin epoxidase | -4.678249955 | 2.55E-09 |  |  |  |
| Cs8g18780 | ABA 8'-hydroxylase |  |  |  | 2.239994415 | 1.72E-05 |
| orange1.1t00416 | ACC synthase |  |  |  | 1.151969425 | 0.000633 |
| Cs5g03060 | ACC synthase | -1.135367249 | 4.77E-07 |  |  |  |
| orange1.1t03606 | 1-aminocyclopropane-1-carboxylate oxidase homolog 1-like | 2.323192425 | 8.98E-25 |  |  |  |
| Cs7g02060 | Polyamine oxidase 1 isoform 1 | 1.782640432 | 4.59E-49 |  |  |  |
| Cs4g02260 | S-adenosylmethionine decarboxylase proenzyme-like | -1.271311845 | 3.86E-09 |  |  |  |
| orange1.1t03535 | Ornithine decarboxylase | -1.699352767 | 0.041105 |  |  |  |

*DEGs shared by the two citrus species were highlighted in bold. FC, fold change; FDR, false discovery rate.*

**TABLE S14 | Al-responsive TFs in *C. sinensis* and *C. grandis* roots.**

| **ID** | **Description** | ***C. grandis*** | |  | ***C. sinensis*** | |  |
| --- | --- | --- | --- | --- | --- | --- | --- |
|  |  | **log2 (FC)** | **FDR** |  | **log2 (FC)** | **FDR** |  |
| Cs5g34990 | Myb domain protein 79 isoform 1 |  |  |  | -1.013216023 | 2.12E-17 |  |
| Cs8g17920 | Myb domain protein 59 isoform 2 |  |  |  | -1.589138897 | 9.25E-104 |  |
| Cs2g07030 | Myb-related protein Myb4 |  |  |  | 2.382438784 | 5.78E-05 |  |
| Cs2g11880 | Transcription factor Myb |  |  |  | 1.183757521 | 1.25E-05 |  |
| Cs2g12700 | R2R3-MYB transcription factor |  |  |  | 1.746001837 | 0.000532 |  |
| Cs7g16380 | Myb domain protein 60 |  |  |  | 1.3488219 | 7.45E-08 |  |
| **Cs9g01750** | **R2R3 transcription factor MYB108-like protein 1** | **-1.751467655** | **1.25E-06** |  | **1.008842339** | **3.56E-13** |  |
| **Cs2g07040** | **Myb-related protein Myb4-like** | **-2.04672637** | **0.000389** |  | **1.771906363** | **9.46E-27** |  |
| **Cs5g25240** | **Myb domain protein 107** | **3.282091615** | **6.64E-06** |  | **1.324805026** | **0.001266** |  |
| **Cs6g08490** | **Myb-like HTH transcriptional regulator family protein isoform 1** | **-3.526614036** | **5.67E-17** |  | **-2.10709863** | **2.75E-20** |  |
| Cs1g06220 | Transcription factor MYB75-like | 1.127054761 | 0.015503 |  |  |  |  |
| Cs3g20590 | Myb domain protein 52 | 1.232873834 | 0.016142 |  |  |  |  |
| Cs8g02740 | MYB transcription factor | 1.105107114 | 1.52E-05 |  |  |  |  |
| Cs5g16420 | Myb-related protein B | 1.399885126 | 0.010133 |  |  |  |  |
| Cs8g15780 | MYB24 | 1.06335614 | 1.44E-10 |  |  |  |  |
| Cs4g18630 | Transcription factor MYB1R1 | 1.76470381 | 3.07E-08 |  |  |  |  |
| Cs2g10330 | Cyclin-D-binding Myb-like transcription factor 1 isoform 1 | 1.135177342 | 1.89E-10 |  |  |  |  |
| Cs2g15390 | Myb-like HTH transcriptional regulator family protein | 1.104066421 | 0.049103 |  |  |  |  |
| Cs3g23070 | R2R3-MYB transcription factor | -1.380864512 | 6.14E-10 |  |  |  |  |
| Cs5g27440 | Transcriptional activator Myb-like | -1.195057424 | 0.000191 |  |  |  |  |
| Cs3g23950 | Transcriptional activator Myb-like | -3.047378002 | 7.50E-41 |  |  |  |  |
| Cs6g21530 | Myb domain protein 106 | -9.091189406 | 0.000683 |  |  |  |  |
| Cs8g05000 | MYB domain class transcription factor | -1.487169091 | 2.51E-12 |  |  |  |  |
| Cs5g29830 | Myb-related transcription factor LBM1 | -1.535075164 | 5.61E-11 |  |  |  |  |
| orange1.1t00339 | Myb domain protein 17 isoform 1 | -1.764978039 | 0.047980 |  |  |  |  |
| orange1.1t01728 | Myb-like transcription factor Myb 5 | -2.600974762 | 2.14E-19 |  |  |  |  |
| Cs3g23270 | AP2/ERF domain-containing transcription factor |  |  |  | -1.157057089 | 3.66E-29 |  |
| Cs5g01140 | AP2/ERF domain-containing transcription factor |  |  |  | -1.074675416 | 5.56E-14 |  |
| orange1.1t02436 | AP2/ERF domain-containing transcription factor |  |  |  | -1.007406234 | 4.51E-11 |  |
| Cs8g10700 | AP2/ERF transcription factor |  |  |  | -1.012643798 | 4.98E-29 |  |
| Cs1g04650 | AP2/ERF domain-containing transcription factor |  |  |  | 3.442155992 | 1.06E-178 |  |
| Cs5g19600 | AP2/ERF domain-containing transcription factor |  |  |  | 1.373519881 | 0.010645 |  |
| **Cs5g08360** | **AP2/ERF domain-containing transcription factor** | **-2.390318156** | **1.38E-35** |  | **1.011875845** | **6.29E-44** |  |
| **Cs4g02210** | **AP2 domain-containing transcription factor** | **-1.988786279** | **1.44E-08** |  | **-2.310381707** | **2.37E-08** |  |
| **Cs6g20790** | **AP2 domain-containing transcription factor** | **-3.623092405** | **6.02E-21** |  | **-2.47134247** | **1.74E-18** |  |
| Cs3g19420 | AP2/ERF domain-containing transcription factor | -2.028488219 | 2.00E-12 |  |  |  |  |
| Cs4g07040 | AP2/ERF domain-containing transcription factor | -1.604544298 | 1.15E-14 |  |  |  |  |
| Cs4g01480 | AP2/ERF and B3 domain-containing transcription factor At1g51120-like | 1.648021277 | 9.72E-07 |  |  |  |  |
| orange1.1t00506 | AP2/ERF domain-containing transcription factor | -1.032856407 | 0.003409 |  |  |  |  |
| orange1.1t04058 | AP2 domain class transcription factor | -1.410358162 | 2.40E-11 |  |  |  |  |
| orange1.1t04769 | AP2/B3-like transcriptional factor family protein isoform 2 | 1.058346815 | 0.014677 |  |  |  |  |
| orange1.1t05528 | Transcription factor APETALA2 |  |  |  | -1.092375189 | 0.005857 |  |
| XLOC_013075 | Ethylene-responsive transcription factor ERF034-like |  |  |  | 1.462910996 | 1.11E-11 |  |
| Cs5g33540 | Ethylene responsive transcription factor 2b | -1.946837814 | 7.39E-26 |  |  |  |  |
| Cs9g16820 | Ethylene-responsive transcription factor ERF025-like | -3.009638949 | 1.04E-07 |  |  |  |  |
| XLOC_012134 | Ethylene-responsive transcription factor 4-like | -1.94941437 | 1.22E-05 |  |  |  |  |
| Cs2g05640 | ERF domain protein 12 | -1.570599904 | 3.86E-13 |  |  |  |  |
| **XLOC_021890** | **Protein SENSITIVE TO PROTON RHIZOTOXICITY 1-like** | **3.457510575** | **2.27E-51** |  | **2.308599125** | **1.68E-14** |  |
| **XLOC_021891** | **Protein SENSITIVE TO PROTON RHIZOTOXICITY 1-like** | **5.574499493** | **2.32E-87** |  | **4.620923285** | **2.87E-36** |  |
| Cs3g15900 | Cys2/His2-type zinc finger protein | -1.48291893 | 4.18E-13 |  |  |  |  |
| Cs3g19200 | C2H2-like zinc finger protein |  |  |  | 1.003976441 | 0.000141 |  |
| XLOC_013561 | Zinc finger C-x8-C-x5-C-x3-H type family protein isoform 2 |  |  |  | 3.256290953 | 0.002820 |  |
| Cs3g03600 | Zinc finger C-x8-C-x5-C-x3-H type family protein isoform 3 | 1.082447587 | 0.001308 |  |  |  |  |
| Cs4g18530 | Zinc finger C-x8-C-x5-C-x3-H type family protein isoform 1 | 2.429929447 | 0.012827 |  |  |  |  |
| orange1.1t03801 | Zinc finger C-x8-C-x5-C-x3-H type family protein isoform 3 | 1.04885771 | 2.03E-07 |  |  |  |  |
| Cs1g17090 | Zinc finger CCCH domain-containing protein 62 |  |  |  | 1.104188504 | 0.000918 |  |
| Cs8g08350 | Zinc finger CCCH domain-containing protein 11-like |  |  |  | 1.001213933 | 0.049732 |  |
| Cs6g21400 | Dof zinc finger protein DOF5.2 |  |  |  | -1.224025105 | 2.57E-38 |  |
| orange1.1t00521 | Dof zinc finger protein DOF3.7-like isoform 1 | 1.117106263 | 1.05E-07 |  |  |  |  |
| orange1.1t00904 | GATA zinc finger domain-containing protein 10 |  |  |  | 1.021317019 | 0.000101 |  |
| Cs9g04030 | Ring zinc finger transcription factor | -2.232761238 | 2.20E-33 |  |  |  |  |
| orange1.1t01779 | WRKY transcription factor 47-2 |  |  |  | -1.20305137 | 1.51E-16 |  |
| orange1.1t02600 | WRKY-type DNA binding protein 1 |  |  |  | -1.095690428 | 1.39E-36 |  |
| Cs2g02790 | WRKY family transcription factor |  |  |  | 1.387451925 | 6.03E-100 |  |
| Cs7g06330 | WRKY transcription factor 29 |  |  |  | 2.7281197 | 1.52E-06 |  |
| Cs9g03310 | WRKY transcription factor 58 |  |  |  | 1.164777815 | 4.34E-08 |  |
| **Cs6g10120** | **Probable WRKY transcription factor 70-like** | **2.351662059** | **1.27E-32** |  | **1.123127715** | **7.13E-30** |  |
| Cs1g04180 | WRKY DNA-binding protein 13 | 1.98323051 | 0.013646 |  |  |  |  |
| Cs3g23190 | WRKY DNA-binding protein 7 | -1.277405584 | 3.57E-08 |  |  |  |  |
| Cs6g09420 | WRKY transcription factor 17 | -2.215976473 | 3.24E-23 |  |  |  |  |
| Cs7g06320 | WRKY transcription factor 2 | -1.604640775 | 1.04E-10 |  |  |  |  |
| orange1.1t01713 | WRKY1 | -1.358837772 | 4.47E-09 |  |  |  |  |
| Cs5g16510 | Homeobox-leucine zipper protein HDG11-like |  |  |  | 1.128699826 | 0.000162 |  |
| Cs9g16980 | Homeobox-leucine zipper family protein / lipid-binding START domain-containing protein isoform 1 |  |  |  | 1.077021622 | 0.000998 |  |
| Cs1g15200 | HD-ZIP IV family of homeobox-leucine zipper protein with lipid-binding START domain isoform 1 |  |  |  | -1.034126361 | 1.30E-14 |  |
| Cs7g01220 | Homeobox-leucine zipper protein HAT14-like |  |  |  | -1.331795555 | 6.73E-18 |  |
| Cs6g13660 | BEL1-like homeodomain protein 1 isoform 1 |  |  |  | -1.147009277 | 1.50E-77 |  |
| **Cs4g17160** | **Homeodomain-like superfamily protein** | **1.045007** | **0.032046** |  | **-1.053271758** | **0.000968** |  |
| orange1.1t04970 | Knotted-like homeobox KNOX5 |  |  |  | -1.325313918 | 0.045984 |  |
| Cs2g02930 | Homeodomain-like superfamily protein | 2.801846502 | 0.031857 |  |  |  |  |
| Cs7g03680 | Homeodomain-like superfamily protein isoform 1 | 1.167193324 | 2.72E-05 |  |  |  |  |
| Cs6g21120 | Transcription factor bHLH135-like |  |  |  | 1.145246825 | 3.86E-05 |  |
| Cs6g09670 | Transcription factor bHLH61-like |  |  |  | -2.173369481 | 2.23E-35 |  |
| **Cs5g05300** | **Transcription factor bHLH36-like, partial** | **-1.364792999** | **0.002003** |  | **1.455015442** | **3.10E-20** |  |
| **Cs3g04590** | **Basic helix-loop-helix (bHLH) DNA-binding superfamily protein** | **-2.508785466** | **0.000136** |  | **-3.000286035** | **8.05E-13** |  |
| XLOC_027581 | Myc2 bHLH protein isoform 1 | -1.230769943 | 1.22E-08 |  |  |  |  |
| Cs3g17780 | Transcription factor bHLH96-like | -1.509479691 | 1.58E-11 |  |  |  |  |
| orange1.1t02482 | Transcription factor bHLH36-like, partial | -2.3613259 | 0.000106 |  |  |  |  |
| **Cs3g27630** | **NAC domain transcriptional regulator superfamily protein** | **-1.091526598** | **0.004996** |  | **-1.897134397** | **4.05E-07** |  |
| **Cs6g20980** | **NAC domain protein, IPR003441** | **-1.391010811** | **1.02E-05** |  | **-1.062481177** | **3.92E-07** |  |
| **orange1.1t00587** | **NAC domain protein** | **1.387482202** | **2.20E-05** |  | **-1.399151382** | **0.016898** |  |
| Cs5g18940 | NAC domain protein, IPR003441 |  |  |  | 1.325038817 | 8.21E-05 |  |
| Cs8g14700 | NAC transcription factor 037 | -1.973008167 | 5.75E-10 |  |  |  |  |
| Cs3g19890 | NAC domain protein, IPR003441 | 1.130665825 | 8.35E-11 |  |  |  |  |
| Cs5g19590 | Integrase-type DNA-binding superfamily protein |  |  |  | -2.191962269 | 0.000330 |  |
| **Cs7g04300** | **Integrase-type DNA-binding superfamily protein** | **-3.838860446** | **1.88E-18** |  | **-1.673696512** | **6.73E-05** |  |
| **orange1.1t03822** | **Integrase-type DNA-binding superfamily protein** | **-2.896796915** | **2.20E-08** |  | **1.029507052** | **6.48E-25** |  |
| Cs7g06120 | Integrase-type DNA-binding superfamily protein | -1.208958036 | 3.75E-05 |  |  |  |  |
| **Cs5g06850** | **GRAS family transcription factor** | **1.222268228** | **7.50E-06** |  | **2.241936482** | **6.18E-32** |  |
| Cs3g19370 | GRAS family transcription factor | 1.663859907 | 5.46E-16 |  |  |  |  |
| Cs3g03200 | GRAS family transcription factor | -1.061048142 | 0.012800 |  |  |  |  |
| Cs4g12130 | GRAS family transcription factor isoform 1 | -2.32646213 | 2.25E-19 |  |  |  |  |
| Cs7g07550 | Basic leucine zipper 9-like |  |  |  | -1.032808593 | 1.91E-15 |  |
| Cs7g05140 | Basic-leucine zipper transcription factor family protein, 5 |  |  |  | 1.012483469 | 0.000824 |  |
| Cs7g02230 | Basic-leucine zipper transcription factor family protein isoform 1 | 1.089595024 | 0.001259 |  |  |  |  |
| Cs1g04780 | Trihelix transcription factor GT-2-like | 1.012157616 | 1.46E-05 |  |  |  |  |
| Cs6g08550 | Trihelix transcription factor GT-3b-like |  |  |  | 1.110976777 | 7.35E-24 |  |
| Cs4g15820 | Heat stress transcription factor C-1-like |  |  |  | -1.278785703 | 2.11E-65 |  |
| Cs9g07650 | Heat stress transcription factor A-6b-like isoform 1 | 1.103163328 | 2.73E-05 |  |  |  |  |
| **orange1.1t03944** | **Transcription factor ORG2** | **-2.948030165** | **2.48E-08** |  | **-2.783993858** | **1.28E-10** |  |
| **Cs5g32400** | **Auxin response factor 23-like** | **-4.938662085** | **4.29E-26** |  | **-1.465159323** | **3.78E-07** |  |
| Cs9g04610 | Nuclear transcription factor Y subunit B-5 | 2.221569102 | 4.62E-13 |  |  |  |  |
| Cs5g03650 | Nuclear transcription factor Y subunit C-9 |  |  |  | -1.222733902 | 8.67E-11 |  |
| Cs3g07030 | Nuclear factor Y isoform 2 | 1.118025788 | 0.003308 |  |  |  |  |
| Cs5g17940 | Agamous-like MADS-box protein AGL12-like |  |  |  | 1.133525154 | 0.018648 |  |
| Cs3g25600 | MADS-box protein | 1.432630199 | 0.000324 |  |  |  |  |
| Cs6g21640 | WIN1-like protein |  |  |  | -1.355586287 | 1.54E-10 |  |
| Cs7g03980 | Transcription factor TCP15-like |  |  |  | -1.29002674 | 0.002919 |  |
| Cs4g18910 | Transcription factor RAX2-like | 1.422610962 | 8.07E-09 |  |  |  |  |
| Cs2g15930 | Transcription factor VIP1-like | -1.037800962 | 0.002928 |  |  |  |  |
| Cs8g15600 | Transcription factor FER-LIKE IRON DEFICIENCY-INDUCED TRANSCRIPTION FACTOR | 1.657252766 | 6.45E-19 |  |  |  |  |
| **orange1.1t05396** | **Abscisic acid stress ripening-related protein** | **2.271891894** | **6.07E-32** |  | **-1.039786306** | **4.45E-16** |  |
| Cs3g21500 | Abscisic acid stress ripening-related protein | 1.644988323 | 2.27E-19 |  |  |  |  |
| Cs3g21510 | Abscisic acid stress ripening-related protein | 2.05685532 | 3.42E-30 |  |  |  |  |
| Cs9g01090 | Class 1 knox protein |  |  |  | 1.01481255 | 0.003278 |  |
| orange1.1t02259 | Protein PHR1-LIKE 1-like |  |  |  | -1.033259464 | 0.000124 |  |
| Cs3g23280 | WUSCHEL related homeobox 4 |  |  |  | -1.76131033 | 1.00E-09 |  |
| Cs3g27780 | AT-hook motif nuclear localized protein 29 |  |  |  | -1.128205864 | 6.61E-08 |  |
| Cs5g04850 | DP-E2F-like 1 isoform 1 |  |  |  | 1.028632098 | 3.48E-12 |  |
| Cs1g06690 | HAT dimerisation | 1.684766842 | 0.000161 |  |  |  |  |
| Cs1g24030 | Scarecrow-like protein 4-like | -2.188554775 | 1.20E-27 |  |  |  |  |
| Cs2g01990 | SCL domain class transcription factor | -1.735662891 | 1.65E-10 |  |  |  |  |
| Cs2g11480 | PLATZ transcription factor family protein | -1.407318927 | 0.013889 |  |  |  |  |
| Cs3g25520 | JHL18I08.10 protein isoform 1 | 1.059074743 | 1.47E-09 |  |  |  |  |
| Cs5g01450 | MYC2 | -1.595319928 | 8.06E-05 |  |  |  |  |
| Cs5g06590 | BTB and TAZ domain protein 2 isoform 1 | -2.345539672 | 5.90E-37 |  |  |  |  |
| Cs8g20550 | Two-component response regulator ARR10 | 2.108455562 | 2.42E-17 |  |  |  |  |
| Cs9g16810 | CRT/DRE binding factor | -3.51649164 | 2.47E-20 |  |  |  |  |
| Cs9g18470 | Sigma factor E isoform 1 | 1.303392182 | 2.33E-09 |  |  |  |  |
| XLOC_008604 | Protein RADIALIS-like 6-like | 1.55882412 | 0.037669 |  |  |  |  |
| Cs4g18920 | RNA polymerase sigma factor rpoD | 1.977221694 | 1.98E-12 |  |  |  |  |
| Cs3g18870 | BZIP transcription factor family protein | 1.340441496 | 7.08E-05 |  |  |  |  |
| orange1.1t01637 | PHD finger protein MALE STERILITY 1 | -8.936939696 | 0.000167 |  |  |  |  |

*DEGs shared by the two citrus species were highlighted in bold. FC, fold change; FDR, false discovery rate.*
